# A direct replication and extension of Popp and Serra (2016, experiment 1): better free recall and worse cued recall of animal names than object names, accounting for semantic similarity

**DOI:** 10.3389/fpsyg.2023.1146200

**Published:** 2023-05-18

**Authors:** Eric Y. Mah, Kelly E. L. Grannon, Alison Campbell, Nicholas Tamburri, Randall K. Jamieson, D. Stephen Lindsay

**Affiliations:** ^1^Department of Psychology, University of Victoria, Victoria, BC, Canada; ^2^Department of Psychology, University of Manitoba, Winnipeg, MB, Canada

**Keywords:** adaptive memory, animacy, cued recall, free recall, direct replication

## Abstract

**Introduction:**

Free recall tends to be better for names of animate concepts such as animals than for names of inanimate objects. In Popp and Serra’s 2016 article, the authors replicated this “animacy effect” in free recall but when participants studied words in pairs (animate-animate pairs intermixed with inanimate-inanimate pairs) and were tested with cued recall, performance was better for inanimate-inanimate pairs than for animate-animate pairs (“reverse animacy”). We tested the replicability of this surprising effect and one possible explanation for the effect (semantic similarity).

**Methods:**

Our Experiment 1 was a preregistered direct replication (*N* = 101) of Popp and Serra’s Experiment 1 (mixed-lists condition). In a second preregistered experiment conducted in four different samples (undergraduate *N* = 153, undergraduate *N* = 143, online Prolific *N* = 101, online Prolific/English-as-a-first-language *N* = 150), we manipulated the within-category semantic similarity of animal and object wordlists.

**Results:**

AIn Experiment 1, just as in Popp and Serra, we observed an animacy effect for free recall and a reverse animacy effect for cued recall. Unlike Popp and Serra, we found that controlling for interference effects rendered the reverse animacy effect non-significant. We took this as evidence that characteristics of the stimulus sets (e.g., category structure, within-category similarity) may play a role in animacy and reverse animacy effects. In Experiment 2, in three out of our four samples, we observed reverse animacy effects when within-category similarity was higher for animals and when within-category similarity was equated for animals and objects.

**Discussion:**

Our results suggest that the reverse animacy effect observed in Popp and Serra’s 2016 article is a robust and replicable effect, but that semantic similarity alone cannot explain the effect.

## Introduction

Replicability is a hallmark of science. Direct replications are particularly valuable for assessing the report of new phenomena that are in some way surprising. Here we report a direct replication of an experiment reported by Popp and Serra (2016) having to do with memory for words that name animals (e.g., cat and dog) versus words that name inanimate objects (e.g., chair and hammer), followed by an experiment investigating one possible explanation for the effects observed by Popp and Serra. We begin by describing the context of Popp and Serra’s research, then summarize their findings and explain why we believe it is important to assess their replicability. Then we report our findings and discuss their implications.

### Adaptive memory

According to evolutionary psychology, our perceptual and memory systems are adapted to notice and retain information with high survival relevance (Nairne et al., 2007; for a meta-analysis, see Scofield et al., 2017). Current accounts suggest that survival-relevant information recruits additional processing (e.g., elaboration, deep processing, simulation) that is less extensively used with other non-survival information (Kazanas et al., 2020). This argument of evolutionary psychology led to the development of the *survival processing* experimental paradigm (Nairne et al., 2007). In this paradigm, participants study stimuli in a survival context (or in a non-survival control context) and are subsequently tested on memory for the stimuli. As an example, some participants may be told to imagine that they are in a dangerous grassland and to think about study-list items in terms of their relevance to surviving in such an environment (Kazanas et al., 2020) or instructed to rate stimuli on their survival relevance (Schwartz and Brothers, 2014), whereas subjects in a control condition complete a standard study-test phase or imagine a scenario with low survival relevance (e.g., moving to a new home; Nairne et al., 2007). Recall performance is generally observed to be superior when items are encoded in a survival orientation.

### Animacy and free recall

In studies of survival processing and memory, subjects are instructed to process study-list items in survival-relevant ways or in some other comparably “deep” way that is not related to survival. In a related but distinct line of work (again initiated by James S. Nairne), researchers have compared memory for names or images of animate things (e.g., animals/humans) versus names or images of inanimate objects (e.g., tools/toys). For example, Nairne et al. (2007) sought to examine the effect of animacy on memory by asking participants to free recall a randomly intermixed list of words representing animate (e.g., baby, soldier, duck) and inanimate (e.g., doll, purse, hat) items and discovered that participants recalled more animate than inanimate items. In related work, VanArsdall et al. (2013) demonstrated a corresponding animacy advantage in free recall when nonwords were associated with animate versus inanimate features. That is, when a made-up nonword was paired with a living property (e.g., “FRAV dislikes tomatoes”), free recall for that nonword was better than when it was paired with a non-living property (e.g., “FRAV runs on gasoline”). This “animacy effect” has been well-documented across a variety of designs and tasks, including recall and recognition memory for both word and picture stimuli (Bonin et al., 2014; Scofield et al., 2017).

### Animacy and cued recall

In contrast to the consistent finding of an animacy advantage in free recall and recognition, studies of the animacy effect in paired-associate cued recall have yielded mixed results. Initial findings suggested that participants were better at learning Swahili-English pseudo-vocabulary translations when a Swahili stimulus word was randomly paired with an English animal name compared to when it was matched with an English object name (VanArsdall et al., 2015). Popp and Serra (2016) noted that vocabulary translation tasks such as that used by VanArsdall et al. (2015) differ from other forms of paired-associate learning and that variables that affect one operationalization of paired-associate learning do not necessarily affect other operationalizations. To address this, Popp and Serra’ Experiment 1 examined participants free and cued recall performance for pairs of English words using 84 animal names and 84 inanimate object names matched on a number of relevant features including number of letters, imagability, concreteness, and frequency. When subjects studied and attempted free recall of individual words, performance was better for animate than inanimate words (i.e., an animacy effect in free recall). However, when they studied animate-animate and inanimate-inanimate pairs, subsequent cued recall of targets was better for the inanimate-inanimate pairs (i.e., a reversed animacy effect in cued recall). Popp and Serra reported converging evidence for this reversed animacy effect in two additional experiments (although, in Experiment 2 they obtained ambiguous results in a condition in which one member of the pair was a Swahili word and the other an animate or inanimate English word).

Kazanas et al. (2020) also reported converging evidence for a reverse animacy effect on cued recall using English-Spanish translation pairs. They reported an experiment in which they orthogonally varied orientation (survival vs. controls) and animacy (animate vs. inanimate). English-speaking monolinguals studied recordings of spoken English-Spanish translation pairs (e.g., cat: gato) with varying instructions as to how to think of the words during study. Later, participants were tested on sentence completion, matching, or picture naming. On all of these associative-learning tasks, performance was better for inanimate than animate pairs. The findings of Kazanas et al. contrast with those of VanArsdall et al. (2015)–who, as noted above, observed an animacy advantage with cued recall of English-Swahili translation pairs–but converge with the Popp and Serra (2016) findings.

In contrast with these findings in support of the reverse animacy effect, recent experiments from DeYoung and Serra (2021, Experiments 4 & 5) with similar procedures and same/different wordsets did not yield a reverse animacy effect. In one experiment, the authors failed to replicate the reverse animacy effect with the Popp and Serra (2016) wordset, and in both experiments the authors observed an animacy advantage for cued recall with a new wordset. These results raise several possibilities, the most salient being that (a) the reverse animacy effect is not replicable, and/or (b) wordset-specific characteristics *other than* animacy explain or moderate the effect. We conducted two experiments to address these possibilities. Experiment 1 is a direct replication of Popp and Serra (2016), and Experiment 2 is an extension of their experiment that examines an alternate explanation for the reverse animacy effect in cued-recall.

## Experiment 1

To the best of our knowledge, no direct replication of the Popp and Serra (2016) experiment has been reported. DeYoung and Serra (2021; Experiment 5) used the Popp and Serra (2016) wordset in a cued recall-only experiment and failed to observe an animacy advantage or disadvantage. Kazanas et al. (2020) observed a reverse animacy effect, but their study was far from a *direct* replication of the original Popp and Serra design. As Popp and Serra noted, language-translation tasks differ from other paired-associate learning tasks. Also, Kazanas et al. used only 12 words from each category.

The animacy advantage in free recall has been reported by multiple labs using different sets of materials and a variety of procedures, and while the animacy literature thus far has allowed researchers to hypothesize mechanisms (e.g., mental imagery, attention, semantic features; Bonin et al., 2015, and Xiao et al., 2016), the matter of underlying mechanisms is far from settled. Because most previous animacy-memory studies have shown a memory advantage for animate stimuli, and because proposed underlying theories (i.e., adaptive memory) predict a general animacy advantage, the observation of an animacy *disadvantage* in cued recall warrants verification. If a reverse animacy effect for cued recall proves to be robust, that may help advance our understanding of the mechanisms underlying the relationship(s) between animacy and memory. Thus, the principal aim of this experiment was to replicate the key finding from Popp and Serra’s Experiment 1 (i.e., an animacy effect in free recall paired with a reversed animacy effect in cued recall). We also collected self-report measures regarding participants’ perceptions of the experiment.

### Method

The plans for this experiment were preregistered on the Open Science Framework^1^ with all study materials, the program used to collect the data, details of the procedure, and the specifications for the planned statistical analyses; see https://osf.io/hcp4m. The data and scripts used to process/analyze the data can be accessed at https://osf.io/pbec9/. We also include a report from the Transparency Checklist (Aczel et al., 2020) that may be useful in assessing our preregistration.

### Design

The experiment conformed to a 2 (animacy: animals, objects) × 2 (memory type: free recall, cued recall) within-subjects design, with the main dependent variable being recall accuracy. Popp and Serra tested half of their Experiment 1 subjects with study lists that intermixed names of animals and objects and half with blocked lists (i.e., all animals for one study-test cycle, all objects for another). In our experiment, we used only mixed lists because we were not particularly interested in list type (which had no statistically significant effects in Popp and Serra).

#### Sample size planning

When planning a direct replication for which the primary outcome will be based on null hypothesis significance testing (NHST), it is desirable to test a sufficiently large sample of subjects to attain high statistical power to detect the hypothesized effect, if it is real. Sample-size planning is complicated by the fact that publication bias favors the publication of large effects. Consequently, sample-size plans based on published literature may have low power to detect the average effect of the manipulation in question (Anderson et al., 2017). Additionally, for under-studied effects (such as the reverse animacy effect), there are few effect size estimates upon which to rely. We used Simonsohn’s (2015) “small telescopes” approach, which suggests setting a sample size of 2.5 times that of the to-be-replicated study. According to Simonsohn, this gives the replication about 80% power to reject the null hypothesis of a detectable effect (i.e., an effect that the original study had 33% power to detect). In other words, the “small telescopes” heuristic allows one to power replication studies for effects that would have been minimally detectable in the original study (rather than observed effects, which may be inflated). Following this heuristic, we set our minimum sample at 2.5 times the number of subjects in Popp and Serra (2016), Experiment 1 mixed-list conditions (*N* = 36), at 90 participants. We also preregistered that if more than 90 subjects met the inclusion criteria at the end of the available data-collection period they would be included in our analyses.

### Sample

Participants (*N* = 104) were recruited via our university’s psychology research participation pool. All members of the participation pool were eligible to participate. Age in our sample ranged from 18 to 36 years old (*M* = 21.2, *SD* = 3.8). We did not collect information on gender or ethnicity, but participants drawn from this pool tend to self-identify as female (72%) and European/Caucasian (71%). We asked participants whether they learned English as a first language, second language, or simultaneously with another language: 82% of participants reported English as a first language, 7% reported English as a second language, and 11% reported bilingual English and another language. Participants were compensated with optional extra credit in a psychology course. Data were collected from 104 participants, but three participants were excluded from analysis based on preregistered exclusion criteria and so the final sample size was 101.^2^

### Materials

The computer program used in this study was generously provided by Michael Serra of Popp and Serra (2016). We made a few minor modifications to the instructions and to the informed consent statement and added some self-report items after the main task. The LiveCode (https://livecode.com/) program for the experiment is accessible at https://osf.io/jpd5k. The Popp and Serra program included 84 each of animal and object words, with lists matched on mean number of letters, mental imagery, concreteness, and word frequency. Animal words included mammals, insects, reptiles, amphibians, birds, and fish. Object words included household objects, tools, instruments, clothing, sports-related objects, appliances, and some miscellaneous objects (e.g., anchor, cannon). Readers can refer to the original Popp and Serra article for a detailed description of words used in this experiment and how they were selected.

### Procedure

We posted the study on our university’s online psychology research participation system that students use to sign up and participate in psychology experiments for optional bonus points in certain courses. Students who signed up to participate were given instructions and a Dropbox link to download the experiment program. After completing the experiment remotely on their own computers participants emailed us their data files. The remote/online nature of our replication differed from the original study, and was a result of COVID-19 closures.

The program began by presenting a description of the study and inviting informed consent. Participants were given an option to withdraw from the study on the consent form, in which case the program collected no data. Participants could also withdraw at any point by simply opting not to send us their data. Due to the remote nature of the data collection, we do not know how many participants opted to withdraw. Of our final sample, 53 participants were randomly assigned to perform two free recall study/test blocks followed by two cued-recall study/test blocks; the other 48 did the two types of study/test blocks in the opposite order.

In each study/test block, the program first presented study instructions. For free recall study/test blocks, participants were told that a series of nouns would be presented one at a time and that they would later be asked to recall those words in any order. For cued recall study/test blocks, they were told that a series of pairs of nouns would be presented and that they would then be shown the first word in each pair and invited to recall its partner. The program then presented study items (words or pairs) one at a time for 5 s each, preceded by a 1-s fixation cross. For each participant and for each study list, the program randomly selected 15 words (or word pairs) from the stimulus set without replacement, under the constraint that across the two study lists of a given type there be a total of 15 animal names (or pairs) and 15 object names (or pairs). For instance, if a participant’s first free recall list contained 5 animals and 10 objects, the second free recall list would contain 10 animals and 5 objects^3^. Then the test instructions were presented. For free recall, participants were instructed to type in all the words they could remember from the previously studied list in any order, pressing enter after each word they recalled. For cued recall, participants were informed that the first word of each studied pair would be presented on the computer screen one-at-a-time and that they were to type in the target word of the pair, after which they pressed enter to proceed to the next cue word. For both types of tests, participants could see all of their responses throughout the test. Participants were told they could guess or leave free and cued responses blank, and were given unlimited time to complete each test phase. The program recorded reaction times as a matter of completeness (i.e., time from test start or last word submission to current word submission).

After pressing enter on the last cued recall item on the cued recall test, participants were automatically shown the study instructions for the next study/test cycle. When participants decided that they had completed a free recall test, they clicked a “Finished” button to advance to the next study instructions. This process repeated until the participant had completed all four study/test blocks. Other than minor changes to the instructions and an informed consent statement, the foregoing parts of the procedure were identical to the procedure used by Popp and Serra.

Following the experiment, participants were invited to enter their age in years and to indicate whether or not English was their first language^4^. We then asked questions that assessed participants’ (a) awareness of the animal/object categories, (b) study and test strategies, and (c) perceived relative difficulty of recalling animals versus objects for each type of test (Table 1). Participants were also asked to indicate whether they experienced distractions during the experiment, with the options “No distractions,” “Minor/brief/few distractions,” “A major distraction that affected my ability to pay attention to the experiment.” After answering all of the questions, participants were debriefed and thanked for their participation.

**Table 1:** Self-report strategy questionnaire.

1.	“Please describe the words you studied. That is, what characteristics, properties, or attributes did the words have?”
2.	“Some of the words were names of animals and others were names of inanimate objects. Did you notice that fact when you were studying the words?” [Definitely not/Maybe not/Do not know/Maybe yes/Definitely yes]
3.	“When words were presented one at a time for study, what if anything did you do to try to remember them?”
4.	“When words were presented one at a time for study, did you use the same strategy for animals and objects, or different strategies for animals versus objects? If you used different strategies, please describe them below.”
5.	“When tested on free recall of words that you had studied one at a time, did you find it easier to recall one category of words than the other? Drag the slider below to indicate the relative ease of remembering words from each category” [Objects much easier vs. Animals much easier]
6.	“When words were presented as pairs for study, what if anything did you do to try to remember them?”
7.	“When words were presented as pairs for study, did you use the same strategy for animal pairs and object pairs or different strategies for animal pairs versus object pairs? If you used different strategies, please describe them below.”
8.	“When tested on recall of words that you had studied in pairs, did you find it easier to recall one category of word pair than the other? Drag the slider below to indicate the relative ease of remembering words from each category.” [Objects much easier vs. Animals much easier]

## Results

### Analytic strategy

Our primary analyses were NHST ANOVAs and follow-up *t* tests, mirroring the analyses of the original experiment. However, we supplemented those frequentist analyses with Bayesian analogs (see Supplementary online material Section 1A for more details). We applied these analyses to the following 6 questions. Did we replicate the animacy and reverse animacy effects observed in Popp and Serra (2016), using their scoring method? Did we replicate the animacy and reverse animacy effects observed in Popp and Serra (2016), using a more involved manual scoring method? Does paired-associates interference (e.g., differential benefits of guessing for animals vs. objects) account for the effects we observed? Were most participants aware of animal and object categories, and if so, how specific or general were the categories they perceived? Did category awareness relate to observed animacy effects? What memory strategies did participants use for free and cued recall, and did participants use different strategies for animals and objects (and if so, did this relate to observed animacy effects)? Were participants sensitive to differences in recall difficulty (e.g., animals easier in free recall, harder in cued recall) at a metacognitive level? Did participant self-reports of relative recall difficulty map onto actual performance (i.e., were participants calibrated)?

### Primary analysis

**Figure 1:**
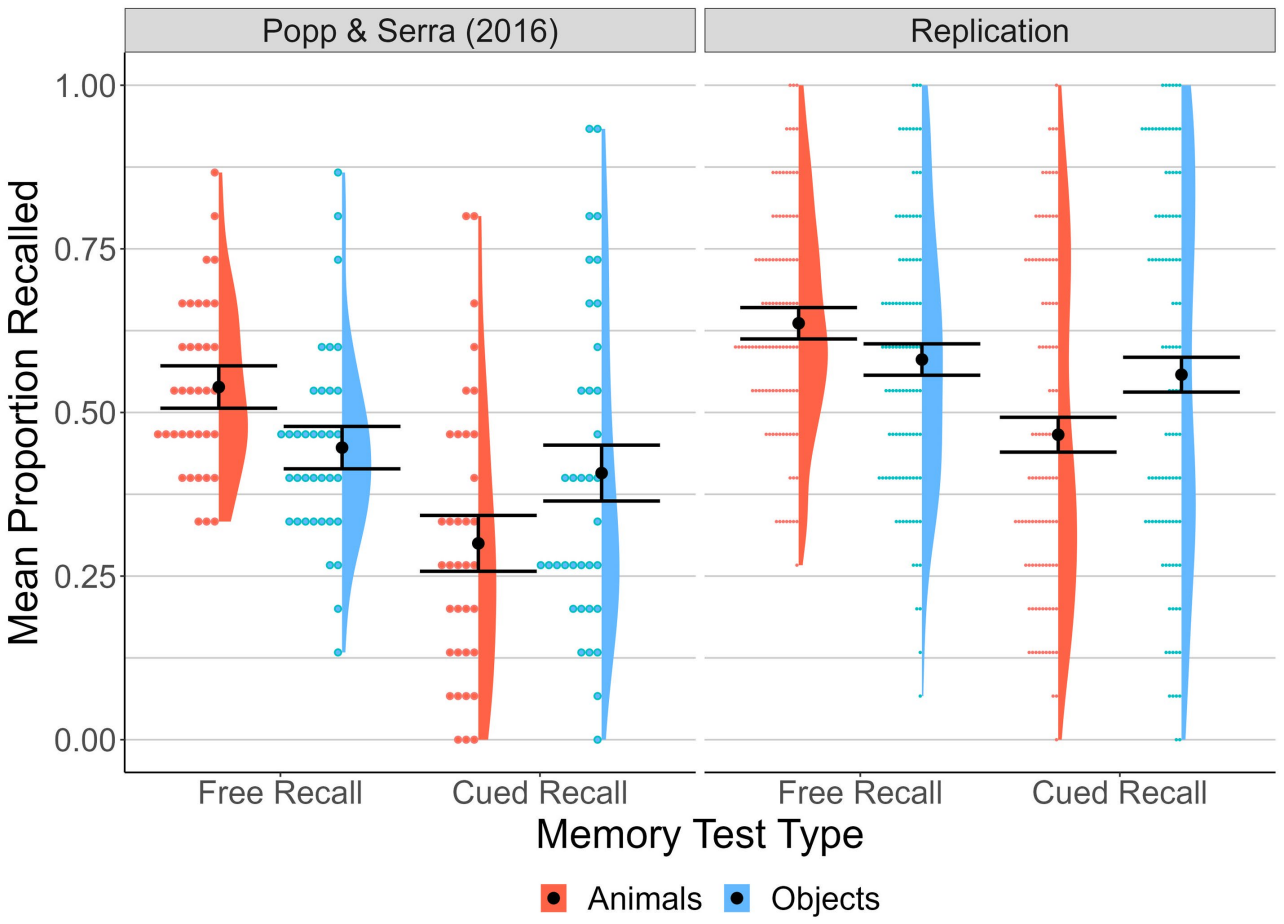
Proportion of targets correctly recalled by Memory Test Type and Animacy. Means, distributions, and individual participant data points for proportion of targets correctly recalled across memory test type and animacy conditions for the original study data (Popp and Serra, 2016, Exp 1, mixed-list condition) and the current replication. Error bars represent 95% within-subject confidence intervals based on the animals versus objects comparison for each memory test type (calculated as per Loftus and Masson, 1994).

We assessed recall accuracy with the scoring method used by Popp and Serra (2016). For free recall, responses that exactly matched a study-list word were automatically counted as correct. All other responses were judged by two independent scorers (blind to type of test) and responses that both scorers judged to be acceptable matches to a study-list word (e.g., “harpsicord” for “harpsichord”) were counted as correct (103 cases, or 3.3% of all free recall trials). When there was disagreement between the two scorers about the match of the response word and a word from the study list (10 cases), a third independent scorer who was blind to the test condition resolved it. Free recall performance was operationalized as the proportion of animal and object words that participants correctly recalled (out of 15 each of studied animal and object words) across the two lists. For cued recall, response words were computer scored: If the first three letters of the response matched the first three letters of the studied target word, the response was counted as correct. Cued recall performance was operationalized as the proportion of animal–animal and object-object pairs that participants correctly completed (out of 15 each of studied animal–animal and object-object pairs) across the two lists of pairs. Figure 1 shows the means, distributions, and data points for each condition.

Proportion of targets accurately recalled was analyzed with a 2 (animacy: animals, objects) × 2 (memory type: free recall, cued recall) within-subjects ANOVA. We also evaluated main and interaction effects via Bayes Factor (BF)^5^ analysis. Consistent with the results of Popp and Serra (2016), mean proportion of targets accurately recalled was higher for free recall (*M* = 0.61, *SD* = 0.19) than cued recall (*M* = 0.51, *SD* = 0.28), *F*(1, 100) = 21.04, *p* < 0.001, η*_p_*^2^ = 0.17. The corresponding BF for this analysis was >100; “extreme” evidence in favor of a memory type main effect (Wagenmakers et al., 2011). Also replicating Popp and Serra, the main effect of animacy was not significant, *F*(1, 100) = 1.68, *p* = 0.20, η*_p_*^2^ = 0.02 (BF = 0.6; anecdotal evidence against an effect), and the interaction between animacy and memory type was significant, *F*(1, 100) = 41.70, *p* < 0.001, η*_p_*^2^ = 0.29 (BF > 100). Paired-samples *t* tests^6^ (Bonferroni corrected) were used to examine the interaction. This revealed that mean free-recall proportion correct for animals (*M* = 0.64, *SD* = 0.17) was better than that for objects (*M* = 0.58, *SD* = 0.21), *t*(100) = 3.24, *p* = 0.002, *d*_z_^7^ = 0.32 [0.12, 0.52]. By contrast, mean cued-recall proportion correct was better for object pairs (*M* = 0.56, *SD* = 0.29) than for animal pairs (*M* = 0.47, *SD* = 0.25), *t*(100) = 4.84, *p* < 0.001, *d*_z_ = 0.48 [0.27, 0.69].

### Alternative analyses

We explored an alternate scoring procedure in which cued recall responses (as well as free recall responses) were scored manually. As detailed in Supplementary online material (Section 1B), results with this measure mirrored those reported above. We also report in Supplementary online material (Section 1C) an unplanned 2 (Animacy) × 2 (Memory Test Type) × 2 (Test Order) × 2 (Study/Test Block) ANOVA to examine all test-related effects. We coded and analyzed qualitative self-reported recall strategy data, but found no clear relationships between strategy use and animacy/reverse animacy effects (Supplementary online material 1F.4). We also analyzed self-reported recall difficulty, and found that participants showed some (but imperfect) metacognitive awareness of animacy/reverse animacy effects (e.g., objects rated as more difficult than animals, moreso for cued recall; mixed evidence for a relationship between reported recall difficulty and actual performance; Supplementary online material 1F.5). Finally, we conducted several exploratory analyses of reaction time (e.g., reaction times for correct answers vs. commission errors vs. omission errors)—these can be found in Supplementary online material 1F.4 (Section 1D). Below, we briefly discuss two additional alternative analyses relevant to potential explanations for the reverse animacy effect–paired-associates interference and category awareness data.

### Paired-associates interference

Popp and Serra (2016) noted that subjects might report words that they recalled as guesses. Even a word presented on the study list might be generated as a guess (e.g., if it was not encoded at study). Moreover, Popp and Serra speculated that their animate names might be a “narrower,” more closely associated set than their inanimate names. That would promote guessing of studied animate names, relative to guessing of studied inanimate names. On the free recall test, such guesses would inflate performance of animate names despite being “lucky intrusions.” On the paired-associates recall test, in contrast, guessing would be less helpful, and might even interfere with correct report of animate names. For one thing, most studied words generated as paired-associate guesses would have been studied with a different cue word. For another, having a guessed word come to mind might interfere with recall of the target. Popp and Serra referred to this as paired-associate interference, and explored the possibility of interference via an exploratory analysis in which any incorrect animal/object word recalled in place of a correct animal/object target was counted as correct, irrespective of whether the incorrect word was studied or not. For example, if a participant studied the cue-target pair “glasses – motorcycle” but then responded to the test cue “glasses” with the previously non-studied “car,” this was counted as correct. Popp and Serra found that the cued recall reverse animacy effect was still present in their overall sample when interference responses (i.e., same-category commission errors) were counted as correct.

We applied a similar analysis to our own data, and found that when treating same-category cued recall commission errors as correct, the reverse animacy effect was no longer significant (with Bayesian evidence in support of *no* effect) in both our dataset and in the restricted mixed-lists condition of Popp & Serra. Results were similar when only treating *studied* same-category commission errors as correct (see Supplementary online material Section 1E for detailed results for these analyses). Although these results seem to suggest an interference account for the reverse animacy effect, even with the liberal scoring criterion mean cued-recall proportion correct was not better for animals than objects. That is consistent with Popp and Serra’s argument that interference and category size effects do not fully explain differential animacy effects in free versus cued recall.

#### Category awareness

To further explore potential mechanisms underlying the animacy/reverse animacy effects, we coded and analyzed qualitative data from the post-study survey (Table 1). The final coding scheme can be found in Supplementary online material (Section 1F.1). Although most participants reported awareness of animal and object categories (85%, Supplementary online material Section 1F.2), casual inspection of participants’ open-ended descriptions of the materials suggested a difference in the *granularity* or *specificity* of category awareness (e.g., whether participants tended to mention animals as a general or superordinate category, but used more specific subordinate subcategories for objects). We therefore coded for specificity, separately for animals and objects (see Supplementary online material Section 1F.1 for details on the coding scheme). The most striking findings here were: (a) 76% of participants reported *only* a superordinate category for animals while only 42% of participants reported *only* a superordinate category for objects (comparing these proportions, 𝜒^2^(1) = 23.64, *p* < 0.001), and (b) No participants reported *only* a subordinate category for animals, compared to 21% of participants who did for objects (comparing these proportions, 𝜒^2^(1) = 21.26, *p* < 0.001). Proportions of responses in the other categories can be found in Supplementary online material (Section 1F.2).

Although the magnitude of the reverse animacy effect in participants who indicated an awareness of a general object category versus participants who did not did not significantly differ (see Supplementary online material Section 1F.3), these data at least suggest that animal and object categories were perceived differently.

## Discussion

On average, our participants obtained somewhat better scores than the Popp and Serra (2016) subjects, but our primary analyses yielded results that closely paralleled theirs. Free recall was better for animal names than object names whereas cued recall was better for object-object pairs than for animal–animal pairs. These results suggest that, as proposed by Popp and Serra (2016) and Kazanas et al. (2020), the relationship between animacy and memory performance is moderated by some unknown factor(s) related to the type of memory task.

On a cued recall test, nontarget words may sometimes come to mind in response to recall cues (based on semantic and/or implicit memory). Popp and Serra (2016) raised the possibility that nontarget animal names might come to mind in response to animal cue words more often than nontarget object names come to mind in response to object cue words. That might occur if the animal category was more salient and/or narrower than the object category. If so, then nontarget animal names might interfere with retrieval of target animal names more often than nontarget object names interfere with retrieval of target object names. To assess the role of such interference, Popp and Serra re-analyzed their data with a liberal scoring criterion in which any within-category response was treated as correct. They reported an analysis of cued recall accuracy as indexed by this liberal criterion among subjects for whom category was blocked (a condition we did not include in our experiment) as well as subjects for whom categories were mixed. In that analysis, the reverse animacy effect was significant, suggesting that interference alone could not account for the reverse animacy effect on cued recall. But in our larger sample, the reverse animacy effect was not significant with this liberal scoring criterion, and Bayesian analysis provided modest support for the null hypothesis. Also, although Popp and Serra reported no significant List (themed vs. mixed) × Animacy (animal vs. object) interaction on cued-recall proportion correct with liberal scoring, the reverse animacy effect was directionally larger in their themed list than in their mixed list condition. We found that an analysis restricted to the latter yielded the same outcome as our data: Evidence for the absence of an animacy effect on cued recall accuracy with liberal scoring in the mixed-lists condition.

Our post-experiment questions regarding subjects’ perceptions yielded some interesting findings. For one thing, when asked to describe the words our participants much more often mentioned a general animal category than a general object category. Also, few of our participants mentioned only subordinate categories of animals (e.g., “mammals”) whereas more of them mentioned only subordinate object categories (e.g., “tools”) without reference to a superordinate “object” category. These results provide further support for the idea that animals (generally or the ones used in the current stimulus set) represent a more cohesive and singular category than objects. As mentioned previously, it is possible that a tighter category structure for animals benefited free recall but hampered cued recall, while reduced awareness of a general objects category may have hampered free recall but benefited cued recall (due to reduced interference). We investigated this possibility–a category-structure-based explanation for the reverse animacy effect–in Experiment 2.

## Experiment 2

The liberal scoring data from Popp and Serra (2016) and our study suggest that the “reverse animacy effect” might better be described as a “reduced animacy due to interference effect.” Additionally, our exploratory analyses of perceived category specificity suggest that the animal and object categories in the current stimulus set have different semantic structures. These findings also raise the possibility that both the animacy effect in free recall and the reverse animacy effect in cued recall may have more to do with differences in the semantic structure of the two categories (either in general or in the particular items selected by Popp and Serra and by Kazanas et al., 2020) than with the evolutionary significance of animal names. We do not think these researchers deliberately crafted lists with different similarity structures, but neither did they (or we) explicitly measure or control for category size/structure. Experiments directly controlling for or manipulating category semantic structure are necessary to adjudicate between semantic-structure-based explanations for animacy effects (e.g., the *overlapping semantic features hypothesis*; McRae et al., 1997; Xiao et al., 2016) and survival-processing-based accounts (e.g., the *animate monitoring hypothesis*; New et al., 2007).

One way to measure the semantic structure of wordsets is Latent Semantic Analysis (LSA; Landauer and Dumais, 1997). LSA is a method for computing distributional semantics that involves computing the co-occurrence of words across large sets of documents. Words that co-occur often in the same contexts across multiple documents are assumed to be highly similar (Landauer et al., 1998). Validating this assumption are experiments showing correlations between LSA-based measures of similarity and behavioral results (e.g., priming, sentence completion times; Günther et al., 2016).

**Figure 2:**
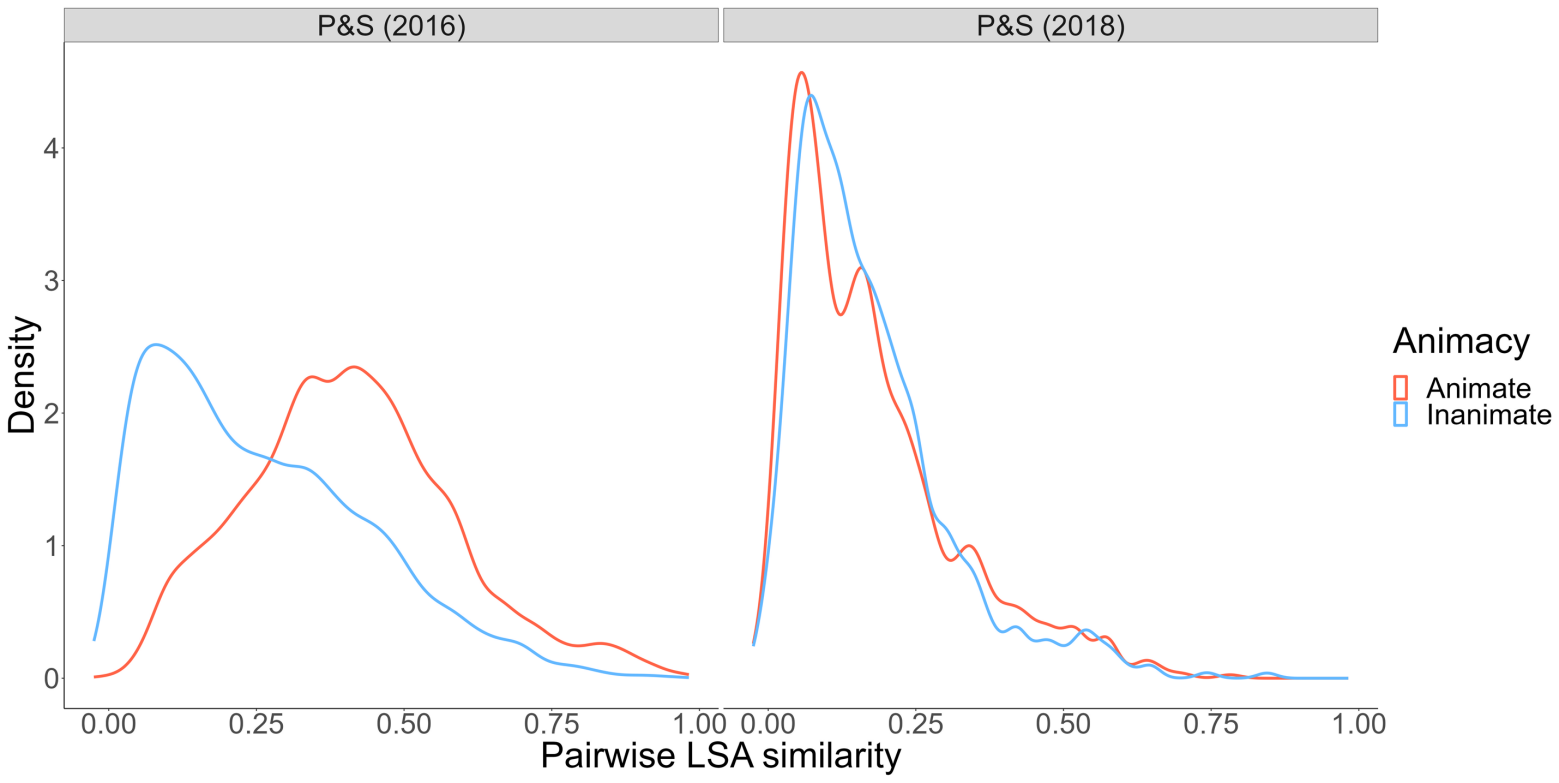
Pairwise semantic similarity from Popp and Serra (2016, 2018) wordsets. Pairwise semantic similarity indexed via LSA cosine similarity, calculated using the LSAfun R package and the English LSA 100 k space with 300 dimensions (Günther et al., 2015).

Using word representations derived with Latent Semantic Analysis as applied to a large document corpus (Günther et al., 2015), we examined wordsets that have been used to test the reverse animacy effect (Popp and Serra, 2016; DeYoung and Serra, 2021). Specifically, we simulated 10,000 animal- and object-word pairs from each wordset and computed pairwise semantic similarities. The distributions of word-to-word similarity are shown in Figure 2.

As shown, the within-category similarities between animate words in the Popp and Serra (2016) animate versus inanimate wordsets (where a reverse animacy effect was observed) is striking. Animate cues and targets were more similar to one another than inanimate cues and targets. Conversely, in the Popp and Serra (2018) wordset (where a reverse animacy effect was *not* observed; DeYoung and Serra, 2021), within-pair similarity was generally lower and, more critically, roughly equal for animates and inanimates. These results, along with our interference analyses and qualitative data on people’s category awareness data from Experiment 1, point increasingly toward a semantic structure-based explanation for the reverse animacy effect. Such an explanation is also consistent with the *overlapping semantic feature* hypothesis (McRae et al., 1997) that suggests animals may be more memorable than objects because animals share more overlapping features (e.g., fur, four legs, teeth) relative to objects, which tend to have wider-ranging features (e.g., features of different musical instruments such as trumpets and guitars have less featural overlap). This hypothesis is supported by studies showing benefits of greater feature and neural global pattern overlap for subsequent memory (Ilic et al., 2013; Xiao et al., 2016). Overlapping semantic features can explain animacy and reverse animacy effects by drawing on the *spreading-activation* theory of semantic memory (Collins and Loftus, 1975) that posits concepts activate other semantically-related concepts in memory proportional to the degree of relatedness. If animal words share more overlapping features, the semantic network will contain stronger links (and vice versa for object words) that benefit free recall of animals due to increased activation of related studied animal targets. However, stronger links might harm performance in cued recall because a given animal cue will activate more related non-target concepts. This explanation is consistent with the results of Popp and Serra (2016) and our Experiment 1, in which there was a clear performance cost in cued recall of animals relative to free recall of animals (whereas free recall and cued recall performance for objects was quite similar).

Of course, semantic similarity lay at the center of these theories and explanations. Thus, we set out to directly test these ideas by comparing animacy and reverse animacy effects in animal versus object free and cued recall with new wordsets that we designed to control pairwise similarity relationships. Specifically, we created one “animals-more-similar” wordset in which listwise/pairwise within-category similarity was higher, on average, for animals than objects (like the Popp and Serra, 2016 wordset), and one “equal” wordset in which within-category similarity was equal, on average, for animals and objects (like the Popp and Serra, 2018 wordset). We initially set out to make an “objects-more-similar” wordset but, perhaps tellingly, were unable to do so. We return to this point in the Discussion.

**Figure 3:**
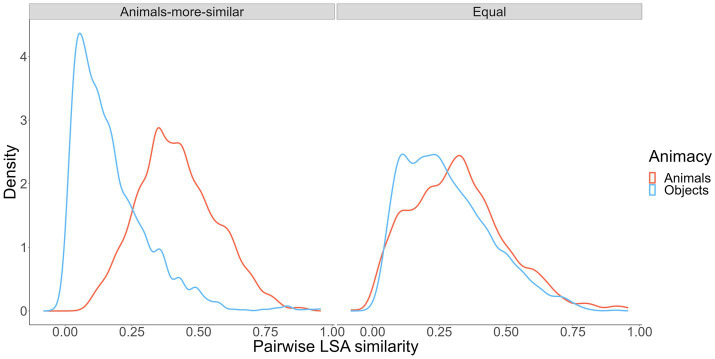
Pairwise semantic similarity for simulated pairs from our experimental conditions.

To create our wordsets, we used a random, iterative word-sampling process to choose words to meet the similarity conditions we wanted to satisfy but that also kept other salient word characteristics as similar as possible across categories (i.e., word frequency, age of acquisition, context diversity, imageability; Madan et al., 2010; Madan, 2021).^8^ Ultimately, we generated two wordsets. The “animals-more-similar” wordset included 48 animal words and 48 object words, with animal-to-animal semantic similarity higher than object-to-object similarity. The “equal” wordset included 48 animal words and 48 objects words, with animal-to-animal and object-to-object semantic similarity roughly equated. The wordsets were mutually exclusive except for 17 animal words and 3 object words that appeared in both wordsets. The similarity profiles in the two wordsets are shown in Figure 3.

Using these wordsets with the Popp and Serra (2016) paradigm, we expect to replicate Popp and Serra (2016) in the animals-more-similar wordset such that participants tested on the “animals-more-similar” wordset will show an animacy effect in free recall but a reverse animacy effect in cued recall (Hypothesis 1). Second, we expect to eliminate the reverse animacy effect in the equal wordset such that we will observe a within-category similarity × animacy interaction for cued recall (Hypothesis 2). Finally, we had no specific predictions about how the “equal” wordset might affect the free recall animacy effect. But one might expect to observe that effect if there is something “special” about the animate words (above and beyond within-category similarity) that eases free recall.

### Methods

We tested these hypotheses in four different samples: two university undergraduate samples and two online samples via Prolific.co. The plans and hypotheses for the latter three samples were preregistered on the Open Science Framework with (a) all study materials, (b) the final version of the program used to collect the data, (c) details of the procedure, and (d) the specifications for the planned statistical analyses^9^ (see https://osf.io/t7qfa). The data and scripts used to process/analyze the data can be accessed at https://osf.io/k4emy/.

### Design

The experiment took the form of a 2 (animacy: animals, objects) × 2 (memory type: free recall, cued recall) × 2 (within-category similarity: animals-more-similar, equal) mixed design. The first two factors were manipulated within-subjects and the third between-subjects. The primary dependent variable was, once again, recall accuracy.

#### Sample size planning

We conducted power simulations using cell mean estimates obtained in Experiment 1 as well as two hypothetical effects (“small” and “large”) of our similarity manipulation for Hypothesis 2. These simulations suggested that we would need 150 ≤ *N* ≤ 500 to detect effects of interest (for the “large” and “small” effects respectively; see the preregistration for more details). For our first sample (undergraduate), we set a target *N* = 150. To maximize the efficiency of sampling for the subsequent samples, we chose to adopt a sequential Bayesian approach (Schönbrodt et al., 2017) whereby we set a minimum testing *N* = 100 and tested our core hypotheses via Bayes Factors at this threshold and at each additional *n* = 10 subsequent participants, up to a maximum total *N* = 150. At each sequential testing threshold, we tested three effects of interest via model-comparison Bayes Factors: the free recall animacy effect in the “animals-more-similar” condition, the cued recall reverse animacy effect in the “animals-more-similar” condition, and the cue recall reverse animacy effect in the “equal” condition. Effects were tested via comparison of a model with the effect of interest to a model without the effect of interest. If evidence in favor or against all three effects exceeded a Bayes Factor of 3, we would terminate data collection, otherwise continue to the next threshold.

### Sample

We collected four different samples for Experiment 2: two undergraduate samples, one online (Prolific.co) sample including all people who reported English fluency, and a fourth sample from online (Prolific.co) restricted to people who reported English fluency and English as a first language (post exclusion *N*s = 150, 143, 101, and 150 respectively). Additional information about the four samples including information about exclusions based on preregistered exclusion criteria (increased from Experiment 1), condition assignments, and demographics can be found in Supplementary online material 2X.

Our objectives in collecting these four samples were to (a) test the generality of effects across different populations and (b) self-replicate our results to ensure internal as well as external convergence^10^.

### Materials

The computer program was unchanged from Experiment 1, except for the following: First, we replaced the Popp and Serra wordset with our new wordsets (“animals-more-similar” and “equal”), to which participants were randomly assigned. Second, we increased the number of words/word-pairs per list to 16 to allow for equal numbers of animal/object words/pairs. Third, For samples 2–4, we ensured that each study list contained 8 animal and 8 object words/pairs. Fourth, we added a line to the cued recall instructions to discourage fast skips: “It is OK to leave items blank if you cannot recall the target word, but please make an effort to recall each target, do not just quickly skip through.” Lastly, we added debriefing/exclusion questions to evaluate cheating (“Did you take notes?”), words understood (0, 25, 50, 75, 100%), and general experiences (general comments, technical difficulties). The LiveCode (https://livecode.com/) program for the experiment is accessible at https://osf.io/khsj9.

### Procedure

Aside from the changes noted above, the procedure was the same as in Experiment 1. In addition to recruiting participants via the University research pool, we posted our study online at Prolific.co, where eligible online participants who took part in the +/− 30-min experiment received $6.00 USD.

## Results

### Analytic strategy

As our sampling plan depended on sequential Bayesian tests, our primary analyses were Bayesian linear mixed-effects models, though we supplemented these analyses with NHST ANOVAs and *t*-tests to facilitate comparison of our results to the original Popp and Serra (2016) work.

Our hypothesized interactions were (a) an animacy by memory type interaction in the “animals-more-similar” condition (i.e., free recall animacy effect paired with a cued recall reverse animacy effect) and (b) a within-category similarity by animacy interaction for cued recall (i.e., reduced/eliminated reverse animacy effect in the “equal” condition). At the granularity of individual cells/conditions, our hypothesized pairwise comparisons of interest, and the basis for our sequential sampling, were (a) an animacy effect in the “animals-more-similar” condition, (b) a cued recall reverse animacy effect in the “animals-more-similar” condition, and (c) *no* cued recall reverse animacy effect in the “equal” condition. To evaluate each of these effects, we conducted Bayes Factor model comparisons of a model with that effect to a model without that effect.

For Experiment 2, accuracy measures were manually coded. That is, verbatim correct answers were counted as correct in addition to misspelled responses that were manually coded (See Supplementary online material Section 1B for details regarding this coding procedure and Supplementary online material Section 2E for manual coding statistics for each sample). Overall, the number of manually corrected responses was small (no more than 5% of total responses).

### Primary analysis

To test our replication of Popp and Serra (2016) in the animals-more-similar wordset, we conducted a 2 (animacy: animals, objects) × 2 (memory type: free recall, cued recall) ANOVAs/Bayesian linear models in the “animals-more-similar” condition. In all samples, the hypothesized interaction was observed (see Table 2 for more details). To test our second hypothesis that we would eliminate the reverse animacy effect in the “equal” wordset, we conducted a 2 (animacy: animals, objects) × 2 (within-category similarity: animals-more-similar, equal) ANOVAs/Bayesian for cued recall only. In all samples (and combining across samples), significant interactions and “extreme” Bayesian evidence (Jeffreys, 1961; BF > 100) supported our first hypothesis. For our second hypothesis, in three out of the four samples the hypothesized interaction was not observed, with moderate Bayesian evidence (BF10 < 0.33) against an interaction in two of those samples (Undergraduate 1 & 2), and anecdotal evidence (1 > BF10 > 0.33) against an interaction in the remaining sample (Prolific EFL). When combining all samples, the interaction was non-significant and Bayesian evidence against was strong (BF > 10). Table 2 lists statistics for these analyses in all samples (see Supplementary online material 2C for individual experiment figures depicting the raw data similar to Figure 1):

**Table 2:** Experiment 2 interaction hypothesis tests.

	H1: Animacy and reverse animacy effects in the “animals-more-similar” condition		H2: Reverse animacy effect in the “animals-more-similar” but not “equal” condition	
Sample	Interaction BF10	Interaction NHST	Interaction BF10	Interaction NHST
Undergraduate 1	2.89 * 10 ^**8**^	*F*(1, 67) = 24.04, ***p* < 0.001**, η^2^_p_ = 0.26	**0.28**	*F*(1, 148) = 0.46, *p* = 0.50, η^2^*_p_* < 0.001
Undergraduate 2	1.48 * 10 ^**10**^	*F*(1, 71) = 52.17, ***p* < 0.001**, η^2^_p_ = 0.42	**0.29**	*F*(1, 141) = 0.68, *p* = 0.41, η^2^*_p_* = 0.005
Prolific	9.90 * 10 ^**6**^	*F*(1, 54) = 12.02, ***p* = 0.001**, η^2^_p_ = 0.18	**6.03**	*F*(1, 99) = 3.34, *p* = 0.07, η^2^_p_ = 0.03
Prolific (EFL)	5.94 * 10 ^**7**^	*F*(1, 78) = 14.55, ***p* < 0.001**, η^2^_p_ = 0.16	0.42	*F*(1, 148) = 0.007, *p* = 0.93, η^2^_p_ < 0.001
Combined	2.11 * 10 ^**23**^	*F*(1, 273) = 91.32, ***p* < 0.001**, η^2^_p_ = 0.25	**0.06**	*F*(1, 542) = 0.35, *p* = 0.55, η^2^_p_ < 0.001

The H1 interaction refers specifically to the 2 (animacy: animals, objects) × 2 (memory type: free recall, cued recall) within-subjects interaction in the “animals-more-similar” condition, with a significant interaction providing support for our hypothesis. The H2 interaction refers specifically to the 2 (animacy: animals, objects) × 2 (within-category similarity: animals-more-similar, equal) within-between interaction for cued recall only, with a significant interaction providing support for our hypothesis. BF10 values are Bayes Factors indicating the ratio of support for the interaction model over a null model with no interaction (i.e., values above 1 provide support for an interaction, values below 1 provide support against an interaction). Bolded values indicate BFs or p-values that exceeded our preregistered critical values.

**Figure 4:**
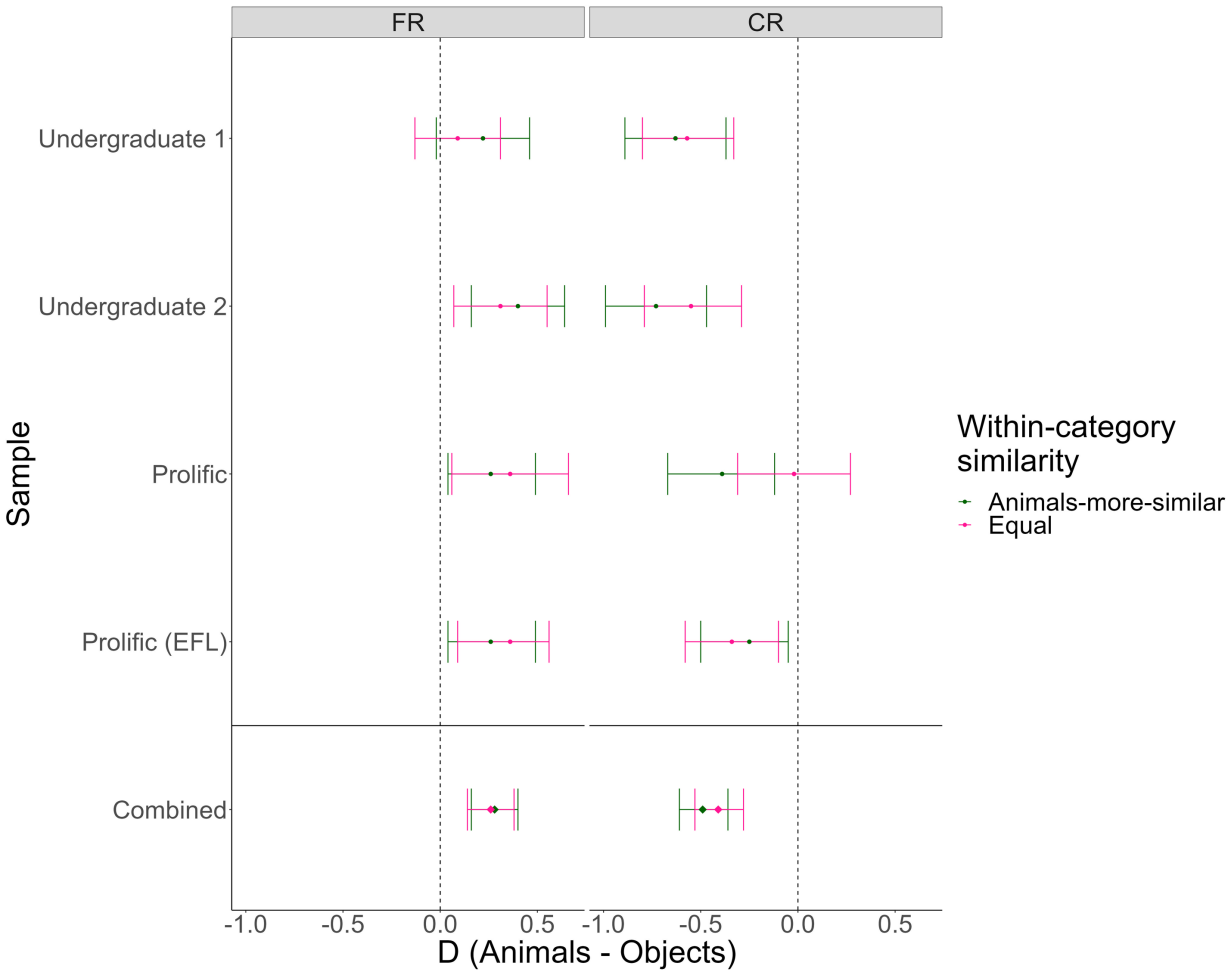
Forest plot of animacy effects by condition and test type in Experiment 2. FR = Free recall, CR = Cued recall. Error bars = 95% CIs on the within-subjects Cohen’s *d_z_*s. Points to the right of the dotted lines represent animacy effects, points to the left of the lines represent reverse animacy effects.

We observed consistent evidence for Hypothesis 1 (Animacy and reverse animacy effects in the “animals-more-similar” condition), replicating the basic Popp and Serra results pattern in our “animals-more-similar” condition. In three out of the four samples (Undergraduate 1 & 2, Prolific EFL), we found evidence *against* Hypothesis 2 (Reverse animacy effect in the “animals-more-similar” but not “equal” condition), suggesting that in these samples, semantic similarity did not modulate the reverse animacy effect. The exception was in our first Prolific sample, where the reverse animacy effect was eliminated in the “equal” semantic similarity condition, with moderate Bayesian evidence for an interaction. As these analyses were conducted using manually coded accuracy, we also conducted versions of these analyses using verbatim/automatic accuracy, with similar results (see Supplementary online material Section 2F). In Table 3, we show the Bayes Factors, *t* tests, and Cohen’s *d_z_* values for all pairwise animal-object comparisons in Figure 4, we visualize the pairwise effects in a forest plot.

**Table 3:** Experiment 2 pairwise animal-object comparison tests.

	“Animals-more-similar” condition				“Equal” condition			
	FR		CR		FR		CR	
Sample	BF10	*t*-test	BF10	*t*-test	BF10	*t*-test	BF10	*t*-test
Undergraduate 1	1.12	*t*(67) = 1.78, *p* = 0.08 / 0.10, *d_z_* = 0.22 [−0.02, 0.46]	2.61 * 10 ^**4**^	*t*(67) = 5.18, ***p* < 0.001**, *d_z_* = −0.63 [−0.89, −0.37]	**0.01**	*t*(81) = 0.83, *p* = 0.41 / 0.49, *d_z_* = 0.09 [−0.13, 0.31]	8.31 * 10 ^**7**^	*t*(81) = 5.14, ***p* < 0.001**, *d_z_* = −0.57 [−0.80, −0.33]
Undergraduate 2	**6.34**	*t*(71) = 3.37, ***p* = 0.001 / 0.003**, *d_z_* = 0.40 [0.16, 0.64]	9.08 * 10 ^**5**^	*t*(71) = 6.21, ***p* < 0.001**, *d_z_* = −0.73 [−0.99, −0.47]	**11.09**	*t*(70) = 2.63, ***p* = 0.01**, *d_z_* = 0.31 [0.07, 0.55]	6.48 * 10 ^**5**^	*t*(70) = 4.60, ***p* < 0.001**, *d_z_* = −0.55 [−0.79, −0.29]
Prolific	**10.24**	*t*(54) = 2.00, *p* = 0.05, *d_z_* = 0.27 [0.001, 0.54]	**9.93**	*t*(54) = 2.91, ***p* = 0.01**, *d_z_* = −0.39 [−0.67, −0.12]	**10.19**	*t*(45) = 2.46, ***p* = 0.02**, *d_z_* = 0.36 [0.06, 0.66]	**0.02**	*t*(45) = 0.15, *p* = 0.88 / 1, *d_z_* = −0.02 [−0.31, 0.27]
Prolific (EFL)	2.81	*t*(78) = 2.35, ***p* = 0.02 / 0.03**, *d_z_* = 0.26 [0.04, 0.49]	**4.96**	*t*(78) = 2.47, ***p* = 0.02**, *d_z_* = −0.25 [−0.50, −0.05]	**23.18**	*t*(70) = 2.75, ***p* = 0.007**, *d_z_* = 0.36 [0.09, 0.56]	**4.65**	*t*(70) = 2.90, ***p* = 0.005 / 0.008**, *d_z_* = −0.34 [−0.58, −0.10]
Combined	**6,705.42**	*t*(273) = 4.70, ***p* < 0.001**, *d_z_* = 0.28 [0.16, 0.40]	4.31 * 10 ^**11**^	*t*(273) = 8.10, ***p* < 0.001**, *d_z_* = −0.49 [−0.61, −0.36]	**2,238.76**	*t*(269) = 4.32, ***p* < 0.001**, *d_z_* = 0.26 [0.14, 0.38]	1.16 * 10 ^**11**^	*t*(269) = 6.68, ***p* < 0.001**, *d_z_* = −0.41 [−0.53, −0.28]

FR = Free recall, CR = Cued recall. Each cell refers to a comparison of memory performance for animals and objects (positive Cohen’s d_z_ effect sizes = better memory performance for animals than objects). BF10 values are Bayes Factors indicating the ratio of support for a model with an animal-object difference relative to a model with no difference (i.e., values above 1 provide support for a difference, values below 1 provide support against a difference). Bolded values indicate BFs or p-values that exceeded our prespecified critical values.

Importantly, in all samples except the third, the cued recall reverse animacy effect was significant and supported by moderate-or-greater Bayesian evidence in both similarity conditions. The free recall animacy effect was less consistent (significant in only 4/8 cells), but was always in the expected direction and significant and supported by extreme Bayesian evidence when combined across samples. Similar results were obtained for verbatim/automatic accuracy (see Supplementary online material Section 2F).

## Discussion

Overall, our results do not support a “semantic similarity” explanation for the reverse animacy effect. The majority-English as a Second Language sample in which we did find support for such an explanation might suggest that first-language status interacts with similarity in some way. There is some evidence that the structure of semantic networks differ between monolinguals and bilinguals (Bilson et al., 2015), but we are hesitant to draw any conclusions about language status on the basis of only one sample. Why then did semantic similarity not influence animacy and reverse animacy effects?

One possibility is that our manipulation was not strong enough. If the reader recalls that we created one wordset in which animal cue-target pairs had (on average) higher within-pair semantic similarity than did cue-target object pairs, and one wordset in which animal and object pairs had (on average) equivalent within-pair semantic similarity. This manipulation was successful in the sense that animal and object word similarity distributions appeared as intended in all samples (see Supplementary online material Section 2D). Both our primary basis for estimating word similarity (LSA) and an additional/newer similarity measure that we computed after the fact (Global Vectors for Word Representation (GloVe)^11^; Pennington et al., 2014) were generally related to memory accuracy (i.e., higher similarity = higher cued recall accuracy, see Supplementary online material Section 2D). However, even in our “equal” similarity condition, semantic similarity was slightly higher for animal–animal than object-object word pairs. It could be that a more forceful difference (e.g., higher within-pair similarity for objects vs. animals) is required to observe statistically corroborated differences in the reverse animacy effect.

**Figure 5:**
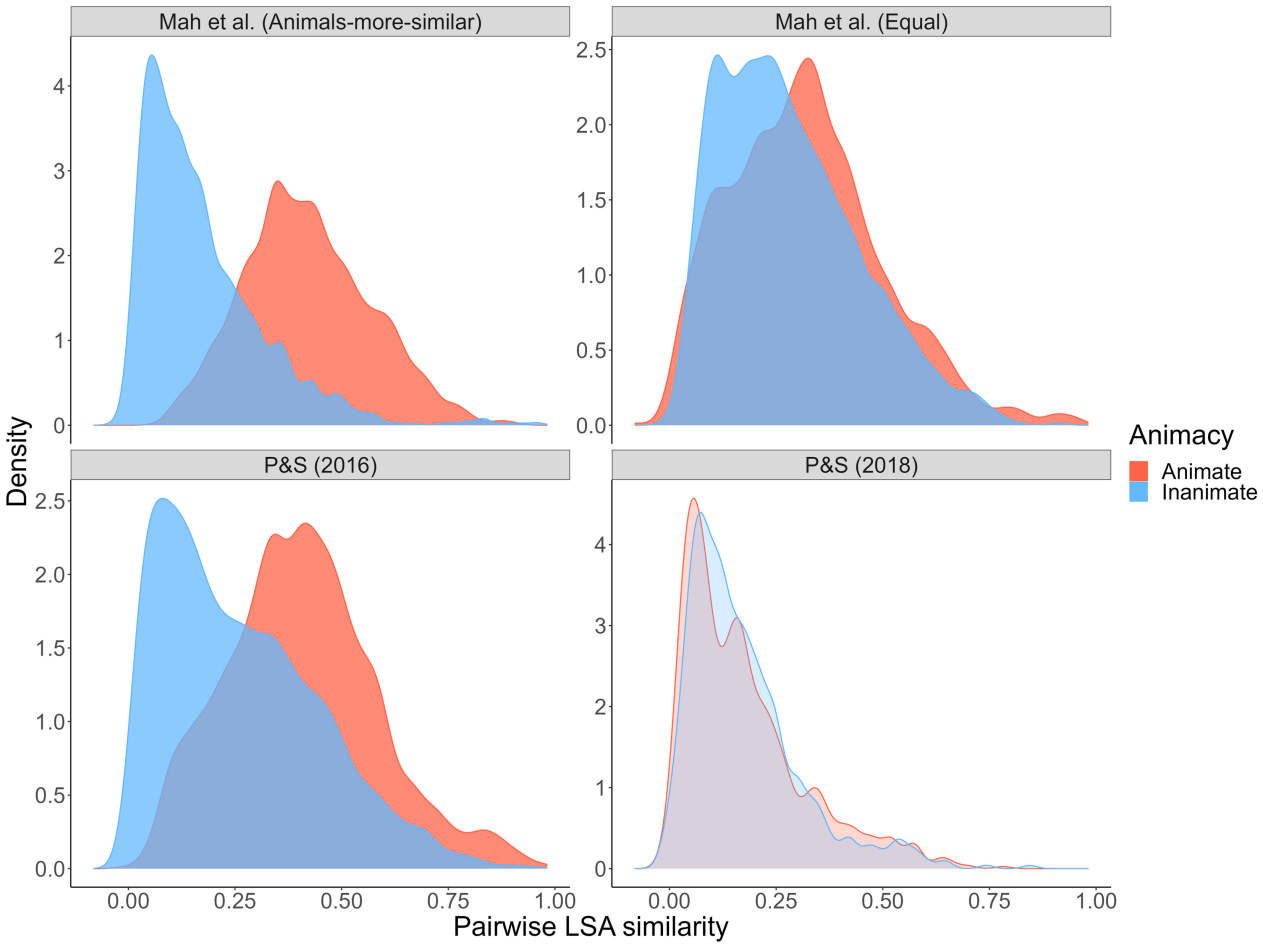
Simulated semantic similarity distributions: Current experiment and Popp and Serra (2016, 2018). Distributions represent pairwise Latent Semantic Analysis (LSA) similarity values for 10,000 randomly generated animal/object pairs from each wordset. High-opacity denotes experiments/samples in which a reverse animacy effect was observed.

However, we think it more likely that semantic similarity is not the primary mechanism behind the reverse animacy effect. Initially, it seemed that patterns in semantic similarity distributions coincided with the presence (or not) of a reverse animacy effect, but that is less clear with our results (see Figure 5).

**Figure 6:**
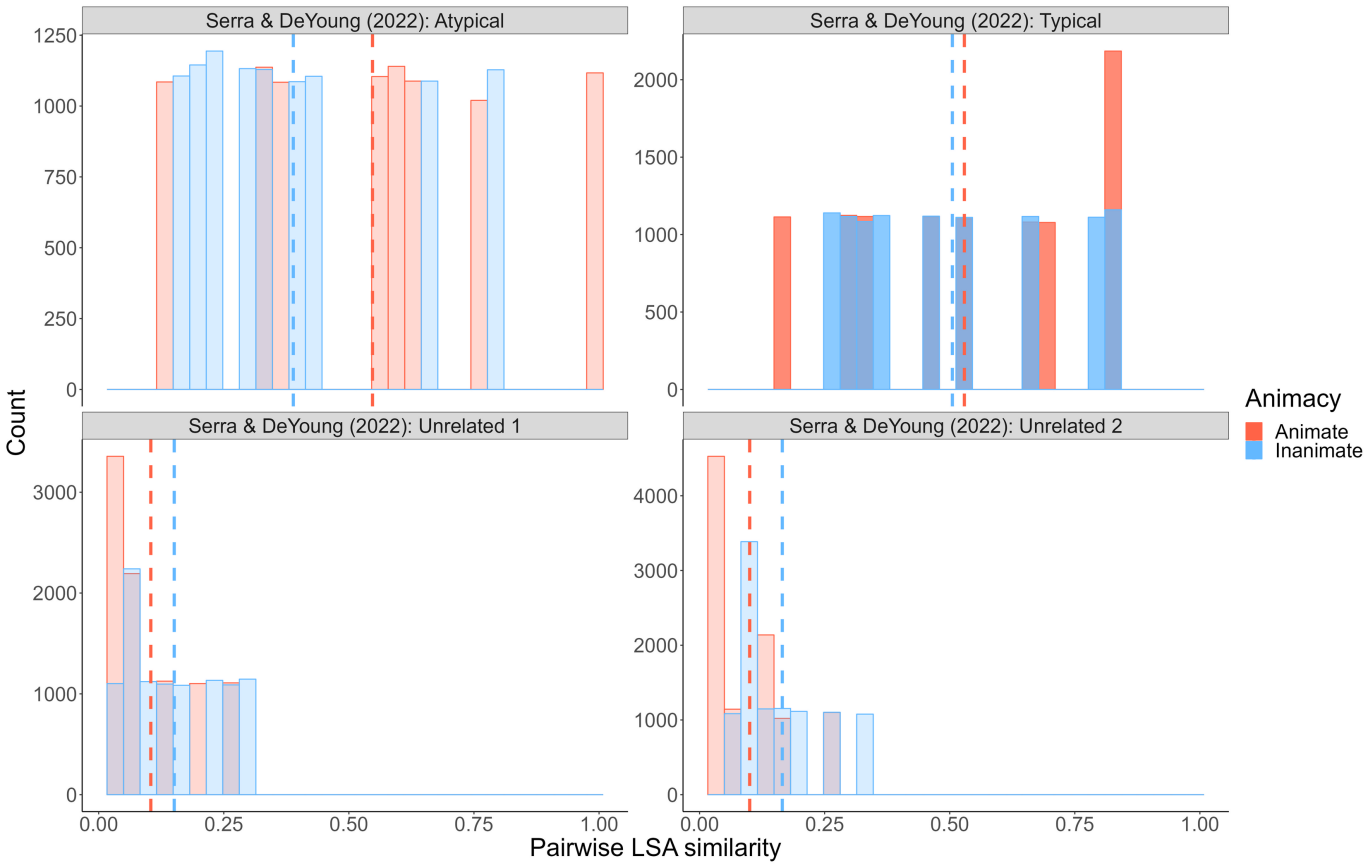
Simulated semantic similarity distributions: Serra and DeYoung (2022). Distributions represent pairwise similarity values for 10,000 randomly sampled animal/object pairs from each wordset. High-opacity denotes experiments/samples in which an animacy effect (animacy advantage) was observed.

Crucially, with high distributional overlap, we observed a reverse animacy effect but Popp and Serra (2018) did not (in fact, they observed a *reverse* reverse animacy effect – higher recall of animal than object targets). As per the Popp and Serra (2018) similarity distributions, it could be that similarity has to be equated *and/or* low overall. In a recent independent study published during our data collection, Serra and DeYoung (2022) also explored potential within-pair factors that might account for the reverse animacy effect. They used a smaller, fixed set of animal and object word pairs that were manipulated to involve either two typical exemplars from the same category (e.g., SALMON–TROUT; FORK–SPOON), one typical and one atypical exemplar from the same category (e.g., SALMON–SNAPPER; FORK–STOVE), or two unrelated exemplars (e.g., SALMON–RECEIVER; FORK–NAIL). With these pairs types respectively, they observed the standard cued recall reverse animacy effect (i.e., higher recall of inanimate than animate typical-typical pairs), a cued recall animacy effect (i.e., higher recall of animate than inanimate typical-atypical pairs), and no difference by animacy (with unrelated pairs). As Figure 6 shows, a *post hoc* analysis of their wordsets similarly failed to reveal a consistent relationship (at least a clear one that we can see) between LSA semantic similarity and the presence/absence of a reverse animacy effect:

On the basis of these results, one might speculate that (a) higher animal than object similarity produces an animacy effect, (b) equal-and-moderate similarity produces a reverse animacy effect, and (c) equal-and-low similarity produces no animacy difference. However, these patterns are not consistent when one considers all the wordsets we examined.^12^ In their study, Serra and DeYoung (2022) conducted additional exploratory analyses using word similarity ratings grounded in the LSA and GloVe theories and found that their constructed typicality categories did not map onto the within-pair similarity measures (and analyses of the within-pair similarity measures were generally inconclusive). As a result, they argue that paired-associate animacy and reverse animacy effects are likely not due to typicality or similarity *per se*, but some other aspect of memorability imperfectly related to these factors (and perhaps more related to typicality than similarity). Having now conducted a fulsome and high power experimental examination of the issue and nevertheless failing to reach a clear and forceful conclusion, we are inclined to agree with them.

## General discussion

In two experiments (and five samples), we investigated animacy and reverse animacy effects in free recall and cued recall. Overall, we replicated the basic pattern observed by Popp and Serra (2016) – better free recall for animals than objects, and better cued recall for objects than animals. Our results (and the results of other recent studies; e.g., Kazanas et al., 2020; Serra and DeYoung, 2022) provide further evidence for the robustness of these effects. Although the cued recall reverse animacy effect is replicable, results seem to vary as a function of materials.

In an effort to investigate underlying mechanisms and determine what specific materials-based factors might account for these inconsistencies, we considered one potential moderator – semantic similarity. Our key hypothesis was that equating animal and object within-category semantic similarity would eliminate or reduce the reverse animacy effect. In three out of the four samples that we collected, we either observed evidence against this hypothesis or failed to find evidence to support it. Based on these results (and examinations of other datasets), we argue that semantic similarity is not likely to be the mechanism behind the reverse animacy effect. Rather, the patterns of semantic similarity that we hypothesized to be causal (e.g., in Popp and Serra, 2016 and Popp and Serra, 2018) were perhaps confounded with the true underlying mechanism.

We are left to consider what other potential mediators or mechanisms might be at play here. As Serra and DeYoung (2022) suggest, it could be category typicality (or some other aspect affected by typicality). Another possibility is category size or specificity. In Experiment 1, and in the combined samples of Experiment 2, we coded and analyzed participants’ post-experiment self-reported perceptions about the words they studied. Data from Experiment 2 suggested that our similarity manipulation did not affect participant perceptions of the *granularity* or *specificity* of the animal and object categories (e.g., participants were no more likely to report a more general or cohesive “objects” category in the “equal” similarity condition vs. the “animals-more-similar” condition, see Supplementary online material Section 2H). The vast majority of participants in Experiment 2 indicated awareness of a superordinate “animals” category (82%), while less than half of participants indicated awareness of a superordinate “objects” category (47%), with minimal differences across conditions. Thus, it is possible that participants may have been influenced more by the general categories themselves than the specific semantic relationships for any particular list (see Higham and Brooks, 1997, for data on the issue even when participants do not express awareness of category structure in memory lists).

Awareness of the categories could have primed participants’ animal and object conceptual networks generally, irrespective of the similarity condition. So, although we intended for less spreading-activation-based interference (Collins and Loftus, 1975) for animals in our “equal” similarity condition (by creating a list of words less strongly linked with one another), it could be that participants became aware of the general animal category, resulting in widespread activation of animal concepts anyway. In other words, perhaps the influence of these general categories overshadowed activations of specific cues within the condition-varying semantic networks, leading to similar results in both conditions. As a final exploratory analysis in this vein, we compared the magnitude of the reverse animacy effect in two groups of Experiment 2 participants: those who indicated an awareness of superordinate “animal” and “object” categories, and those who indicated only an awareness of a superordinate “animals” category. The reverse animacy effect did not differ as a function of category awareness (see Supplementary online material Section 2Hd), but again, the vast majority of participants in our samples indicated awareness of a general animals category (i.e., we did not have enough unaware participants for a 3rd comparison group). We suggest that a “lack of awareness of a general animals category” may be more important than “awareness of a general objects category” for modulation of reverse animacy effects.

Similar effects (differential impacts of a manipulation on item versus associative memory tasks) have been observed in other paradigms. For example, Madan et al. (2012) and Bisby and Burgess (2013) found better item memory (free recall) but worse associative memory (cued recall in the former, cued recall/context memory in the latter) of emotionally arousing negative words/pairs than neutral words/pairs. Zimmerman and Kelley (2010) replicated this general pattern and also found substantial overconfidence in judgments of learning for negative relative to neutral pairs. These results suggest two possibilities – that the individually arousing words in studied pairs drew attention away from the association, or that subjects may have been overconfidence in their ability to remember emotionally arousing pairs (Madan et al., 2012). It is possible that these mechanisms might extend to animacy and reverse animacy effects.

Many studies have tested and ostensibly ruled out arousal (and proximate mechanisms such as attention) as an explanation for animacy effects (e.g., Meinhardt et al., 2018; Popp and Serra, 2018; Leding, 2019; Rawlinson and Kelley, 2021). However, metacognitive explanations for animacy and reverse animacy effects have received less attention. DeYoung and Serra (2021) did find higher judgments of learning for animate than inanimate pairs, but did not replicate the reverse animacy effect. Future studies replicating the reverse animacy effect while measuring participant judgments of learning are needed to determine the plausibility of metacognitive explanations – namely, that participants are overconfident in their ability to memorize and recall animate pairs and thus exert less effort studying them.

Like semantic similarity, specific animate and inanimate word categories vary across experiments. In our experiments and Popp and Serra (2016), the cued recall reverse animacy effect was observed (although this was not the case in DeYoung and Serra, 2021). The same wordset (consisted *only* of animals and objects) was used in these three studies. In Kazanas et al. (2020), reverse animacy effects were observed in three paired-associates tasks. Their wordset consisted of animate animals and inanimate clothing, fruits, and weapons. In Popp and Serra (2018), where a reverse animacy effect was not observed, the wordset consisted of a mix of animate humans and animals, and inanimate natural and manmade objects (DeYoung and Serra, 2021). In Serra and DeYoung (2022), the reverse animacy effect fluctuated based on the relative typicality of cues and targets. The wordset in their study consisted of a mix of animate humans described in terms of roles (e.g., doctor, quarterback) and animals versus exclusively manmade inanimate objects. Although these experiments were conducted in different samples (and in some cases using different tasks), it is suggestive that the reverse animacy effect was most consistently observed when the animate category consisted only of nonhuman animals. In line with our category size/specificity explanation, it could be that the use of more ambiguous categories (e.g., animate vs. animals) allows within-pair characteristics (like typicality; DeYoung and Serra, 2021) to modulate cued recall animacy effects. But when the general category is highly salient, it could be that category-specific effects dominate the influence of pair-level variables. We do not claim to answer these questions here, but it seems worthwhile to examine and compare animate and inanimate categories of varying levels of specificity. This idea of category specificity has been explicitly tested in only two studies. In one, VanArsdall et al. (2017) found a reverse animacy effect with constrained categories (i.e., four-footed animals, furniture), but they used a different task: English-Swahili word pairs in an English-speaking sample. In another, Gelin et al. (2017) controlled for category size and cohesion, but only tested participants on free recall. Perhaps the field needs a larger multi-lab replication effort to examine the benefit/cost of animacy in free/cued recall to settle the issue once and for all?

## Limitations

As mentioned previously, one possible limitation is that our Experiment 2 manipulation of within versus between category similarity could have been more forceful. Our “equal” similarity condition was noticeably different from the “animals-more-similar” condition in terms of semantic similarity distributions, but within-pair similarity was still slightly higher for animals relative to objects. A re-analysis of our wordset using an alternate similarity measure (GloVe; Pennington et al., 2014) revealed an even larger discrepancy between similarity distributions in favor of animals. Although our manipulation aimed to control for semantic similarity by equating average listwise and pairwise values across animacy categories, an “ideal” manipulation of category semantic similarity might have been a wordset in which within-pair similarity was higher for objects than animals. Over the course of many 1,000s of simulated wordsets of common animal and object words, we did not obtain a single wordset in which this was the case. Perhaps this speaks to the general categories themselves – the animal category might be inherently more cohesive and constrained than objects, restricting the degree to which manipulations can affect category-related outcomes. Such an explanation is consistent with the *overlapping semantic features* hypothesis (McRae et al., 1997). That is, animals may naturally share more overlapping features than is the case for objects. Popp and Serra (2018) and Serra and DeYoung (2022) were able to manipulate the reverse animacy effect using more general animate and inanimate categories. It might be that in addition to reducing the salience of the overall categories (as we have suggested above), their wordsets allowed for control of effects via a reduction of semantic feature overlap. If so, our hypothesis that there may be uninteresting but important psycholinguistic factors driving the reversed animacy effect in cued recall might be revived. However, the story is more complex than semantic similarity models like LSA and GLoVe currently capture.

### Constraints on generality

The remaining limitations relate to potential constraints on the generalizability of our findings (Simons et al., 2017). First, our observations and conclusions are specific to memory performance on the particular (quite artificial) free and cued recall tasks used in our experiments (i.e., words with certain characteristics presented singly or in random word-pairs for one at a time in random order with instructions to remember them for a subsequent test, followed by a brief retention interval and then tests of the sorts we have reported). Animacy effects in memory are often couched in real-world, evolutionarily relevant contexts (e.g., remembering predators or useful objects), so caution is advised in generalizing from these artificial tasks to more realistic remembering. Some experiments have examined the effects we report on here in more ecologically valid contexts (e.g., using survival-processing paradigms; Kazanas et al., 2020; or more complex/realistic stimuli like images; Bonin et al., 2014). Further experiments specifically examining the reverse animacy effect in more realistic contexts would help determine the generalizability of the effect, and shed light on the degree to which the effect is specific to word stimuli or word-related characteristics.

Our experiments also relied on two relatively constrained wordsets. As we have discussed, the wordsets used likely impact more than the generalizability of findings–rather, wordset characteristics likely relate to substantive mechanisms underlying the animacy and reverse animacy effects. Still, it is possible that even with another set of similar animals and objects, we might have observed different results. Although we considered and attempted to control for various other word characteristics related to memory (e.g., word frequency, age of acquisition, context diversity, imageability; Madan et al., 2010; Madan, 2021), it is impossible to perfectly match stimulus sets on all these categories (e.g., Clark, 1973). Even if it was possible, the extreme set of constraints on word selection would probably generate a list of words that resembles the natural category in some unusual and odd fashion that would render distinctiveness a going factor in understanding people’s memorial performance and study strategies. Although unlikely, it is possible that a wordset differing on one or more of these characteristics (e.g., lower overall word frequency) could have led to different patterns of results.

Finally, our experiments tested English-speaking participants sourced from undergraduate and online populations, with English concrete nouns carrying various psycholinguistic characteristics. Although the animacy effect on free recall has been replicated with multilingual stimulus sets (e.g., French, German, Chinese, Portuguese; Mieth et al., 2019), to our knowledge the reverse animacy effect has only been examined in English samples, with English stimuli. Our point here is not to claim that we have a theory that predicts different patterns of animacy and reverse animacy effects as a function of varying word frequency, context diversity, participant samples, languages and so forth. Would that we did. We are merely acknowledging potential constraints on the generality of our findings (Simons et al., 2017).

## Conclusion

The results of our experiments (and other experiments from Serra and colleagues) suggest that the reverse animacy effect in cued recall is a robust, replicable effect. Moreover, it does not appear that semantic similarity explains the effect. That is not to say that the effect cannot be ascribed to non-animacy pair-level factors. In fact, recent work from Serra and DeYoung (2022) suggests that within-pair factors such as typicality can explain the reverse animacy effect. However, like Serra and DeYoung, we do not think that these results rule out “adaptive memory” explanations for animacy and reverse animacy effects. Pair-level factors such as typicality or category-level factors such as category specificity might explain these effects, but could in turn be related to evolutionary factors.

Although we have not identified the specific mechanisms underlying the reverse animacy effect, our experiments bring the field closer. We provide evidence against an initially attractive candidate–word similarity–consistent with Serra and DeYoung’s suggestion of some mechanism related to typicality. Additionally, our exploratory analyses of category awareness and an informal review of wordsets in reverse animacy experiments point to a potential influence of the size, specificity, or granularity of the animate and inanimate categories used. We have also added to a growing body of openly available free and cued recall animacy data (e.g., see https://osf.io/7cx2r/) that we hope will be of use to other researchers examining these effects (e.g., allowing analyses of different word/category characteristics). Finally, we have helped to establish the replicability of the reverse animacy effect, but it remains unclear whether and how the effect generalizes to more realistic memory tasks.

## Data availability statement

The datasets presented in this study can be found in online repositories. The names of the repository/repositories and accession number(s) can be found at: https://osf.io/hc2af/.

## Ethics statement

The studies involving human participants were reviewed and approved by University of Victoria Human Research Ethics. Written informed consent for participation was not required for this study in accordance with the national legislation and the institutional requirements.

## Author contributions

EM, AC, NT, KG, and DL planned and conducted Experiment 1 and drafted the original writeup. EM, KG, RJ, and DL planned Experiment 2, and EM, KG, and DL conducted Experiment 2 (as part of KG’s Honours Thesis), and EM and KG drafted the writeup for Experiment 2. EM drafted the final version of the combined manuscript, and all authors revised this version before final submission.

## Funding

This work was supported by an NSERC Discovery Grant (#RGPIN-2016-03944) awarded to DL. Funds from this grant were used to pay online (Prolific.co) participants.

## Conflict of interest

The authors declare that the research was conducted in the absence of any commercial or financial relationships that could be construed as a potential conflict of interest.

## Publisher’s note

All claims expressed in this article are solely those of the authors and do not necessarily represent those of their affiliated organizations, or those of the publisher, the editors and the reviewers. Any product that may be evaluated in this article, or claim that may be made by its manufacturer, is not guaranteed or endorsed by the publisher.

## Footnotes

^1^We created a preregistration prior to data analysis, but due to technical issues this registration was not archived and we had to upload a copy of the registration after we had conducted our analyses. The new copy of the preregistration was identical to the pre-analysis version.

^2^Two of these participants did not correctly recall at least one word from both free recall lists (*N* = 2), and one participant reported experiencing a "major distraction that affected your ability to pay attention to the experiment." There were no missing/incomplete data in our final sample.

^3^In our preregistered plan, we initially intended that the ratio of animal:object words in each list be restricted to 7:8 and 8: 7, and mistakenly assumed that the program (and original experiment) worked this way. However, our replication (and the original experiment) included variable ratios as extreme as 4: 11. Along with testing additional hypotheses, we addressed this in three of the samples collected for Experiment 2.

^4^We did not plan or preregister any analyses of these demographics variables, and included them only to provide a picture of our sample and to allow other researchers to examine these variables.

^5^These Bayes Factors captured ratios of prior-weighted marginal likelihoods for models with and without effects of interest. In other words, the ratio of how well one model explains the data relative to another.

^6^To account for apparent non-normality in proportion correct, we also conducted nonparametric Wilcoxon signed-rank tests. In all cases, the parametric and nonparametric analyses agreed (i.e., both were either significant or non-significant).

^7^*d_z_ =* Cohen’s *d* for within-subjects comparisons, with the 95% Confidence Interval on the effect size estimate in brackets.

^8^See Supplementary online material X for an exhaustive description of our word-selection process.

^9^We drafted but did not preregister a plan for the first undergraduate sample because we discovered after data collection but before analysis that the program was not assigning equal numbers of animals/objects to each study list as we had intended (see footnote 2). We include the experiment here because both Popp and Serra (2016) and our replication also had unequal category-list assignment, and our core hypotheses described above did not change after the discovery of the error. The draft version of the preregistration can be viewed here: https://docs.google.com/document/d/18oKBq7jxl-4Pz7zZ6iQBIVeflmaU83QU7oXk0UzGi1s/edit?usp=sharing. For consistency across experiments, we analyzed all data according to the preregistration for the latter three samples.

^10^Although we preregistered a general experiment plan, we did not initially plan to collect four samples. Rather, we conducted the experiments one at a time, making decisions as to the details as we went along.

^11^Using the common crawl space with 840B tokens, 2.2 M vocabulary, cased, and 300d vectors.

^12^Results were similar when analyzing GloVe similarity, see Supplementary online material Section 2D. See Supplementary online material Section 2G for descriptive statistics for the similarity metrics and the presence of reverse animacy effects.
