# Supplementary material for: A direct replication and extension of Popp and Serra (2016, experiment 1): better free recall and worse cued recall of animal names than object names, accounting for semantic similarity
Source: Front Psychol. 2023 May 18;14:1146200. doi: 10.3389/fpsyg.2023.1146200 (PMC10232972; doi:10.3389/fpsyg.2023.1146200)
Supplement: Supplementary file 1 [file Data_Sheet_1.docx]

# Supplementary Online Material

**1. Experiment 1**

## Bayesian analyses

Bayesian analyses have several advantages over standard NHST, including the ability to explicitly model and provide evidence for both the null and alternative hypotheses (see Wagenmakers et al., 2018, for a review, and the 2018 *Psychonomic Bulletin & Review* special issue (Vol. 25, Issue 1) on Bayesian statistics for comprehensive methodological coverage). We conducted Bayesian linear models (McElreath, 2019) and follow-up contrasts analogous to our NHST analyses. These analyses provided evidence ratios for models, hypotheses, and effects in the form of Bayes Factors (BFs). BFs represent the relative evidence for one model/hypothesis/effect over another—for example, a BF = 50 suggests that one model is 50 times more likely than the other, given the data.

1. **Calculation and evaluation of Bayes Factors (BFs)**

The BFs reported for evaluation of main and interaction effects refer to model-comparison BFs (i.e., ratios of prior-weighted marginal likelihoods^^[[1]](#footnote-1)^^). In these cases, BFs represent the relative evidence favoring a model with an effect to a model without that effect. BFs for individual effects (i.e., pairwise comparisons or *t* tests) were calculated using the Savage-Dickey density ratio method (Wagenmakers et al., 2010, see also <https://vuorre.netlify.com/post/2017/03/21/bayes-factors-with-brms/>). This method provides the ratio of the probability of a specified effect size (e.g., 0) under the prior distribution (before we have seen the data) to the probability under the posterior distribution (after we have seen the data). Essentially, a Savage-Dickey BF represents the degree by which we should update our pre-data beliefs for or against an effect, after seeing the data.

Though interpretation of BF cutoffs is somewhat subjective, we adopt the following standards proposed by Jeffries (1961, outlined in Wagenmakers et al., 2011): 1 ≤ BF < 3 = Anecdotal evidence; 3 ≤ BF < 10 = Substantial evidence; 10 ≤ BF < 30 = Strong evidence; 30 ≤ BF < 100 = Very strong evidence; BF ≥ 100 = Extreme evidence. For the current analyses, we treat BFs ≥ 3 as non-trivial evidence for or against an effect/model difference. To calculate BFs, we obtained posterior distributions of effects via Hamiltonian MCMC sampling (see McElreath, 2019 for an overview and <https://elevanth.org/blog/2017/11/28/build-a-better-markov-chain/> for an interaction demonstration). Models were run using 4 chains of 11,000 iterations (1,000 warmup) each.

1. **Results**
   1. **Popp and Serra scoring**

We first conducted analyses using proportion of targets accurately recalled as calculated in the original experiment (manual scoring for free recall responses, automatic 3-first-letters scoring for cued recall responses.

1. **Priors**

We chose priors that were based on the results of the original experiment, but allowed for substantial variation in the effects of animacy and memory type. See SOM A3 (Figure S1) for a visual representation of the chosen priors.

1. **Model comparison results**

To evaluate evidence for/against main/interaction effects, we compared mixed-effects Bayesian linear models (McElreath, 2019) of increasing complexity via hierarchical forward entry. Specifically, we compared an animacy main-effect model to an intercept-only model, a model with animacy and memory type main effects to the animacy main-effect model, and a model with an animacy ✕ memory type interaction to the dual main effects model^^[[2]](#footnote-2)^^. These model comparisons revealed anecdotal evidence *against* an animacy main effect (BF = 1.67), extreme evidence *for* a memory type main effect (BF > 100), and extreme evidence *for* an interaction (BF > 100). These results corroborate the NHST results, and additionally suggest that there may or may not be an overall animacy effect.

1. **Evaluation of individual effects**

We also calculated Savage-Dickey BFs for individual pairwise contrasts (Wagenmakers et al., 2010, see also <https://vuorre.netlify.com/post/2017/03/21/bayes-factors-with-brms/>). These BFs are commonly used to test specific hypotheses, and represent the ratio of the probability of a specified effect size (e.g., 0) under the prior distribution (before we have seen the data) to the probability under the posterior distribution (after we have seen the data). The Savage-Dickey contrast BFs obtained in favor of an animacy effect for free recall and a reverse animacy effect for cued recall were both > 100; extreme evidence for the hypothesized effects (relative to null effects).

We also used Savage-Dickey BFs to evaluate the degree to which we should update our beliefs in the original effects. We observed BFs in the range of 1.5 - 3.5, suggesting that (a) our replication results should increase belief in the original effect sizes by a modest amount and (b) our replication results do not entail a radical shift in beliefs from the original experiment (further evidence that the original results are robust). Using the Savage-Dickey method, we calculated BFs for the crucial differences, namely, the animacy effect for each memory type. For this analysis, we calculated the relative likelihood of the following effect sizes in the posterior relative to the prior distribution:

1. Cued recall Animacy effect > 0
2. Free recall Animacy effect > 0
3. Cued recall Animacy effect = .1074 (Original experiment result)
4. Free recall Animacy effect = .1463 (Original experiment result)

Analyses 1 and 2 allowed us to evaluate the relative likelihood of null effects given the data, and Analyses 3 and 4 allowed us to get a sense of how we should update our beliefs regarding the original effect sizes. In addition to computing these four BFs using our pre-registered priors, we also conducted exploratory analyses with tighter priors using the exact effect sizes obtained in the original experiment (see SOM A3 Figure S2). This exploratory version provides more insight into Analyses 3 and 4 (i.e., a direct comparison of plausible effect sizes in the original and replication experiments). The results of both versions of these analyses are displayed below in Table S1.

**Table S1**

*Savage-Dickey Bayes factors for individual effects: Popp and Serra scoring*

| Effect | BF  (Pre-registered priors) | BF  (Replication priors) |
| --- | --- | --- |
| Cued recall Animacy effect ≠ 0 | > 100 | > 100 |
| Free recall Animacy effect ≠ 0 | > 100 | 20 |
| Cued recall Animacy effect = .1074 | 2.78 | 1.55 |
| Free recall Animacy effect = .1463 | 1.86 | 3.45 |

Thus, our replication provided strong-to-extreme evidence against null effects. Interpreting the relative likelihood of the original effects, given our replication, our results suggest that we should increase our belief in the original effects by a modest amount (i.e., a factor of ~1.5 - 3).

- 1. **Manual scoring**

We also conducted analyses using proportion of targets accurately recalled as calculated with our scoring method, which was automatic for responses that were verbatim matches for recall targets and manual for responses that were not verbatim matches for recall targets.

1. **Priors**

Priors for this analysis were the same as the previous analysis (refer to Supplementary Material A3 Figures S1 & S2).

1. **Model comparison results**

We adopted the same model comparison strategy as in the previous analysis. These model comparisons revealed anecdotal evidence *against* an animacy main effect (BF = 1.92), extreme evidence *for* a memory type main effect (BF > 100), and extreme evidence *for* an interaction (BF > 100). These results corroborate the NHST results and are nearly identical to our results when using the original Popp and Serra scoring method.

1. **Evaluation of individual effects**

As with the Popp and Serra scoring analysis, we calculated Savage-Dickey BFs for the critical effects of interest, using both pre-registered and exploratory replication priors. The results of both versions of these analyses are displayed below in Table S2.

**Table S2**

*Savage-Dickey Bayes factors for individual effects: Manual scoring*

| Effect | BF (Pre-registered priors) | BF (Replication priors) |
| --- | --- | --- |
| Cued recall Animacy effect ≠ 0 | > 100 | > 100 |
| Free recall Animacy effect ≠ 0 | > 100 | 12.5 |
| Cued recall Animacy effect = .1074 | 2.94 | 1.71 |
| Free recall Animacy effect = .1463 | 2.10 | 3.45 |

Again, these results are nearly identical to the results of our analyses using the original Popp and Serra scoring method.

1. **Priors for Bayesian analyses**
   1. **Preregistered priors**

**Figure S1**

*Prior predictive simulation: Density plot of 4,000 simulated draws from the prior distribution (pre-registered priors)*

**
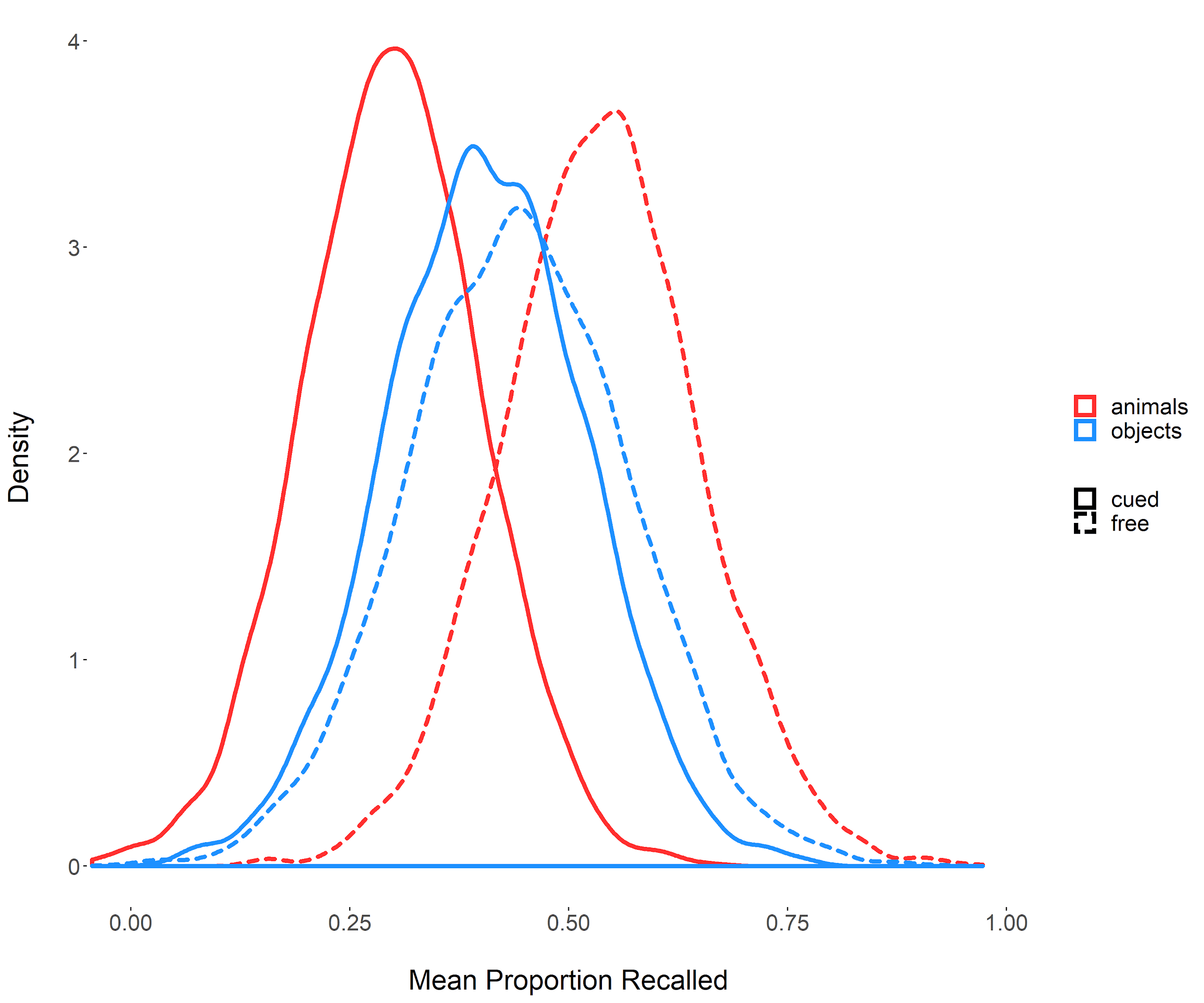
**

Note that the above distributions include sampling variability as a result of simulation from the full model, taking into account *all* specified priors. Thus, this figure (and all other like it) is meant to provide a more intuitive understanding of the implications of our chosen priors. The exact formal specification of the priors is as follows:

Proportion correctly recalled ~ Normal(*m_i,_ σ_i_*)

*m_i_* = 𝛼_participant_ + 𝛽_1_Animacy + 𝛽_2_Memory Type + 𝛽_3_Animacy ✕ Memory Type

𝛼_participant_ ~ Normal(*m*_participant_,  *σ*_participant_)

*m*_participant_ ~ Normal(.30, .10)

*σ*_participant_ ~ Normal(.1248, .02)

𝛽_1_ ~ Normal(.1074, .05)

𝛽_2_ ~ Normal(.2389, .05)

𝛽_3_ ~ Normal(-.20, .025)

*σ_i_* ~ Normal(.1481, .0105)

- 1. **Exploratory replication priors.**

**Figure S2**

*Prior predictive simulation: Density plot of 4,000 simulated draws from the prior distribution (exploratory replication priors)*

**
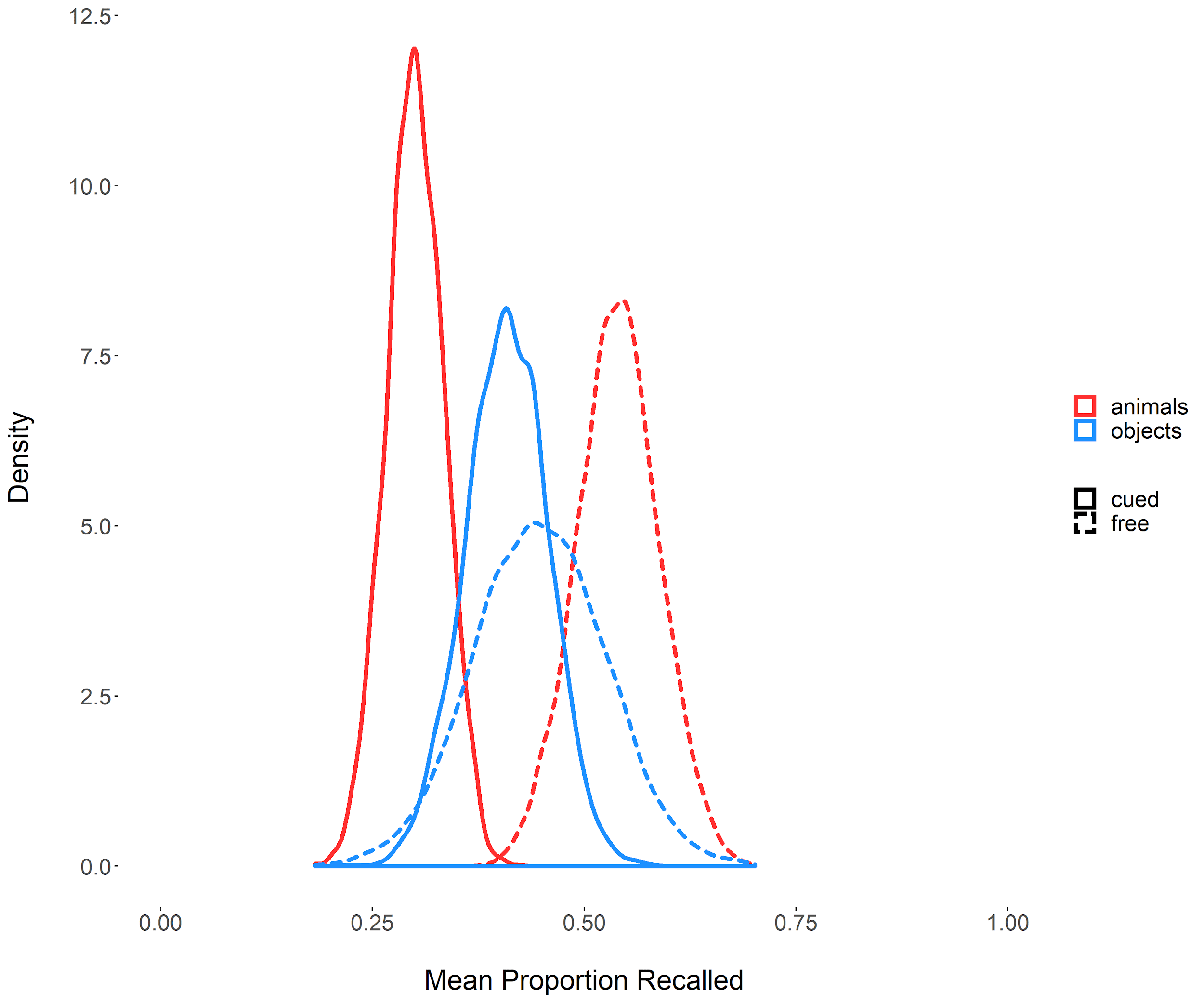
**

Note that the above distributions include sampling variability as a result of simulation from the full model, taking into account *all* specified priors. The exact formal specification of the priors is as follows:

Proportion correctly recalled ~ Normal(*m_i,_ σ_i_*)

*m_i_* = 𝛼_participant_ + 𝛽_1_Animacy + 𝛽_2_Memory Type + 𝛽_3_Animacy ✕ Memory Type

𝛼_participant_ ~ Normal(*m*_participant_,  *σ*_participant_)

*m*_participant_ ~ Normal(.30, .0326)

*σ*_participant_ ~ Normal(.1248, .0214)

𝛽_1_ ~ Normal(.1074, .0348)

𝛽_2_ ~ Normal(.2389, .0349)

𝛽_3_ ~ Normal(-.20, .0492)

*σ_i_* ~ Normal(.1481, .0105)

- 1. **Exploratory Priors for the perceived ease of recalling each word type by memory test type**

**Figure S3.**

*Prior predictive simulation: Density plot of 4,000 simulated draws from the prior distribution (exploratory priors)*

*
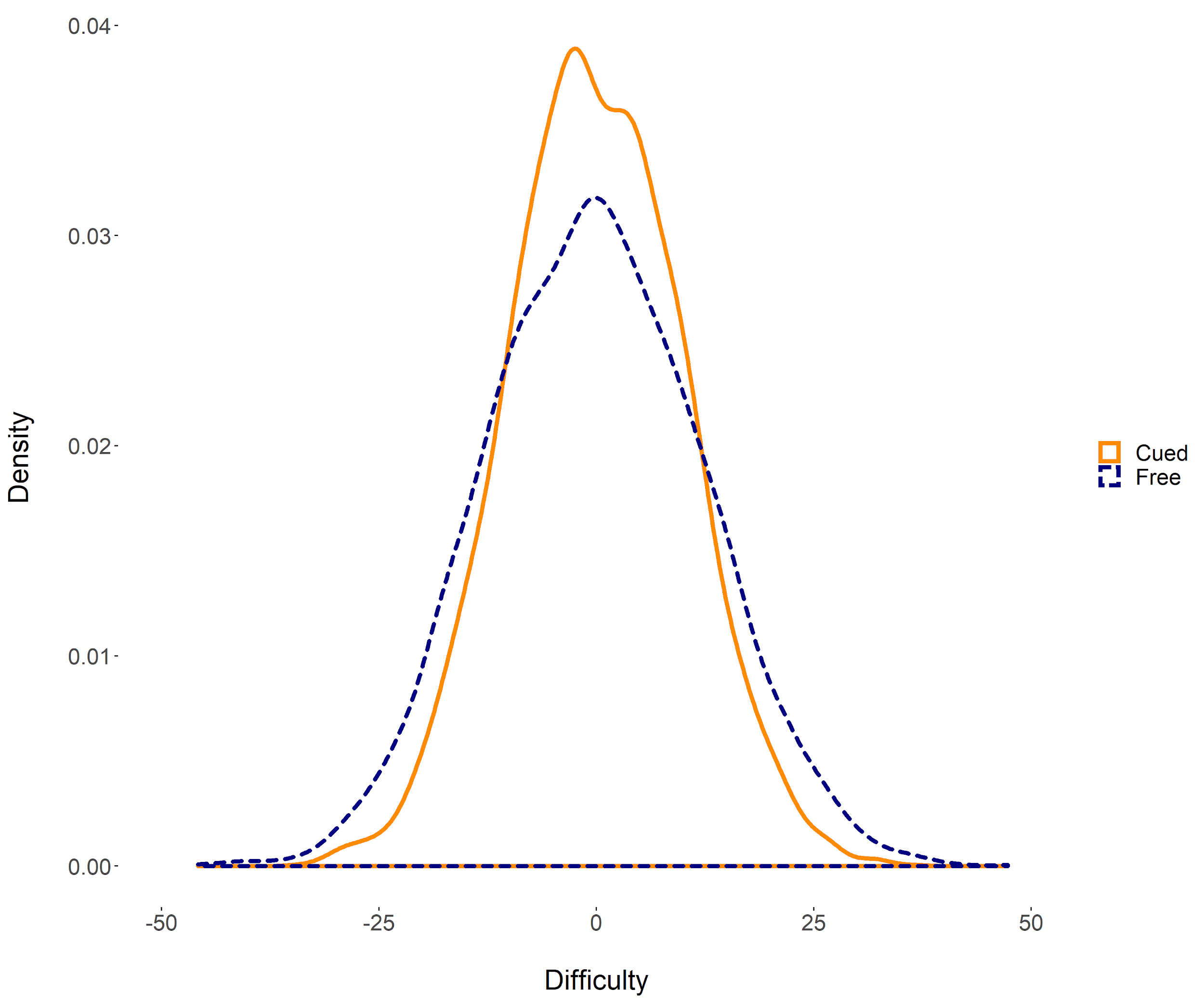
*

Note that the above distributions include sampling variability as a result of simulation from the full model, taking into account *all* specified priors. The exact formal specification of the priors is as follows:

Difficulty ~ Normal(*m_i,_ σ_i_*)

*m_i_* = 𝛼_participant_ + 𝛽_1_Memory Type

𝛼_participant_ ~ Normal(*m*_participant_,  *σ*_participant_)

*m*_participant_ ~ Normal(0, 10)

*σ*_participant_ ~ Exponential(1)

𝛽_1_ ~ Normal(0, 7.5)

*σ_i_* ~ Normal(0, 10)

- 1. **Exploratory Priors for the relationship between relative recall performance and relative recall difficulty**

**Figure S4A.**

*Prior predictive simulation: 100 regression line draws from the prior distribution*

**
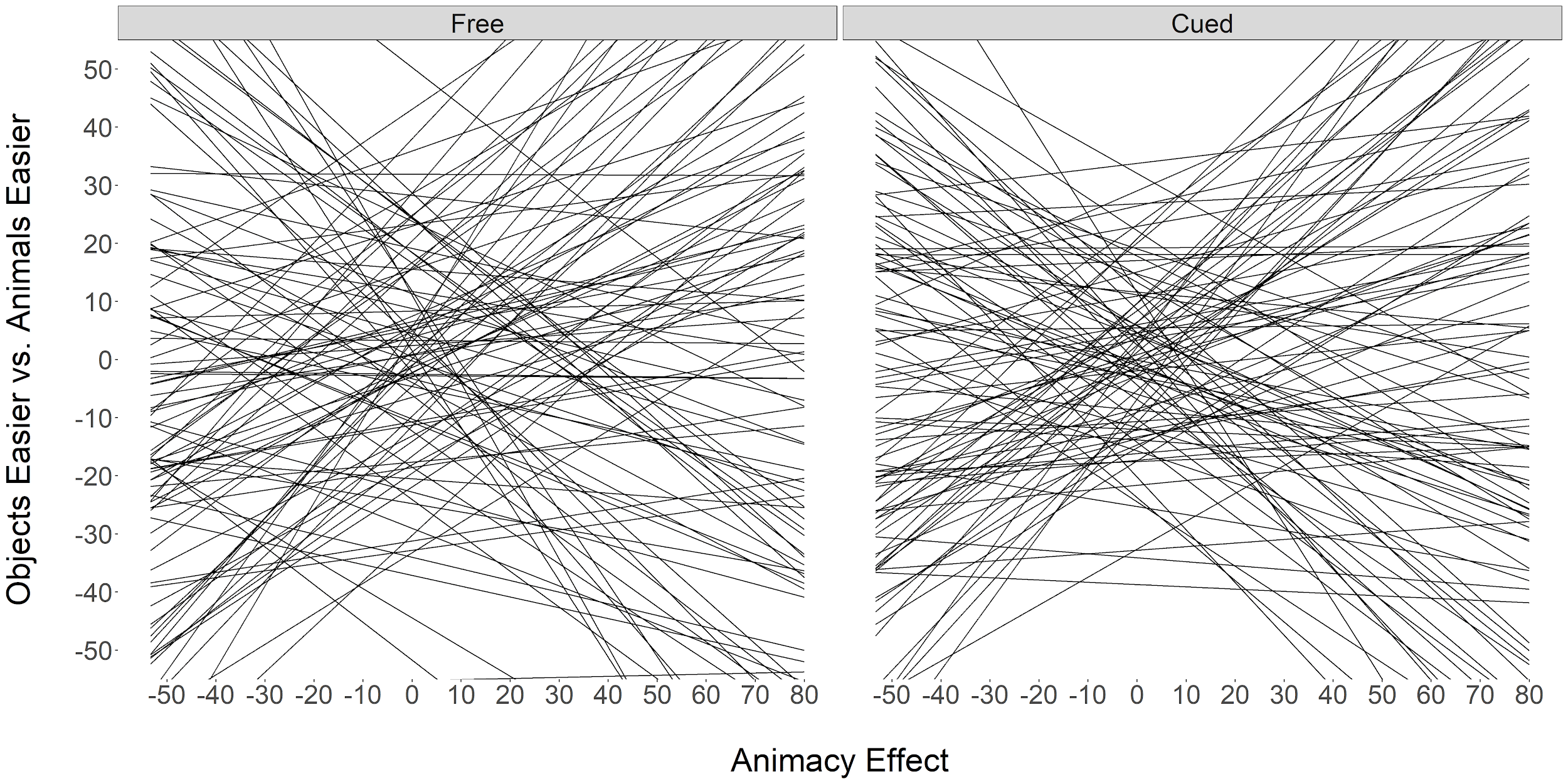
**

**Figure S4B.**

*Prior predictive simulation of intercepts by memory type*

**
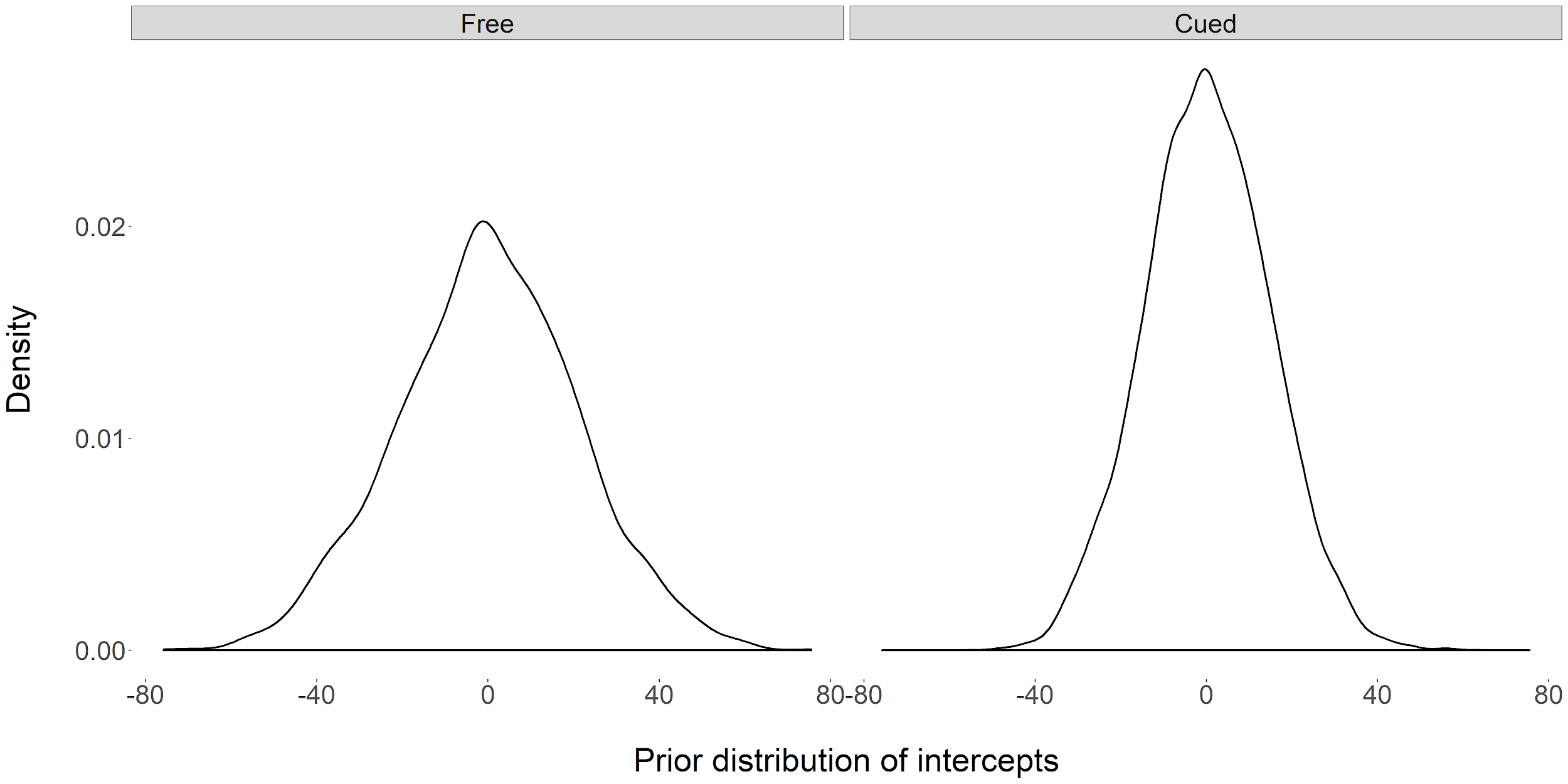
**

**Figure S4C.**

*Prior predictive simulation of slopes by memory type*

**
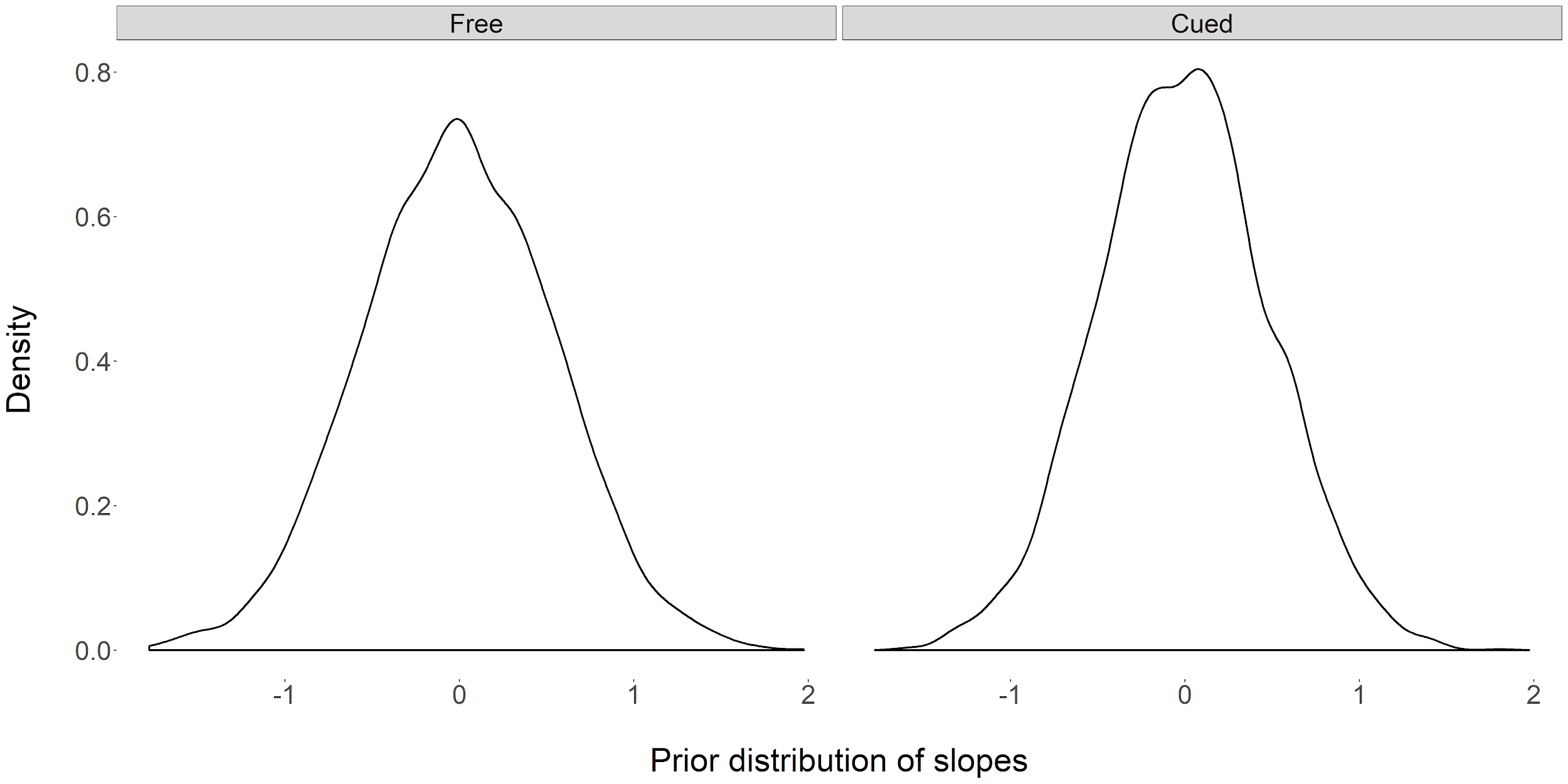
**

Note that the above distributions include sampling variability as a result of simulation from the full model, taking into account *all* specified priors. The exact formal specification of the priors is as follows:

Difficulty ~ Normal(*m_i,_ σ_i_*)

*m_i_* = 𝛼_participant_ + 𝛽_1_Animacy + 𝛽_2_Memory Type + 𝛽_3_Animacy ✕ Memory Type

𝛼_participant_ ~ Normal(*m*_participant_,  *σ*_participant_)

*m*_participant_ ~ Normal(0, 15)

*σ*_participant_ ~ Exponential(1)

𝛽_1_ ~ Normal(0, 0.5)

𝛽_2_ ~ Normal(0, 15)

𝛽_3_ ~ Normal(0, 0.25)

*σ_i_* ~ Normal(0, 5)

## Manual Scoring

Popp and Serra manually checked free recall responses that did not exactly match any word in the study list for cases in which subjects appeared to have misspelled a studied word (e.g., “lamma” when “llama” was on the study list) and counted those as correct recalls. For cued recall, in contrast, a response was scored as correct if and only if the first three letters matched those of the correct response (e.g., “lamma” would have been scored as incorrect if the target was “llama”). We conducted a separate analysis of proportion correctly recalled using the manual scoring method for both conditions. Proportion correctly recalled was again analyzed with a 2 (animacy: animals, objects) ✕ 2 (memory type: free recall, cued recall) within-subjects ANOVA. Consistent with both the findings of Popp and Serra and our initial results using the original scoring method, proportion correctly recalled was higher for free recall (*M* = .61, *SD* = .19) than cued recall (*M* = .51, *SD* = .28), *F*(1, 100) = 22.60, *p* < .001, η*_p_*^2^= .18. The main effect of animacy was not significant, *F*(1, 100) = 1.27, p = .26, η*_p_*^2^ = .01, and the interaction between animacy and memory type was significant, F(1, 100) = 37.80, p < .001, η*_p_*^2^ = .27. As before, we probed this interaction using paired-samples *t* tests (Bonferroni corrected). This revealed that, in consensus with our initial findings, free-recall proportion correctly recalled for animals (*M* = .64, *SD* = .17) was higher than for objects (*M* = .58, *SD* = .21), *t*(100) = 3.24, *p* = .002, *d*_z_ = .32 [.12, .52], and that cued-recall proportion correctly recalled was better for object pairs (*M* = .55, *SD* = .29) than for animal pairs (*M* = .47, *SD* = .25), *t*(100) = 4.52, *p* < .001, *d*_z_ = .45 [.24, .65]. These results indicate that, for our study, differences in scoring methods did not produce significant changes in the observed relationship between animacy and memory type.

## Effect of Test Order and Study/Test Block Order

A 2 (animacy: animal vs. object) ✕ 2 (test type: cued vs. free recall) ✕ 2 (block order: first study/test block vs. second study/test block) ✕ 2 (test order: cued recall first vs. free recall first) ANOVA was performed to investigate effects of block order on test performance. In addition to the original finding of a main effect of test type, *F*(1, 99) = 21.03, *p* < .001, η_p_^2^ = .18, and a test type by animacy interaction, *F*(1, 99) = 38.6, *p* < .001, η_p_^2^ = .28, we also found a significant interaction of test order and block order, *F*(1, 99) = 10.67, *p* = .001, η_p_^2^ = .10. This interaction was not statistically significant in the original Popp and Serra (2016) data, *F*(1, 34) = 1.57, *p* = .22, η_p_^2^ = .04. Because we had no a priori predictions about the effect of test order on block order effects, and this interaction did not involve the animacy effects of interest, we did not conduct follow up analyses.

1. **Reaction Time analyses**

We were also interested in identifying patterns between response type (i.e. correct responses, responses left blank (omission errors), and erroneous attempted responses (commission errors)) and response time in the cued-recall task. Response time was operationalized as the time between stimulus onset and the submission of a participant's response. Using the Popp and Serra (2016) data as a guide, we categorized response times beyond the range of 40 seconds as being anomalous (99.7% of Popp and Serra (2016) responses were made within 40 seconds). Indeed, of our 3030 total observations, only 8 were outside the response time range of 40 seconds (.03%). With this criterion for outliers in place, we began looking at patterns in response time by response type. In our sample of 3030 responses, 1491 were incorrect responses (747 commission errors, 744 omission errors), and 345 of those errors (206 commission errors, 139 omission errors) were made outside the response time range of 1-10 seconds (see, Figure S5).

**Figure S5**

*Response times by response type: Raincloud plots*


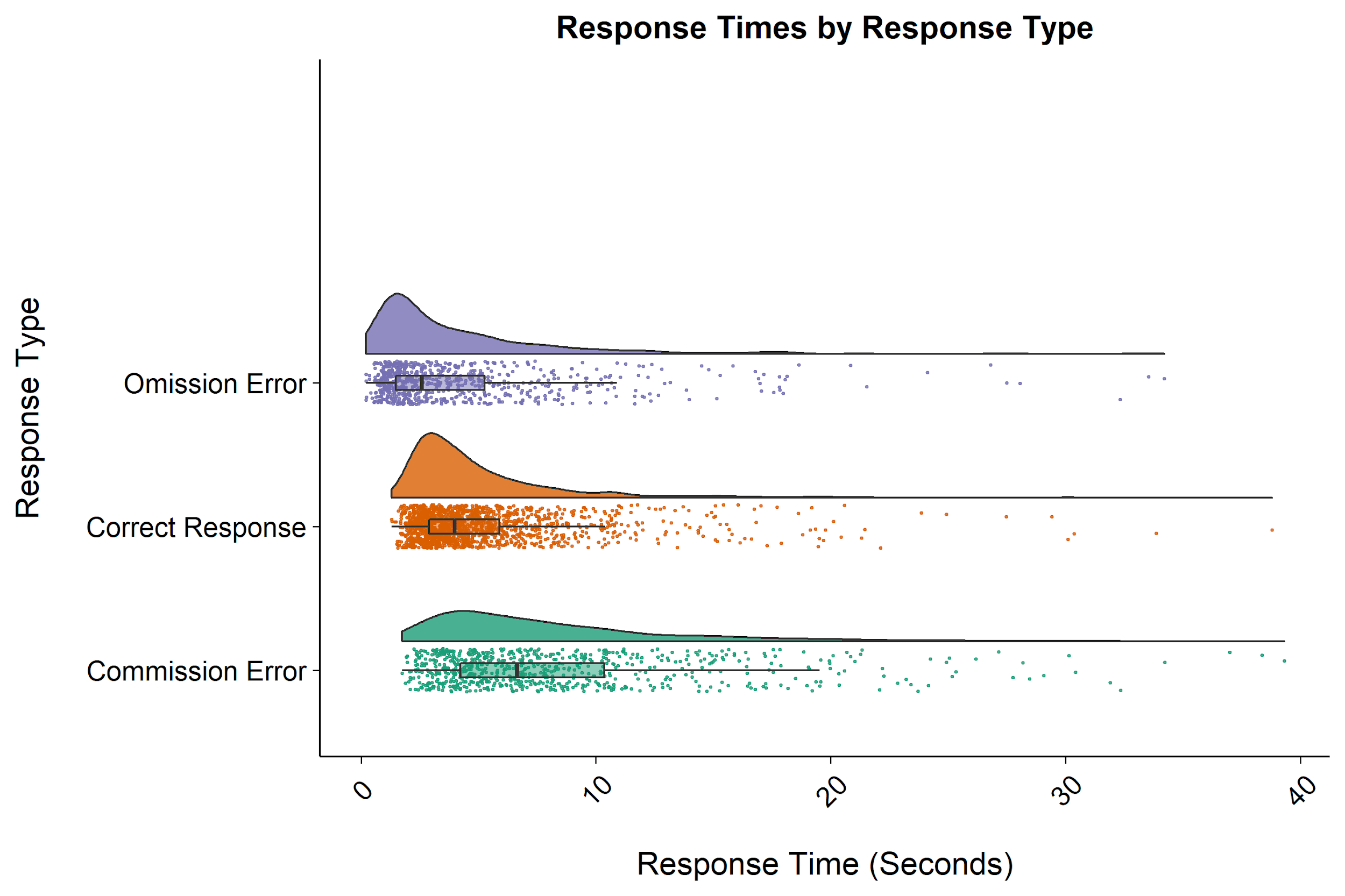


82% of all responses were made within 10 seconds of stimulus onset, which was slightly lower than the 92% of responses made within 10 seconds identified by Popp and Serra (2016). As shown in Figure S6, commission errors were found to be significantly slower than both correct responses and omission errors, even when excluding response times over 40 seconds.

**Figure S6**

*Response times by response type: Means and 95% CIs*


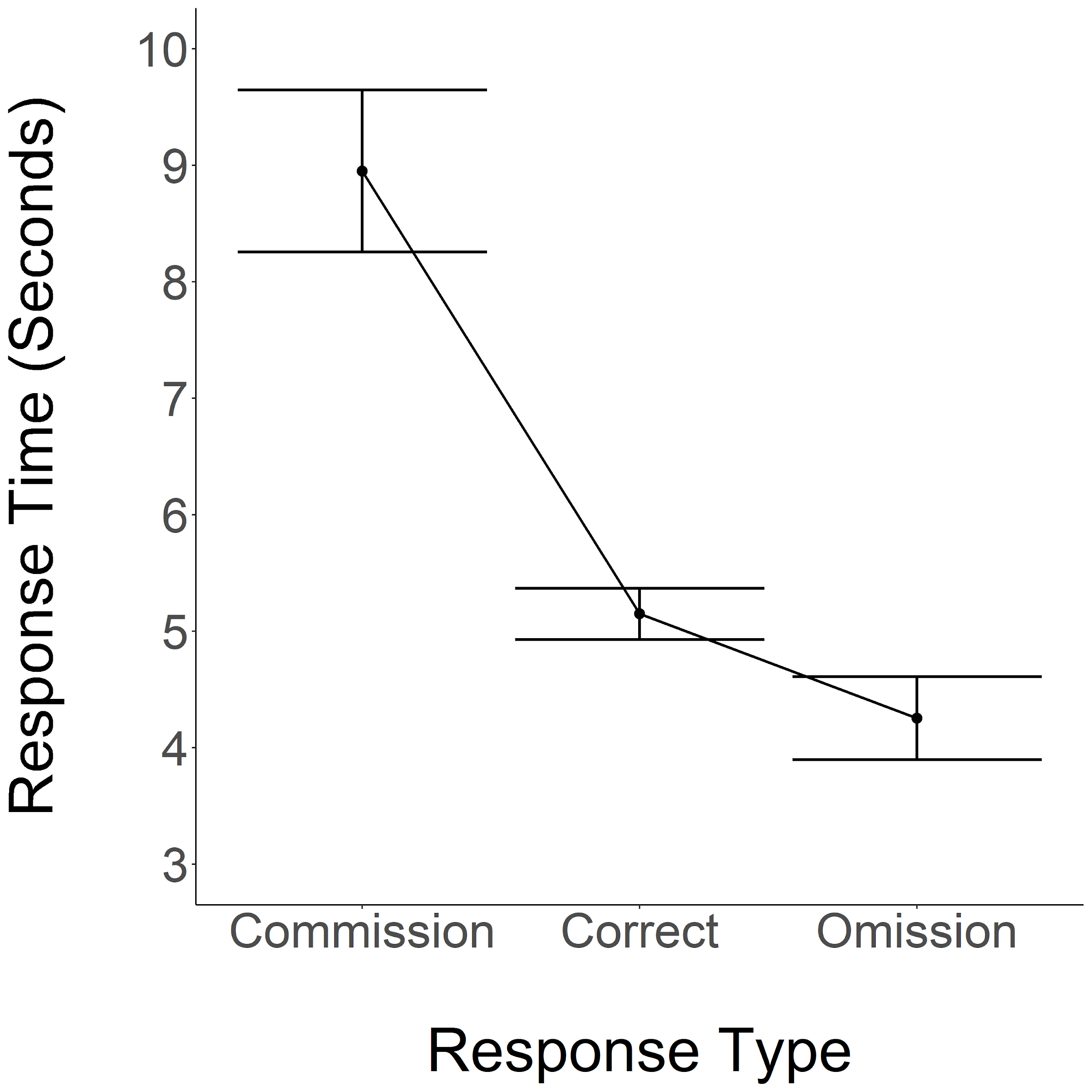


This pattern is unsurprising given that, for this short term memory task, without adopting advantageous mnemonic strategies the longer an individual perseverates on generating a correct response the more erroneous their recollection may be. Indeed, in our sample, over 82% of correct responses occurred within 7 seconds of stimulus onset. An interesting further investigation of this pattern would be to see whether the commission errors were typically composed of elements previously studied in any of the given lists, or if they were confabulations. Furthermore, it would also be valuable to see whether certain error types (i.e. commission and omission) were more likely with animate pairs or inanimate pairs. A final thought may be to look at mixed-animacy study pairs, where the paired-associates could be a combination of animate and inanimate stimuli, and then evaluate whether errors or correct responses are differentially affected by the animacy of the stimulus or the response word (e.g. is memory better when the studied stimulus word is animate, or when the response word is animate). Based on these findings of response latency, it may be appropriate to consider exclusion criteria based on response time. For example, in our sample we did register a few responses that were greater than 100 seconds. All of these cases were errors, and could be assumed to be distractions. Therefore, when evaluating participant-level data, response times could influence a researchers decision on whether to include or exclude participants. A participant showing consistently fast omission errors is perhaps unable to perform the test, or skipping a majority of the questions, whereas an individual with consistently long erroneous response times is perhaps sufficiently distracted or uninvested in the project. Thus, evaluating response times and their relationship with response type could be an important consideration when considering a priori exclusion criteria.

1. **Paired-associates interference: Within-study same-category commission errors**

Popp and Serra noted that subjects might report words that they recalled as guesses. Even a word presented on the study list might be generated as a guess (e.g., if it was not encoded at study). Moreover, Popp and Serra speculated that their animate names might be a “narrower,” more closely associated set than their inanimate names. That would promote guessing of studied animate names, relative to guessing of studied inanimate names. On the free recall test, such guesses would inflate performance of animate names despite being “lucky intrusions”. On the paired-associates recall test, in contrast, guessing would be less helpful, and might even interfere with correct report of animate names. For one thing, most studied words generated as paired-associate guesses would have been studied with a different cue word. For another, having a guessed word come to mind might interfere with recall of the target. Popp and Serra referred to this as paired-associate interference.

To explore the possibility that paired-associate interference accounted for the reverse animacy effect, Popp and Serra (2016) reported an exploratory analysis in which any incorrect animal/object word recalled in place of a correct animal/object target was counted as correct, irrespective of whether the incorrect word was studied or not. For example, if a participant studied the cue-target pair “glasses – motorcycle” but then responded to the test cue "glasses" with the previously non-studied “car," this was counted as correct. Popp and Serra found that the cued recall reverse animacy effect was still present in their overall sample when interference responses (i.e., same-category commission errors) were counted as correct..

Following this example, we recalculated proportion of targets correctly recalled according to this more liberal criterion, and analyzed both the original Popp and Serra data (mixed-lists condition only) and our data. Specifically, we compared mean cued-recall proportion correct (liberal) for animals and objects via paired-samples *t* tests. Figure S7 shows the means and distributions for each condition in both experiments.

**Figure S7**

*Mean proportion of targets correctly recalled (including same-category commission errors) on cued recall by Animacy*

*
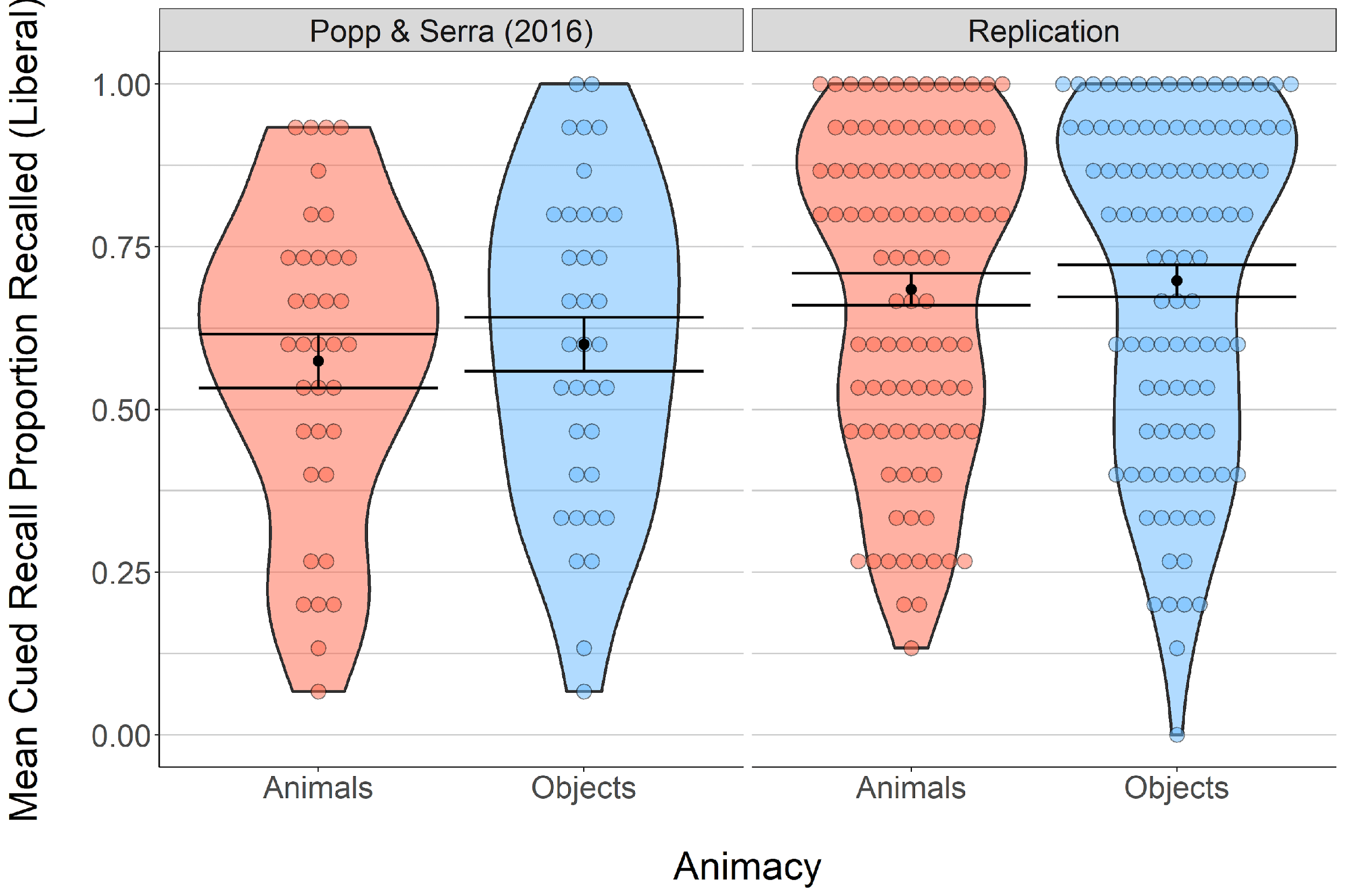
*

*Note.* Means and distributions for cued-recall proportion of targets correctly recalled (liberal criterion including same-category commission errors) across animacy conditions for the original study data (Popp and Serra, 2016, Exp 1, mixed-list condition) and the current replication. Error bars represent 95% within-subject confidence intervals based on the animals-objects comparison for each experiment (calculated as per Loftus & Masson, 1994).

Surprisingly, even in the Popp and Serra (2016) data, the difference was not significant, *t*(35) = .90, *p* = .37, *d*_z_ = .15 [-.18, .48]. It is possible that the overall difference found in their full sample (including the pure-lists condition) was not significant in the smaller mixed-lists subsample due to lower power. However, the difference in our larger sample was also not significant, *t*(100) = .76, *p* = .45, *d*_z_ = .08 [-.12, .27], with Bayesian evidence against a reverse animacy effect.^^[[3]](#footnote-3)^^

In an alternate version of the paired-associates interference analysis reported above, we analyzed proportion correctly recalled using a modified version of the liberal criterion for accuracy. On this criterion, a commission error was counted correct if the recalled word was the same category as the correct target *and* was a word that the participant had seen in the current or previous list (as a cue or target). For example, a test response of the previously unstudied “car” to the studied cue-target pair “glasses – motorcycle” would not be counted as correct, but a response of the previously studied “bicycle”, which appeared as a study word on a prior free recall list, would. Figure S7 below shows the means and distributions for each condition in both experiments.

**Figure S7**

*Mean proportion of targets correctly recalled on cued recall (including within-study same-category commission errors) by Animacy*

*
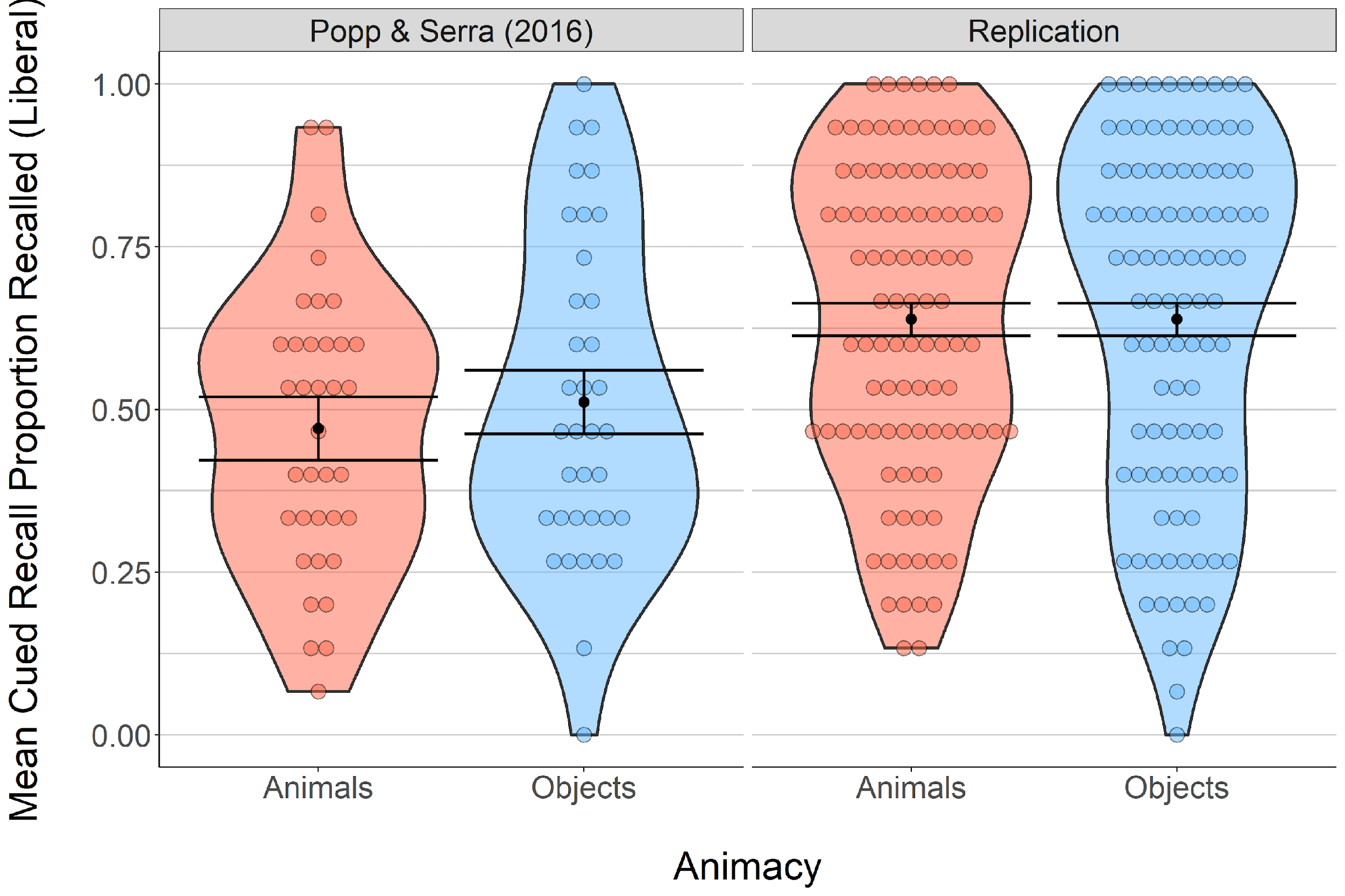
*

*Note.* Means and distributions for cued-recall proportion correctly recalled (liberal criterion) across animacy conditions for the original study data (Popp and Serra, 2016, Exp 1) and the current replication. Error bars represent 95% within-subject confidence intervals based on the animals-objects comparison for each experiment (calculated as per Loftus & Masson, 1994).

In the Popp and Serra data, the difference was not significant, *t*(35) = 1.20, *p* = .24, *d*_z_ = .20 [-.13, .53]. The difference in our sample was also not significant , *t*(100) < .001, *p* = 1, *d*_z_ = 0 [-.20, .20]^^[[4]](#footnote-4)^^. A Bayesian analysis of our data (using the same priors as our main analysis) resulted in a BF of 14.72 *against* a reverse animacy effect. Similar to the main analysis reported in-text, we also observed a BF > 100 *against* a reverse animacy effect similar in magnitude to the original Popp and Serra reverse animacy effect.

1. **Post-study survey**
   1. **Coding scheme**

For all questions except for 2, 5, and 8, a coding scheme was developed by two of the authors (EM and KG). After the coding scheme was finalized, a third author (AC) coded cases in which the first two coders disagreed (84/909 total coding instances). Any cases in which each of the three coders selected a different code were coded as “ambiguous” (39/909 total coding instances).

| **Category awareness** | **Note that mention of specific animals/objects without a mention of a category or subcategory does not count as a mention of animals or objects. There must be some indication that the participant was aware of a general category.** |
| --- | --- |
| *Animals and objects* | Must mention animals (or a subcategory) and objects (or a subcategory) |
| *Animals but not objects* | Must mention animals (or a subcategory), but no objects (or a subcategory) |
| *Objects but not animals* | Must mention objects (or a subcategory), but no animals (or a subcategory) |
| *No category/Other* | Any response that does not include a category (e.g., "All nouns"), or mentions a category that does not fall under animals/objects |
| *No response* |  |
|  |  |
| **Specificity of animal categories** | **Leave blank if they don't mention animals** |
| *Superordinate only* | If they mention only one or more of: Animals, animate words, creatures, living things |
| *Superordinate and subordinate* | If they mention any of the above as well as an animal subcategory (e.g., mammals) |
| *Subordinate only* | If they do not mention any of the superordinate categories but mention one or more subcategories |
| *Single items, no category* | Use if they do not mention a category or subcategory, just single words (e.g., just specific animals) |
|  |  |
| **Specificity of object categories** | **Leave blank if they don’t mention objects** |
| *Superordinate only* | If they mention only one or more of: Objects, everyday objects, household objects, inanimate words, things, items, non-living things |
| *Superordinate and subordinate* | If they mention any of the above as well as an object subcategory (e.g., tools) |
| *Subordinate only* | If they do not mention any of the superordinate categories but mention one or more subcategories |
| *Single items, no category* | Use if they do not mention a category or subcategory, just single words (e.g., just specific objects) |
|  |  |
| **Study strategy** | **If the participant mentioned more than one strategy, code the first and second strategies mentioned** |
| *Imagery (picture, visualize, method of loci)* | If there is mention of an interaction between imagined things, use the "Story" category instead |
| *Rehearsal/Repetition* | "Saying aloud" counts for this one |
| *Relate word to something in one's life* |  |
| *Categorize* |  |
| *Syntactic strategy* | If any non-semantic features of words are used (e.g., letters, acronyms) |
| *Tell story, narrative, song, movie linking words together* |  |
| *Acting out* | E.g., "pantomiming", "hand gestures" |
| *General associative strategy* | Use for non-specific associative strategies (e.g., "I tried to link the words", "I tried to associate the words") |
| *No response or no strategy* | Vague strategies (e.g., "I tried to remember") count for this one |
| *Other* |  |
|  |  |
| **Different strategies for animals/objects?** | **Ignore anything that doesn't related to strategy (e.g., if participant mentions that one category was harder)** |
| *Same* | Use if they do not make explicit mention of categories (e.g., they give a single strategy without mentioning animals/objects) |
| *Different* | Use if they mention doing *anything* different for animals/objects (e.g., if they use imagery for both categories but use a different kind of imagery for each category), or if they only mention doing something for one category. Use even if the participant says "same strategy", but then mentions doing something for animals but not objects (or vice versa). If participants mention separating animals and objects but doing the same thing, counts as "Same". |
| *No response* |  |
| *Other* | "Yes" or "No" answers, or any other answers that give no hint as to whether they mean the same/different (e.g., "sometimes") count for this one. |

- 1. **Category Awareness**

After completing the recall tests, participants were asked to describe the words presented in the experiment (Table 1). Most participants (85.15%) indicated some awareness of both animal and object categories; few participants indicated awareness of animals but not objects (8.91%), objects but not animals (1.98%), or a lack of category awareness (3.96%). Then participants were explicitly asked if they noticed that the words consisted of animals and objects. The responses were 75.2% “Definitely yes”, 19.8% “Maybe yes”, and 1.9% for each of “Maybe not” and “Definitely not” (1 participant failed to select a response).

- 1. **Specificity of Category Awareness**

## Table S3

*Specificity of category awareness: Animals and objects*

| **Specificity** | **Animals %**  **[95% CI]** | **Objects %**  **[95% CI]** | ***𝜒^2^* *p*** |
| --- | --- | --- | --- |
| *Superordinate only* | 76.24 [67.07, 83.48] | 41.58 [32.45, 51.33] | < .001 |
| *Superordinate and subordinate* | 17.82 [11.58, 26.42] | 21.78 [14.85, 30.78] | .60 |
| *Subordinate only* | 0 | 20.79 [14.02, 29.70] | < .001 |
| *Single items, no category* | .99 [.17, 5.40] | 1.98 [.54, 6.93] | 1 |
| *No mention* | 3.96 [1.55, 9.74] | 10.89 [6.19, 18.46] | .11 |
| *Ambiguous* | .99 [.17, 5.40] | 2.97 [1.02, 8.37] | .61 |

We conducted an exploratory analysis comparing the reverse animacy effect across participants who did and did not indicate an awareness of a superordinate object category. If the reverse animacy effect depends in part on category specificity, one might expect to see a smaller reverse animacy effect in participants who viewed objects as a more cohesive, general category. However, we did not find a significant difference in the reverse animacy effect between those who indicated awareness of a superordinate object category (*M* = .09, *SD* = .20) and those who did not (*M* = .10, *SD* = .17), *t*(85.71)^^[[5]](#footnote-5)^^ = 0.23, *p* = .82. The corresponding BF of 19.19 provided further evidence *against* a difference. This suggests that although there were substantial differences in participant awareness of animals and objects as general categories, this difference does not fully explain the reverse animacy effect. This is in line with prior research showing animacy effects that persisted across different levels of category salience (VanArsdall et al., 2017).

- 1. **Self-reported strategy use**

Participants also described the strategies they used for free and cued recall, and whether or not they used different free/cued strategies for animals and objects. For free recall, the most commonly reported strategy was rehearsal/repetition (24.44%), and for cued recall the most commonly reported strategy was imagery (22.73%). However, the only significant difference between strategies was that participants reported using a general associative strategy more often for cued recall (16.67%) than for free recall (5.19%, *Z*-test *p* = .004)—a result that is not particularly surprising. Proportions for all strategies and comparisons across test type are reported below.

**Table S4**

*Self-reported strategy use by memory type*

| **Strategy** | **Free %**  **[95% CI]** | **Cued %**  **[95% CI]** | ***Z-*test *p*** |
| --- | --- | --- | --- |
| *Rehearsal/Repetition* | 24.44 [17.97, 2.33] | 13.64 [8.80, 20.53] | .04 |
| *Story/narrative/song* | 22.96 [16.68, 30.75] | 15.91 [10.65, 23.10] | .19 |
| *Imagery* | 17.78 [12.24, 25.09] | 22.73 [16.41, 30.59] | .39 |
| *Categorize* | 6.67 [3.55, 12.18] | 2.27 [.78, 6.47] | .15 |
| *General associative strategy* | 5.19 [2.53, 10.32] | 16.67 [11.27, 23.95] | .005* |
| *Relate word to something in one’s life* | 5.19 [2.53, 10.32] | 0 | .05 |
| *Syntactic strategy* | 2.96 [1.16, 7.37] | 9.09 [5.28, 15.22] | .06 |
| *Acting out* | 1.48 [.41, 5.24] | .76 [.13, 4.17] | 1 |
| *Ambiguous* | 9.63 [5.71, 15.78] | 15.15 [10.03, 22.25] | .24 |
| *No response/No strategy* | 3.70 [1.59, 8.38] | 3.79 [1.63, 8.56] | 1 |

*Note*. * = Significant after controlling for multiple tests via Bonferroni correction

We also asked participants whether they used the same strategies for animals and objects, separately for free and cued recall. For both free and cued recall, the majority of participants reported using the same strategy for animals and objects (63.37% for free, 57.42% for cued). There were no significant differences in the proportions of same/different strategy reporting between free and cued recall:

**Table S5**

*Self-reported differential strategy use by memory type*

| **Used different strategies for animals and objects?** | **Free %**  **[95% CI]** | **Cued %**  **[95% CI]** | ***Z-*test *p*** |
| --- | --- | --- | --- |
| *Same* | 63.37 [53.64, 72.11] | 57.43 [47.69, 66.62] | .47 |
| *Different* | 17.82 [11.58, 26.42] | 15.84 [9.99, 24.19] | .85 |
| *Other* | 8.91 [4.76, 16.07] | 12.87 [7.68, 20.78] | .50 |
| *Ambiguous* | 1.98 [.54, 6.93] | 0 | .48 |
| *No response* | 7.92 [4.07, 14.86] | 13.86 [8.44, 21.93] | .26 |

Thus, a strategy-based explanation for the reverse animacy effect might be the following: most participants adopt a general strategy (e.g., imagery) for cued recall, and this strategy is less effective for animal pairs than object pairs. To explore this possibility further, we separated participants into those who used the same strategy for cued recall of animals and objects and those who reported using different strategies, and compared the magnitude of the reverse animacy effect. If the above explanation is correct, we might expect to see a smaller reverse animacy effect (or a normal animacy effect) in participants who reported differential strategy use. However, we did not find a significant difference in the reverse animacy effect between those who used the same strategy (*M* = 10, *SD* = 17.76) and those who used different strategies (*M* = 9.17, *SD* = 22.03), *t*(20.69)^^[[6]](#footnote-6)^^ = 0.14, *p* = .89^^[[7]](#footnote-7)^^. The corresponding BF of 21.27 in favor of a null effect provided further evidence against a difference. This suggests that either 1) participants strategy switching was ineffective in combating the reverse animacy effect, 2) the reverse animacy effect has less to do with strategy and more to do with inherent category features (e.g., animal words reduce cued recall performance across a wide range of strategies), or 3) retrospective self-reports of strategy may have low validity. Further research directly manipulating strategy use could help disentangle these two possibilities.

- 1. **Self-reported relative recall difficulty**

We examined participants’ ratings of perceived ease of recalling animals relative to objects for each memory task (Figure 3). Values to the right of the dashed line (from 0 to 50) indicate greater ease for animals, values to the left of the dashed line (from 0 to -50) indicate greater ease for objects. On average, participants indicated greater ease for recalling animals compared to objects on both the free recall task (*M* = 13.2, *SD* = 24.3) and the cued recall task (*M* = 6.7, *SD* = 21.4). A paired-samples *t* test^^[[8]](#footnote-8)^^ indicated that the reported relative ease of animate nouns was higher for the free recall task, *t*(100) = 2.60, *p* = .01, *d*_z_ =.26 [.06 .46]. A Bayesian version of this analysis revealed extreme evidence that participants viewed animals as easier to recall than objects for free recall (Savage-Dickey BF > 100), and moderate evidence that this difference was larger for free than cued recall (Savage-Dickey BF = 3.13). However, we also found extreme evidence that participants viewed animals as easier to recall than objects for cued recall (Savage-Dickey BF > 100). Finally, we examined the relationship between relative recall performance (e.g., the size of animacy/reverse animacy effects) and self-reported relative recall difficulty. As detailed in SOM (Section F4), these unplanned exploratory analyses yielded mixed evidence for a relationship, suggesting that participants’ self-reports imperfectly tracked actual performance.

## Figure S8

*Perceived ease of recalling each word type by memory test type*


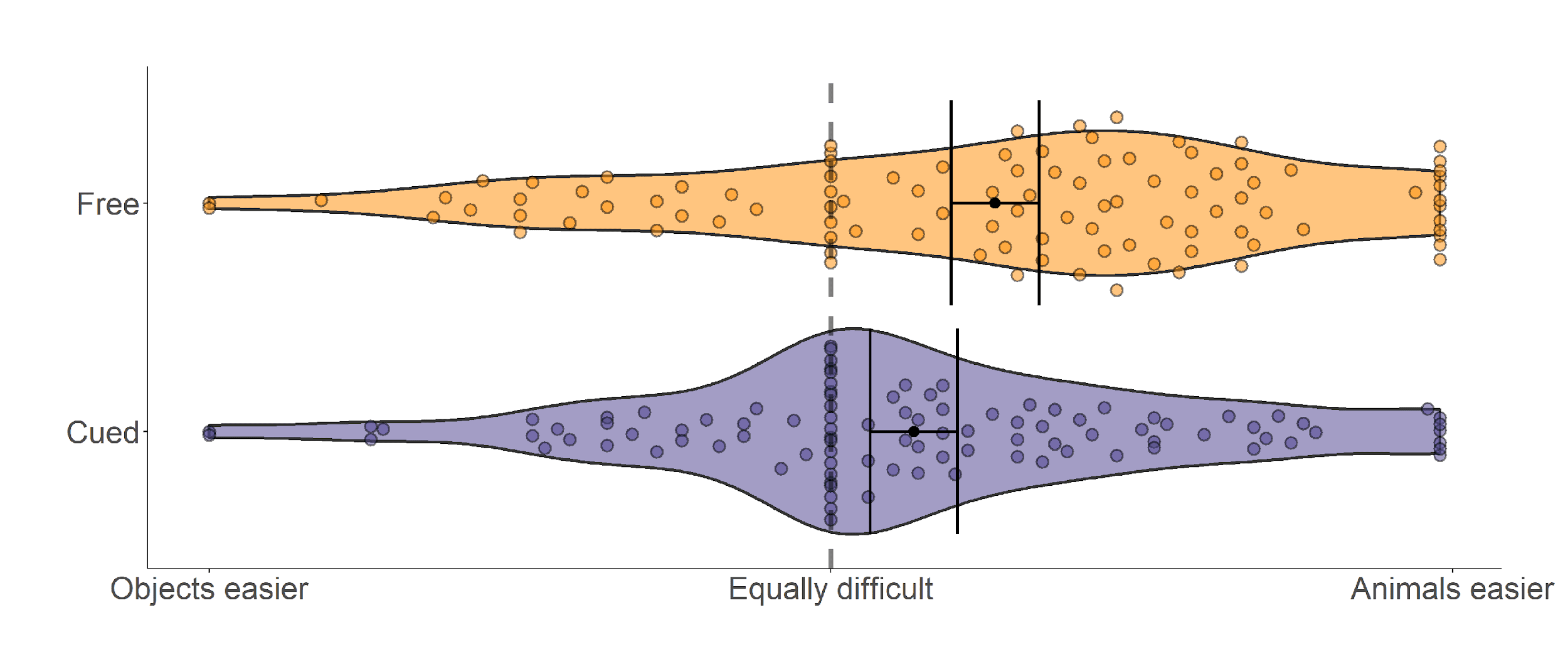


*Note.* Means and distributions for perceived ease of recalling each word type by memory test type. Positive values indicate greater ease for animals, whereas negative values indicate greater ease for objects. Error bars show 95% within-subject confidence intervals.

We were also interested in whether participant reports of relative recall difficulty were calibrated with their performance (e.g., whether participants who recalled many more objects than animals also reported that objects were easier to recall than animals). To investigate this, we conducted NHST and Bayesian regressions predicting relative recall difficulty from relative recall performance (i.e., proportion recalled animals - proportion recalled objects), by memory type. If participant self-reports were calibrated with their performance, we would expect a significant positive relationship. This relationship was not statistically significant, 𝛽 = .19, *t*(180) = 1.73, *p* = .09, but was supported by Bayesian evidence (BF = 6.67). The interaction between relative recall performance and memory type was not statistically significant, 𝛽 = .08, *t*(154) = .51, *p* = .61, but Bayesian evidence against an interaction was only anecdotal (BF = 1.78). Though these results are not conclusive, they suggest that participant meta-memorial reports of recall difficulty were calibrated with their performance, and that this calibration was similar for free and cued recall. Figure S8 below shows relative recall difficulty by relative recall performance for free and cued recall.

##

## Figure S8

*Relationship between self-reported relative recall difficulty and relative recall performance*


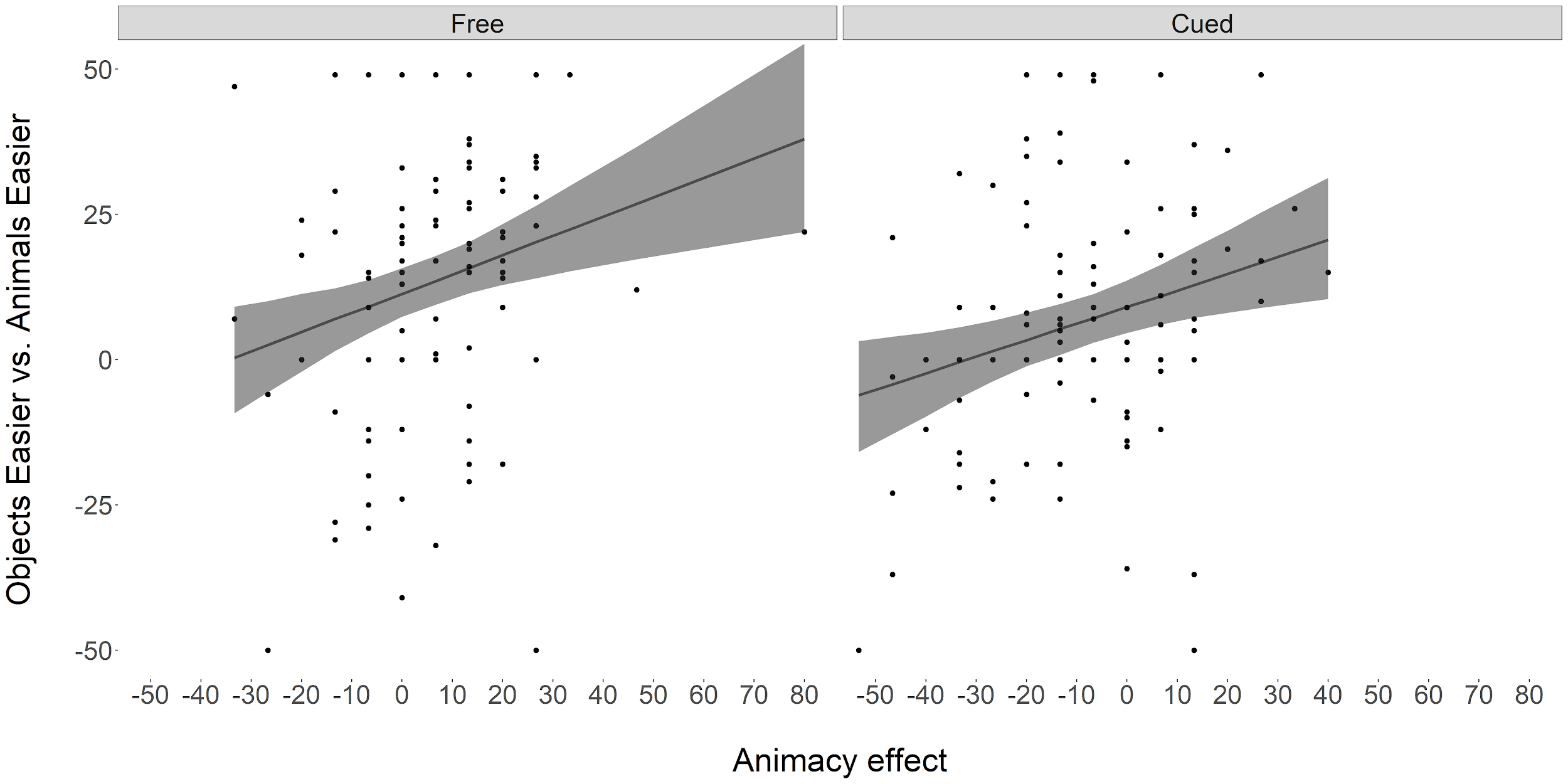


*Note.* Points represent individual participants. Regression lines plotted with 95% credible intervals on the fit line. Estimates based on the Bayesian regression model. See figure S4A, S4B, and S4C for a visual depiction of model priors.

## 2. Experiment 2 (Samples 2-5)

### Sample exclusions and statistics

|  |  | Exclusion criteria | | | | | | | Final samples | | | | |
| --- | --- | --- | --- | --- | --- | --- | --- | --- | --- | --- | --- | --- | --- |
| Sample^^[[9]](#footnote-9)^^ | Total *N* | Major distraction* | Cheating* | Understood < 75% of words* | ESL* | Technical Difficulty* | 0 correct on any list | ≥ 5 “fast skips” on any CR list | Final *N* | “Animals-more-similar” | FR 1st | Age *M* (SD) | ESL |
| Undergraduate 1 | 179 | 7 | 0 | 18 | N/A | 5 | 3 | 6 | 150 | 68 | 84 | 21.7 (5.7) | 6% |
| Undergraduate 2 | 176 | 5 | 2 | 10 | N/A | 5 | 8 | 11 | 143 | 72 | 74 | 21.4 (5) | 15% |
| Prolific | 134 | 2 | 6 | 16 | N/A | 0 | 11 | 10 | 101 | 55 | 61 | 26.7 (7.6) | 63% |
| Prolific (EFL) | 180 | 0 | 1 | 7 | 4 | 2 | 17 | 9 | 150 | 79 | 72 | 29 (9.1) | 0% |

*Note.* * denotes self-report questions. A CR “fast skip” was any CR response where the participant pressed “Enter” without typing a CR response in under 1s. Note that some participants met multiple exclusion criteria. Differing final *N*’s are due to differential sequential stopping points (see the OSF wiki page at <https://osf.io/k4emy/?view_only=0d5ffa55a8e040fb9b16a478cb2cd2c1> for a record of sequential tests for each sample).

### Word-selection process

The following details the word selection process for our study, which examined the degree to which category semantic structure explains the (reverse) animacy effect.

First, we gathered an initial wordset of animals and objects:

● A dictionary of animals was obtained from the [SemNetDictionaries](https://cran.r-project.org/web/packages/SemNetDictionaries/SemNetDictionaries.pdf) R package (dictionary created via Google, thesaurus, semantic fluency data).

● A dictionary of objects was obtained from the [THINGS](https://journals.plos.org/plosone/article?id=10.1371/journal.pone.0223792) database.

○ From this database, we removed words from the following categories: animals, fruits, nuts, plants, insects, fossils, body parts, humans, food, wood, lumber, fungus, dirt, flowers, trees, and seeds, as well as any words including a hyphen or space.

● Category exemplar generation data from the classic Battig & Montague wordset (from the [WordPools](https://rdrr.io/cran/WordPools/man/Battig.html) R package), as well as from a Canadian norming study from [Kantner & Lindsay](https://onlineacademiccommunity.uvic.ca/lindsaylab/wp-content/uploads/sites/4861/2020/08/Kantner-and-Lindsay-2014-Canadian-Category-Norms.pdf)

○ From both datasets, we obtained category exemplars generated for animals (Categories: bird, fish, four-footed animal, insect, snake) and for objects (Categories: article of clothing, article of furniture, carpenter’s tool, kind of money, kitchen utensil, musical instrument, precious stone, toy, type of footgear, type of human dwelling, type of reading material, type of ship, type of vehicle, weapon)

○ Then, we generated lists of animals and objects that were *not* already in the animal/object dictionaries. Eric went through these lists and subjectively excluded items that he deemed not suitable (e.g., plural forms of words already in the database, words that had dual meanings that made category ambiguous, words unlikely to be recognized by undergraduates, words that were not concrete nouns). The remaining words were then added to the dictionaries (first from Battig & Montague, then checking again for novel items in Kantner & Lindsay).

● Then, Eric, Kelly & Steve reviewed the updated dictionaries for further potential exclusions (using similar criteria as above). The following further manual exclusions were made, again on somewhat subjective determinations:

○ Animals: "lioness" (cf., “lion”), "kiwi", "feline", "fowl", "bass", "rhinoceros" (cf. “rhino”), "hippopotamus" (cf. “hippo”), "mermaid","queen", "human", "centaur","dragon", "monitor", "narwhale" (cf. “narwhal”), "pegasus","ragdoll", "sphinx", "tiffany","unicorn", "amphibian", "reptile", "pet","arachnid", "bitch", "coral", "dinosaur","insect", "mammal", "marsupial", "mice", "molly","moccasin", "neanderthal", "newfoundland", "pointer","pup", "rodent", "sasquatch", "seafish",“sponge", "steer", "tigress" (cf. “tiger”), "tom", "bat", "mouse","boa", "boxer", "bug", "crane", "jack", "jenny", "joey","lab", "pike", "seal", "budgerigar" (cf. “budgie”), "yeast", "rock", “ass”, “fish”, “mole”, “bird”

○ Objects: "bat", "boa", "crane", "fungus","mistletoe", "moccasin", "mold","tool", "jack", "mouse", "seal", "buoy","chalice", "wreck", "camper", "garter","honeycomb", "kite", "organ", "pulpit","stand", "slip", "bass", "bicycle" (cf. “bike”)

● After these exclusions, our dictionary consisted of 611 animal words and 1205 object words

## Then, we gathered databases of relevant word characteristics, and reduced our wordset to those with all relevant characteristics

● The following characteristics were chosen on the basis of previous literature and [a recent study examining the relationships between semantic properties and free recall probability](https://link.springer.com/article/10.3758/s13423-020-01820-w) (Madan, 2020) (specifically, those characteristics that were significantly correlated with recall probability in both studies examined in the paper, and those characteristics unrelated to animacy):

○ Word frequency (from the [SUBTLEXUS database](http://www.lexique.org/?page_id=241))

○ Contextual diversity (from the [SUBTLEXUS database](http://www.lexique.org/?page_id=241))

○ Age of Acquisition (from [Kuperman et al., 2012](https://link.springer.com/article/10.3758/s13428-012-0210-4))

○ Imageability (from the [Glasgow Norms](https://link.springer.com/article/10.3758/s13428-018-1099-3))

● Alongside these characteristics, we were primarily interested in the within-category similarity relationships. So, we obtained a semantic space “created from a 2 Billion word corpus, which was created by concatenating the British National Corpus (BNC), the ukWaC corpus and a 2009 Wikipedia dump.” (EN_100k_lsa, more details [here](https://sites.google.com/site/fritzgntr/software-resources/semantic_spaces))

● We then cross-referenced our dictionaries with these databases, including only words that appeared in all of the databases. This resulted in a reduced dictionary of 141 animals and 451 objects.

## Next, we generated a large number of randomly sampled wordsets and obtained various statistics for the sets

● Specifically, over 500,000 iterations, we randomly sampled 48 animal words and 48 object words (the required words for our experiment design) from our reduced dictionary. For each list and each category, we obtained:

○ The within-category pairwise semantic similarities

○ Word frequency

○ Contextual diversity

○ Age of Acquisition

○ Imageability

● From the above, we derived:

○ The [assumption-free distributional overlap index](https://jackedtaylor.github.io/2020/07/06/item-wise-and-distribution-wise-matching/#distribution-wise-matching) between the animal and object samples, for each of the characteristics

● And crucially:

○ The difference in mean animal pairwise semantic similarity and mean object pairwise semantic similarity

## We then searched the generated set for wordsets that met our needs

● First, we restricted the lists to those in which the average overlap (across word frequency, contextual diversity, age of acquisition, and imageability) was above the 97.5th percentile, and for which the animal semantic similarity advantage was above the 97.5th percentile (i.e., “Animal similarity advantage” sets). Then, the same restrictions were applied to the reduced set to find the best sets.

○ From these lists, Eric selected one that:

■ Had a large animal similarity advantage (10th highest out of 245 candidate sets at .25)

■ Had “good” overlap on the relevant characteristics (all overlaps over .60)

■ Did not contain any potentially problematic words

● Next, we restricted the lists based on the same criteria above, but now to those in which the average overlap was above the 97.5th percentile and the animal semantic similarity advantage was below the 2.5th percentile (i.e., an “Equal similarity” set^[1]^). For the second pass, we used the 90th percentile for overlap because there were no sets that met the criteria when the 97.5th percentile was used.

○ For each of these “Equal similarity” candidate sets, we extracted the animal and object lists and compared their characteristic distributions to those in our chosen animal set, via the same distribution overlap index. The objective here was to ensure that our “Equal similarity” set was not radically different from our “Animals-more-similar” set in terms of word frequency, age of acquisition, context diversity, or imageability.

■ Based on these results, Eric selected the set with the lowest animal similarity advantage (at .04)--although this set did not have the highest distributional overlap with the “Animals-more-similar” set, the priority was on ensuring a maximally strong manipulation. Despite this, all distributional overlap indices were above .60.

● The final “Animals-more-similar” and “Equal” wordsets are shown below:

| **word** | **CATEGORY** | **Similarity Condition** |
| --- | --- | --- |
| trout | animals | Animals-more-similar |
| ostrich | animals | Animals-more-similar |
| pig | animals | Animals-more-similar |
| hen | animals | Animals-more-similar |
| calf | animals | Animals-more-similar |
| mink | animals | Animals-more-similar |
| gorilla | animals | Animals-more-similar |
| kangaroo | animals | Animals-more-similar |
| tiger | animals | Animals-more-similar |
| giraffe | animals | Animals-more-similar |
| hippo | animals | Animals-more-similar |
| hog | animals | Animals-more-similar |
| termite | animals | Animals-more-similar |
| bull | animals | Animals-more-similar |
| tortoise | animals | Animals-more-similar |
| chipmunk | animals | Animals-more-similar |
| weasel | animals | Animals-more-similar |
| yak | animals | Animals-more-similar |
| toad | animals | Animals-more-similar |
| rhino | animals | Animals-more-similar |
| seahorse | animals | Animals-more-similar |
| eel | animals | Animals-more-similar |
| sheep | animals | Animals-more-similar |
| rat | animals | Animals-more-similar |
| snake | animals | Animals-more-similar |
| shrew | animals | Animals-more-similar |
| deer | animals | Animals-more-similar |
| goose | animals | Animals-more-similar |
| maggot | animals | Animals-more-similar |
| ladybird | animals | Animals-more-similar |
| lynx | animals | Animals-more-similar |
| crow | animals | Animals-more-similar |
| pony | animals | Animals-more-similar |
| shrimp | animals | Animals-more-similar |
| butterfly | animals | Animals-more-similar |
| gerbil | animals | Animals-more-similar |
| bee | animals | Animals-more-similar |
| chameleon | animals | Animals-more-similar |
| donkey | animals | Animals-more-similar |
| ferret | animals | Animals-more-similar |
| duck | animals | Animals-more-similar |
| pheasant | animals | Animals-more-similar |
| porpoise | animals | Animals-more-similar |
| chimpanzee | animals | Animals-more-similar |
| mule | animals | Animals-more-similar |
| wolf | animals | Animals-more-similar |
| wasp | animals | Animals-more-similar |
| gazelle | animals | Animals-more-similar |
| book | objects | Animals-more-similar |
| curb | objects | Animals-more-similar |
| dictionary | objects | Animals-more-similar |
| amplifier | objects | Animals-more-similar |
| swing | objects | Animals-more-similar |
| cello | objects | Animals-more-similar |
| ski | objects | Animals-more-similar |
| bus | objects | Animals-more-similar |
| bullet | objects | Animals-more-similar |
| altar | objects | Animals-more-similar |
| scooter | objects | Animals-more-similar |
| umbrella | objects | Animals-more-similar |
| candle | objects | Animals-more-similar |
| fuse | objects | Animals-more-similar |
| trumpet | objects | Animals-more-similar |
| pulley | objects | Animals-more-similar |
| grenade | objects | Animals-more-similar |
| vessel | objects | Animals-more-similar |
| roof | objects | Animals-more-similar |
| beaker | objects | Animals-more-similar |
| blouse | objects | Animals-more-similar |
| speaker | objects | Animals-more-similar |
| palace | objects | Animals-more-similar |
| inn | objects | Animals-more-similar |
| projector | objects | Animals-more-similar |
| tray | objects | Animals-more-similar |
| ladder | objects | Animals-more-similar |
| accordion | objects | Animals-more-similar |
| cane | objects | Animals-more-similar |
| submarine | objects | Animals-more-similar |
| bowl | objects | Animals-more-similar |
| triangle | objects | Animals-more-similar |
| piano | objects | Animals-more-similar |
| shoe | objects | Animals-more-similar |
| seesaw | objects | Animals-more-similar |
| scale | objects | Animals-more-similar |
| spear | objects | Animals-more-similar |
| arrow | objects | Animals-more-similar |
| crown | objects | Animals-more-similar |
| ferry | objects | Animals-more-similar |
| wood | objects | Animals-more-similar |
| fork | objects | Animals-more-similar |
| ruler | objects | Animals-more-similar |
| harpsichord | objects | Animals-more-similar |
| fireworks | objects | Animals-more-similar |
| flask | objects | Animals-more-similar |
| sail | objects | Animals-more-similar |
| puzzle | objects | Animals-more-similar |
| caterpillar | animals | Equal |
| lark | animals | Equal |
| owl | animals | Equal |
| walrus | animals | Equal |
| lynx | animals | Equal |
| tarantula | animals | Equal |
| bull | animals | Equal |
| sheep | animals | Equal |
| moose | animals | Equal |
| mussel | animals | Equal |
| deer | animals | Equal |
| giraffe | animals | Equal |
| shark | animals | Equal |
| pheasant | animals | Equal |
| slug | animals | Equal |
| goose | animals | Equal |
| penguin | animals | Equal |
| rooster | animals | Equal |
| cricket | animals | Equal |
| anteater | animals | Equal |
| butterfly | animals | Equal |
| prawn | animals | Equal |
| stag | animals | Equal |
| beetle | animals | Equal |
| chameleon | animals | Equal |
| mule | animals | Equal |
| dove | animals | Equal |
| puffin | animals | Equal |
| sow | animals | Equal |
| python | animals | Equal |
| lizard | animals | Equal |
| moth | animals | Equal |
| ladybird | animals | Equal |
| hog | animals | Equal |
| hedgehog | animals | Equal |
| chimpanzee | animals | Equal |
| frog | animals | Equal |
| kangaroo | animals | Equal |
| antelope | animals | Equal |
| ostrich | animals | Equal |
| oyster | animals | Equal |
| hen | animals | Equal |
| louse | animals | Equal |
| lobster | animals | Equal |
| squid | animals | Equal |
| porpoise | animals | Equal |
| mosquito | animals | Equal |
| starfish | animals | Equal |
| foam | objects | Equal |
| noose | objects | Equal |
| trailer | objects | Equal |
| dictionary | objects | Equal |
| ceiling | objects | Equal |
| veil | objects | Equal |
| blade | objects | Equal |
| bandage | objects | Equal |
| crown | objects | Equal |
| vacuum | objects | Equal |
| harmonica | objects | Equal |
| window | objects | Equal |
| compass | objects | Equal |
| pocket | objects | Equal |
| balloon | objects | Equal |
| paperclip | objects | Equal |
| pencil | objects | Equal |
| strap | objects | Equal |
| whip | objects | Equal |
| girdle | objects | Equal |
| scalpel | objects | Equal |
| dresser | objects | Equal |
| duster | objects | Equal |
| cottage | objects | Equal |
| keg | objects | Equal |
| trunk | objects | Equal |
| hovercraft | objects | Equal |
| gold | objects | Equal |
| cushion | objects | Equal |
| train | objects | Equal |
| chime | objects | Equal |
| hat | objects | Equal |
| handle | objects | Equal |
| sock | objects | Equal |
| pamphlet | objects | Equal |
| flask | objects | Equal |
| mallet | objects | Equal |
| toothbrush | objects | Equal |
| shawl | objects | Equal |
| bulldozer | objects | Equal |
| badge | objects | Equal |
| mask | objects | Equal |
| crank | objects | Equal |
| drawers | objects | Equal |
| cord | objects | Equal |
| mug | objects | Equal |
| screwdriver | objects | Equal |
| chip | objects | Equal |

● Figure A1 depicts the distributions of our wordsets and categories.

**Figure A1**

*Distributions for the wordsets and categories*


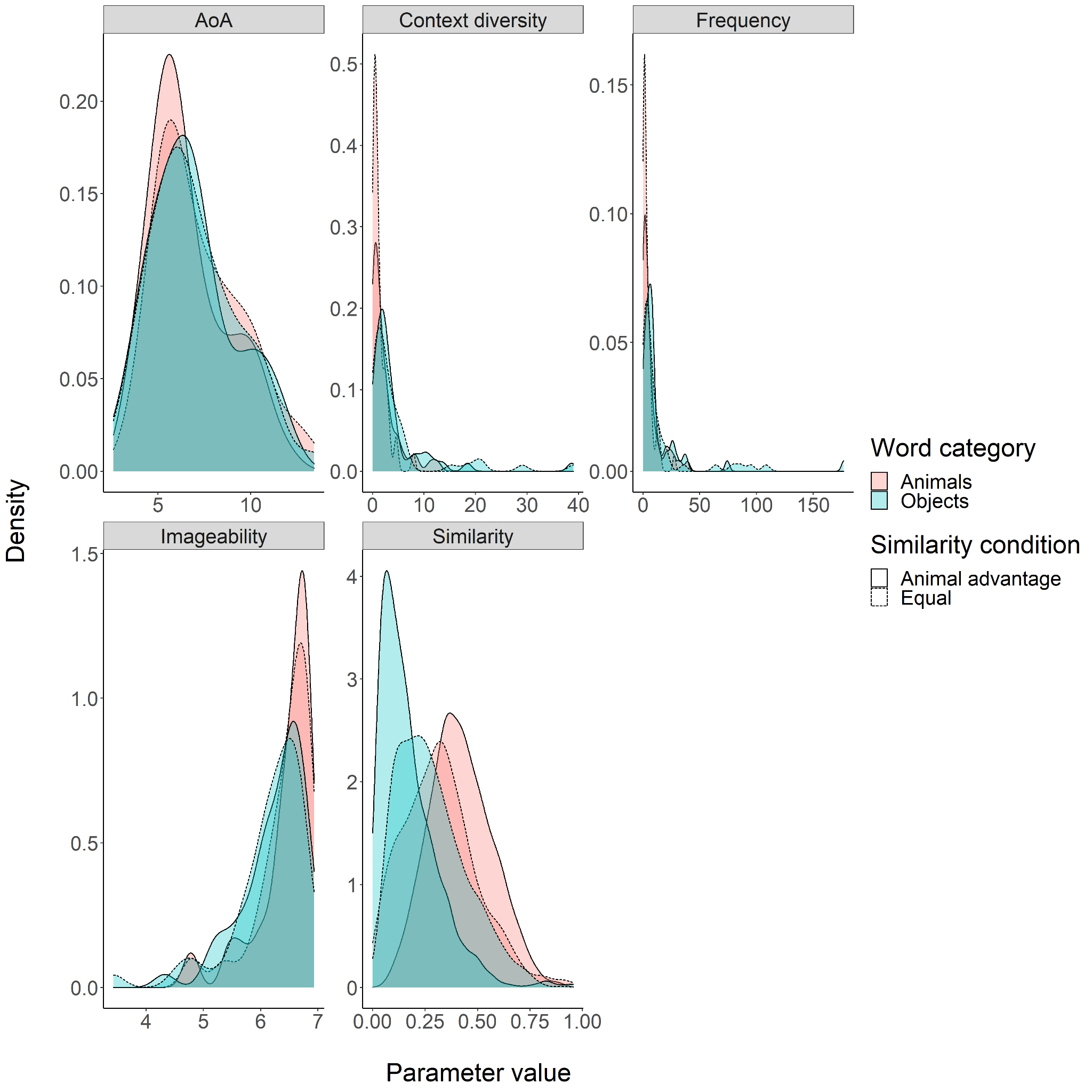


● For most characteristics, overlap across categories and conditions is good (though CD and frequency seem to be a bit lower for animals, and imageability seems to be a bit lower for objects). Importantly, we see a difference in similarity across the similarity conditions (though perhaps not as strong as we would like).

## From our final wordset, we examined the properties of randomly-generated individual experiment lists

● That is, we repeated the same random sampling process, this time drawing 10,000 sets of 8 animals and 8 objects (the minimum size of an experiment wordlist, maximum being double that) from our final wordsets (“Animals-more-similar”, “Equal”). The objective here was to see whether individual lists that participants might see roughly met our criteria.

○ First, for the Animals-more-similar set, we examined the distribution of listwise differences in word frequency, age of acquisition, context diversity, and imageability. The objective here was to make sure that on average, lists were not unduly biased toward animals or objects on a given characteristic

■ The distribution of listwise differences are plotted below (figure A2):

**Figure A2**

*Distribution of listwise differences for Animals-more-similar wordset*

**
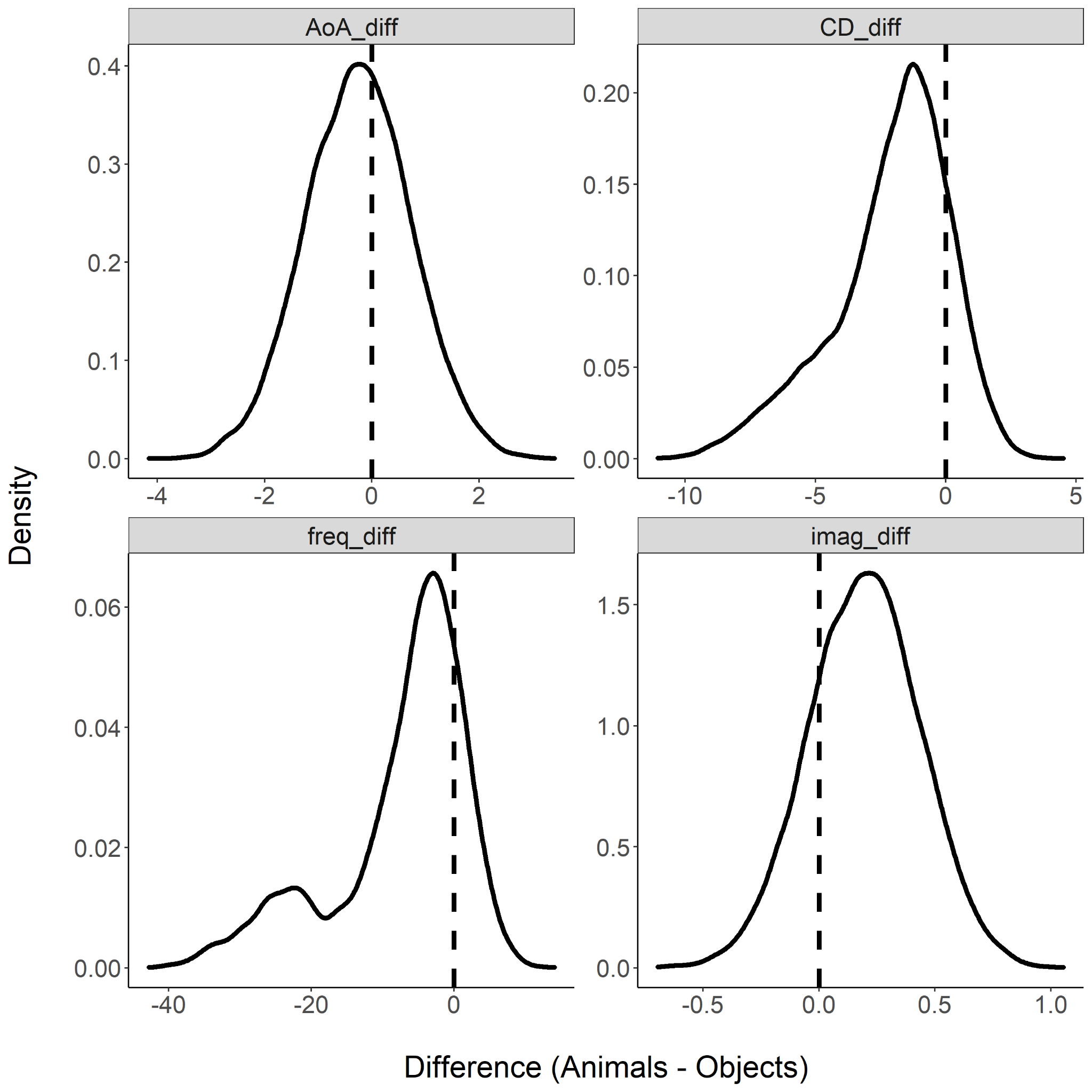
**

Not ideal for context diversity and frequency (both skewed slightly towards objects), but most of the differences are clustered near zero.

○ Then, we did the same thing for the Equal wordset (figure A3)

**Figure A3**

*Distribution of listwise differences for Equal similarity wordset*

*
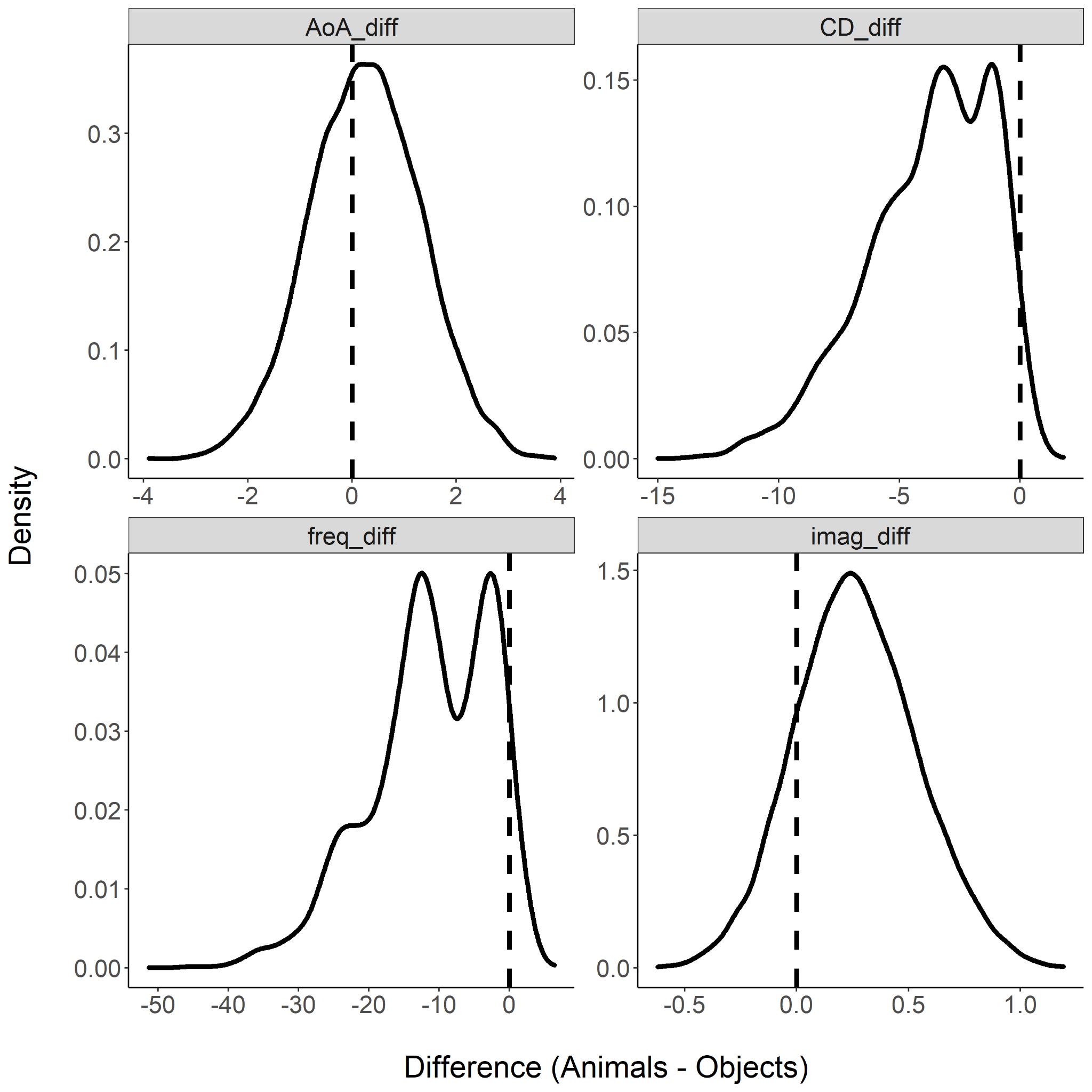
*

■ Things again appear to be less than ideal for context diversity and frequency. Maybe it is difficult to manipulate the relative animal-object similarity without shifting frequency and contextual diversity?

■ For both sets of previous study words, we observed similar distributions of listwise differences as our new lists (i.e., contextual diversity and frequency disadvantages for animals, imageability advantage for animals)

○ For both of our new wordsets, we were also interested in the listwise distributional overlap between the 8 animal and 8 object words on the salient characteristic. Those results are shown below (figure A4):

**Figure A4**

*Listwise distributional overlap between a random sample of 8 animal and 8 object words*


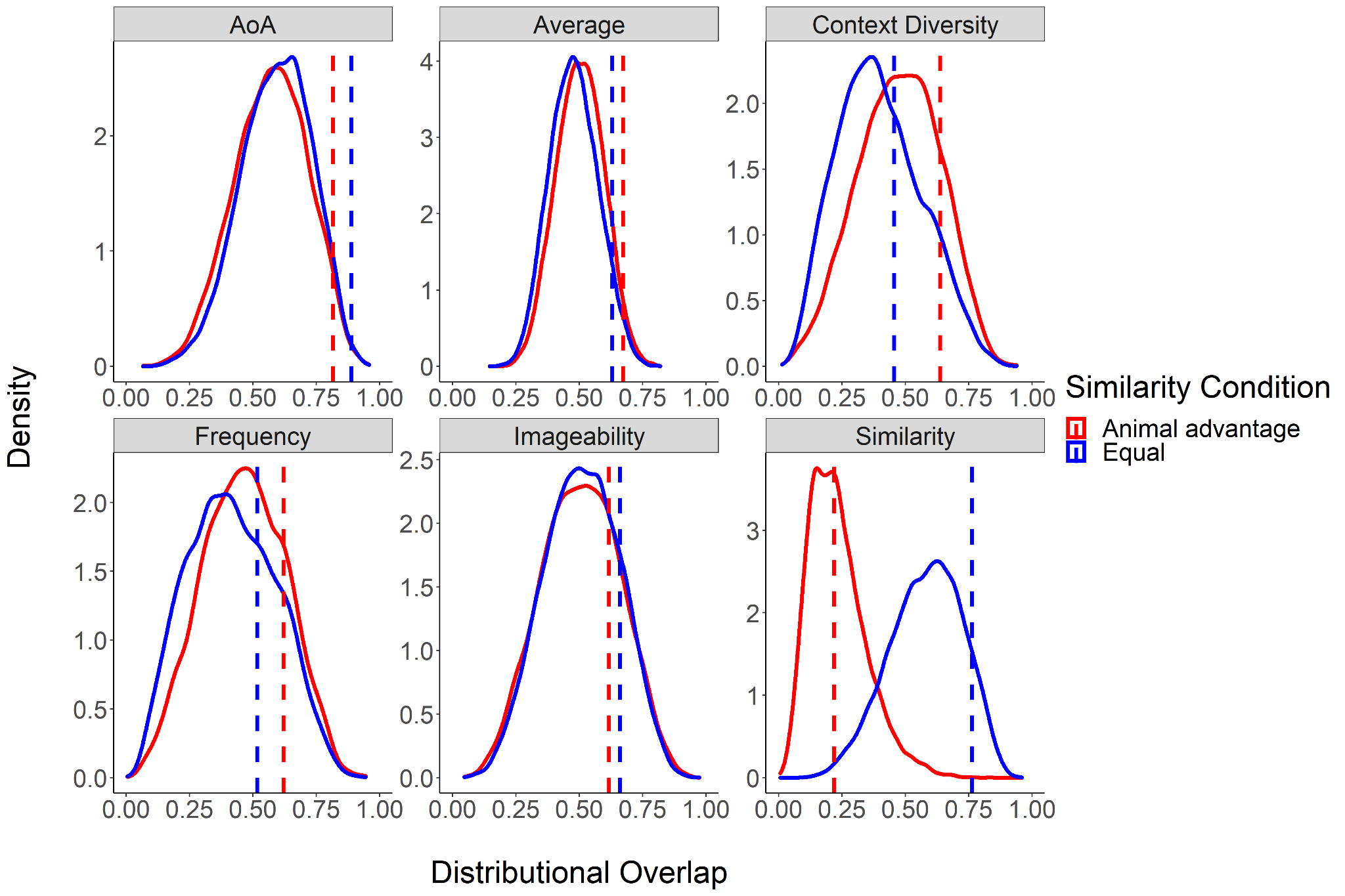


■ The vertical lines here represent the distributional overlap value in the overall wordsets, with the distributions representing the listwise results. Overall, distributional overlap tends to be lower for the sublists than the overall list (harder to get stable distributions with 16 than 96 words, probably), and overlap ranges from decent to poor. So again, not ideal (except for similarity, which suggests that our manipulation might work as intended).

○ Finally, we were interested in our proposed manipulation at the listwise level. From the above randomly-generated sample, we computed listwise animal similarity advantages (avg. pairwise similarity for the 8 animal words - avg. pairwise similarity for the 8 object words). We also went back into our data from the previous study (using the [Popp and Serra](https://citeseerx.ist.psu.edu/viewdoc/download?doi=10.1.1.1059.8326&rep=rep1&type=pdf) wordlist) and calculated listwise animal similarity advantages for all the lists that appeared in the study. We then plotted our proposed manipulation conditions with the previous study data (figure A5 and figure A6):

**Figure A5**

*Listwise animal similarity advantages for randomly-generated sample in Figure A4*


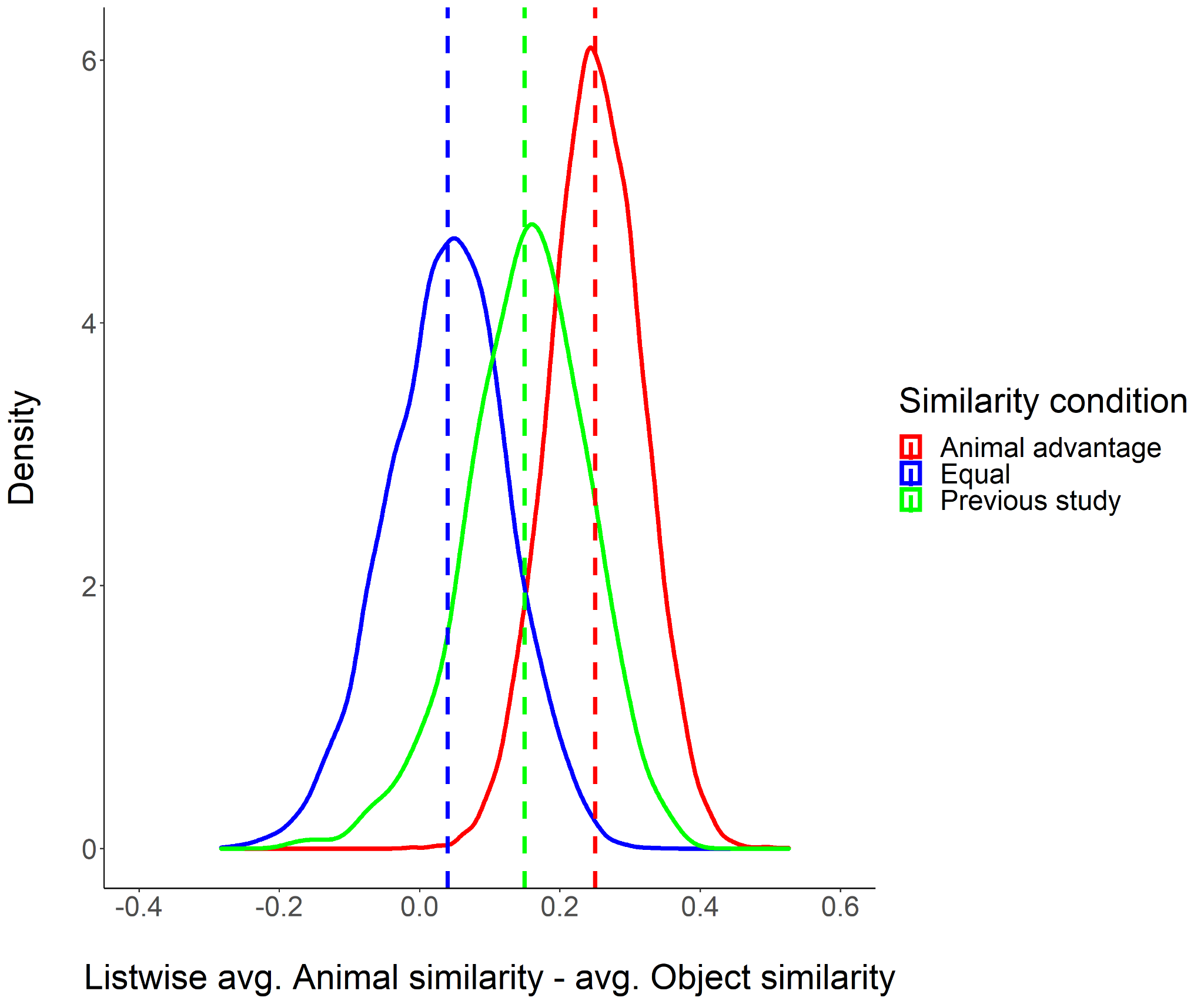
(Note: The above figure uses lists of only size 16 for the new conditions, and lists of size 15 and 30 for the previous study, i.e., cues and targets were included. For the same thing limited to only previous study targets, i.e., lists of 15, see the figure below)

**Figure A6**

**
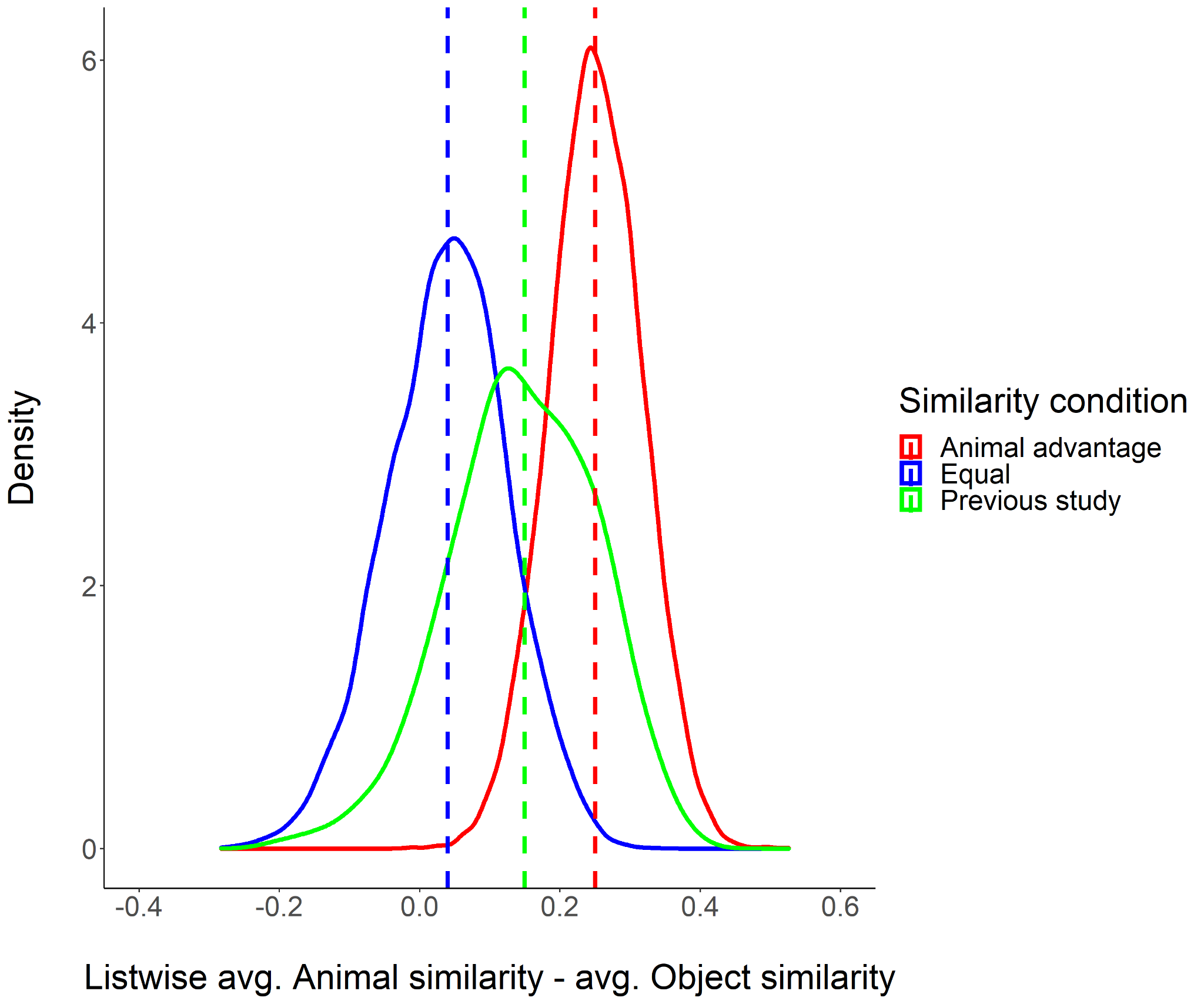
**

As we can see, it does seem like our manipulation conditions represent reasonably different similarity relationships at the list-level. Interestingly, the wordset used in our previous studies seems to fall almost exactly between the proposed manipulations for the current study.

^[1]^ The reader may wonder why we did not adopt the stronger manipulation of an “Object similarity advantage” set, as was initially planned. This is because out of the 500,000 randomly sampled wordsets, *no* set had objects with average pairwise similarity higher than the animals in the set. This may be a quirk of our sampling process or dictionaries, or because higher semantic similarity is an inherent property of animal words.

### Figures for individual samples

- 1. Undergraduate 1


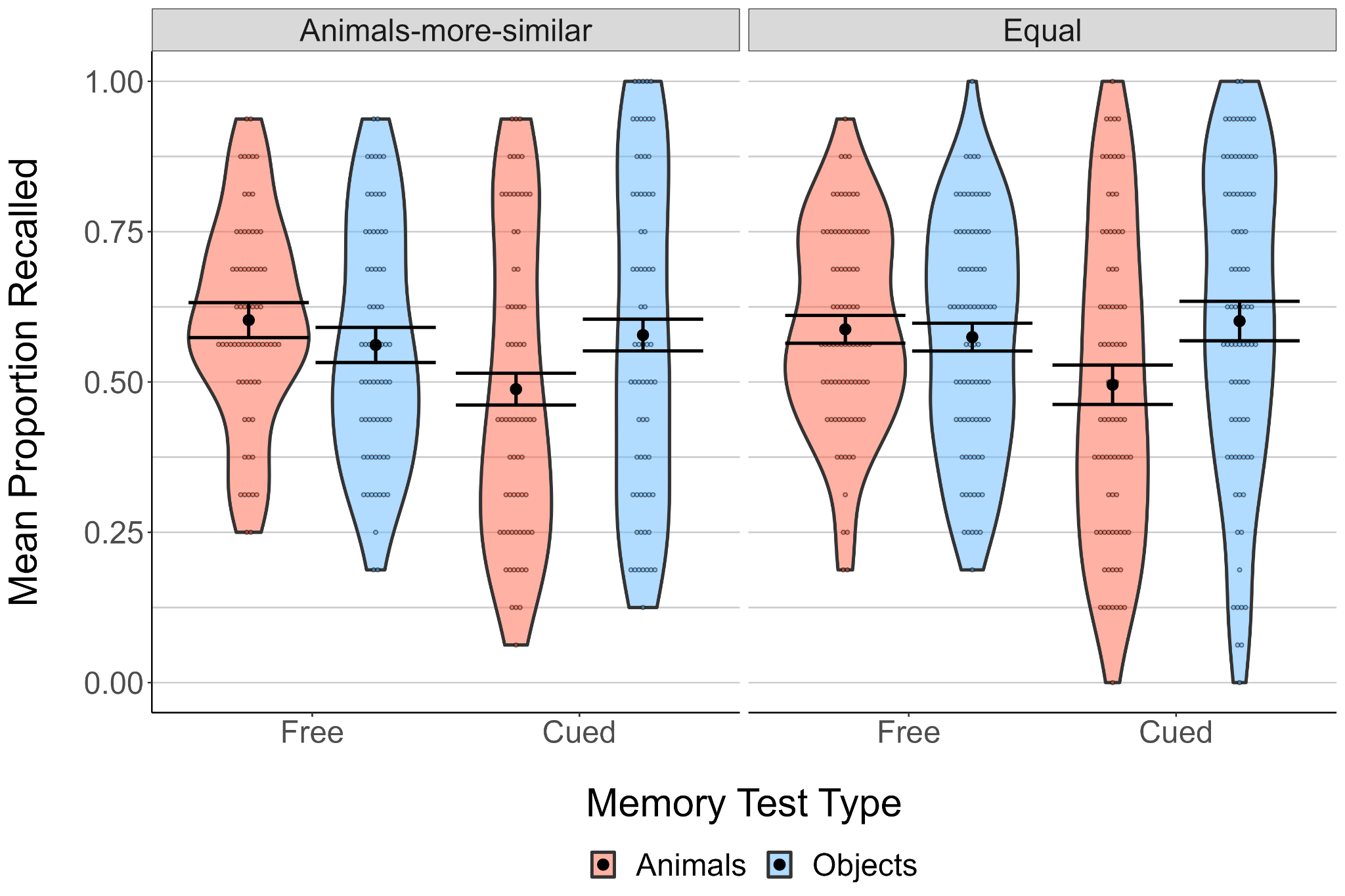


- 1. Undergraduate 2


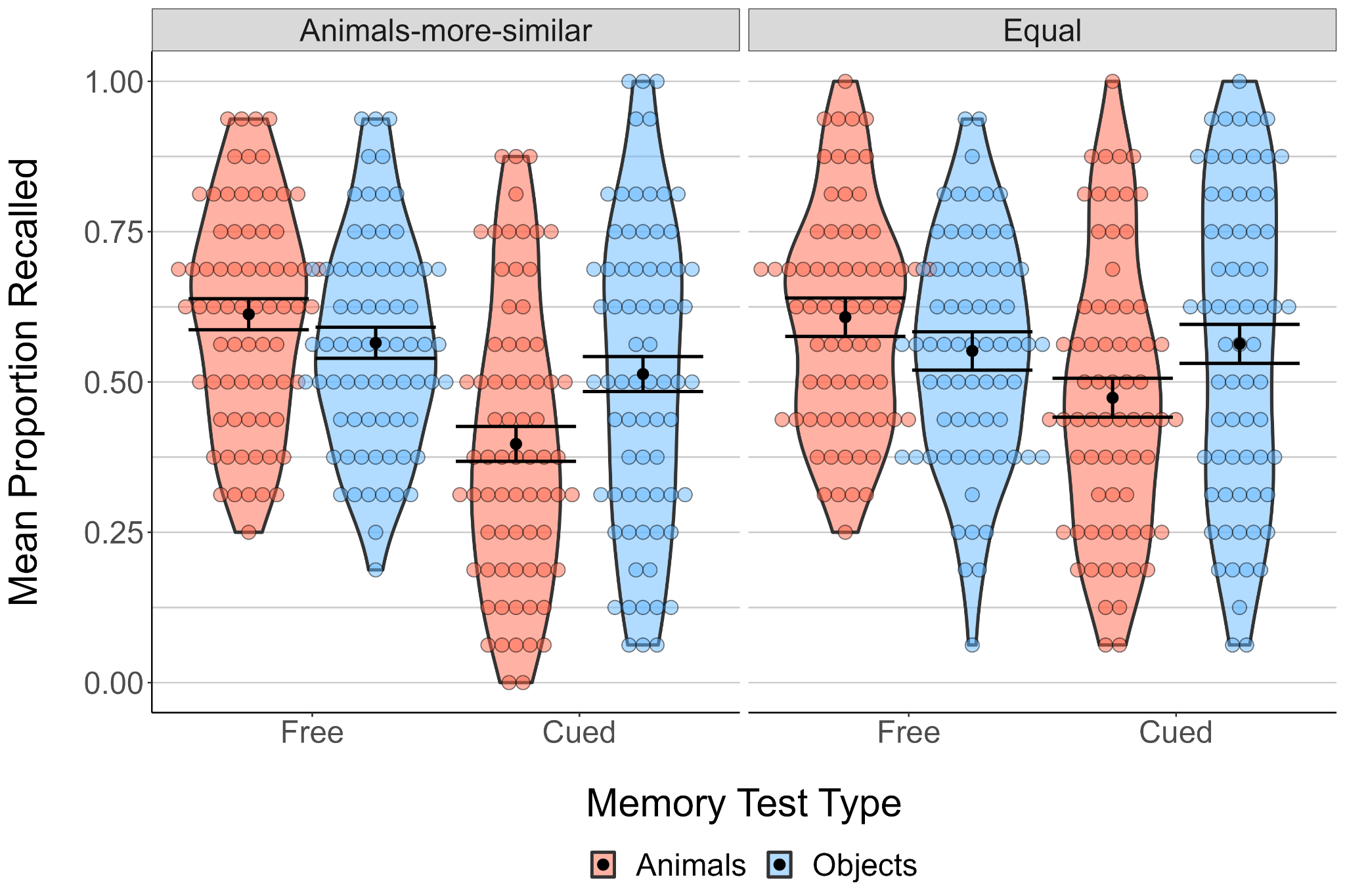


- 1. Prolific


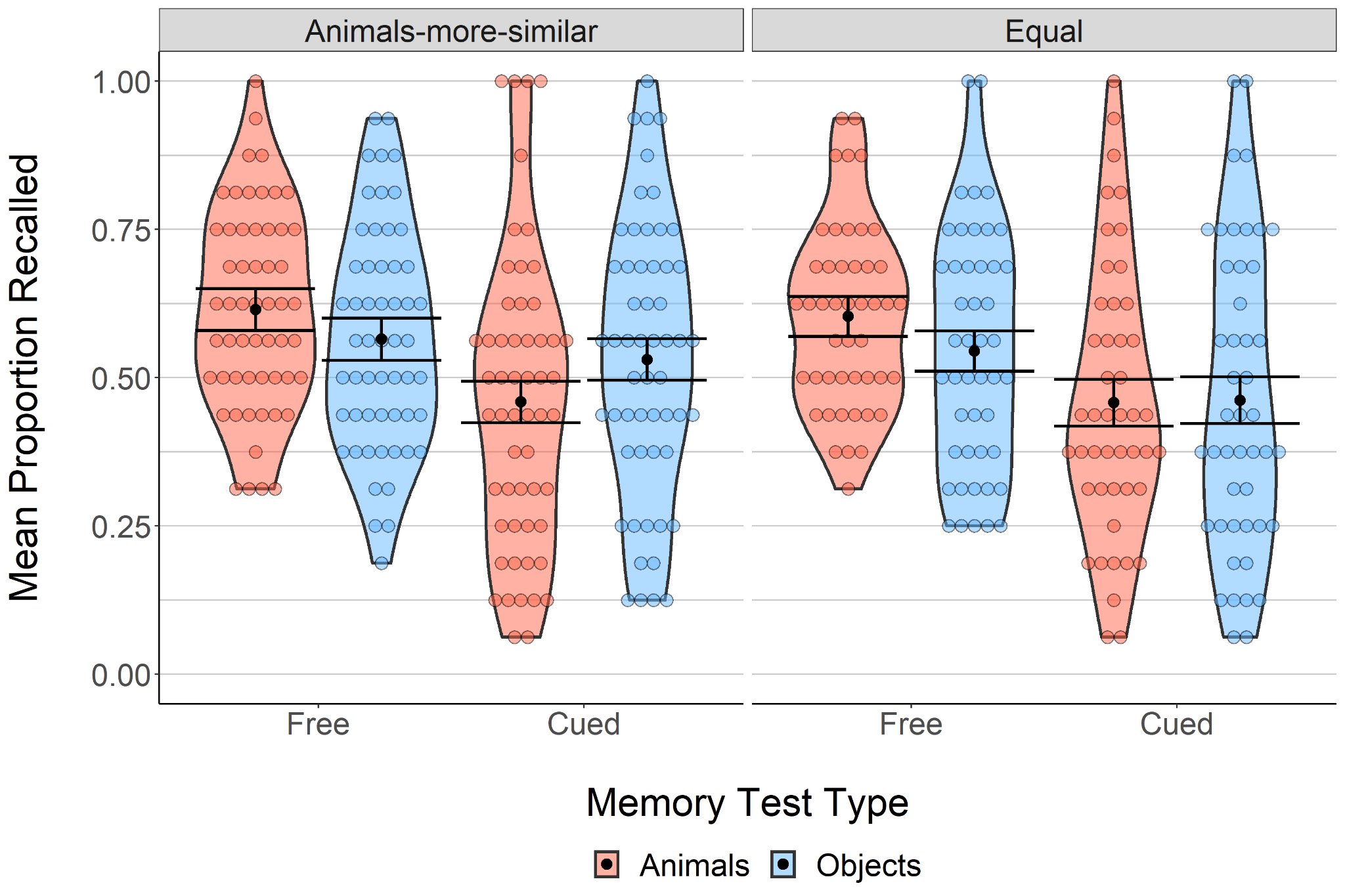


- 1. Prolific (EFL)


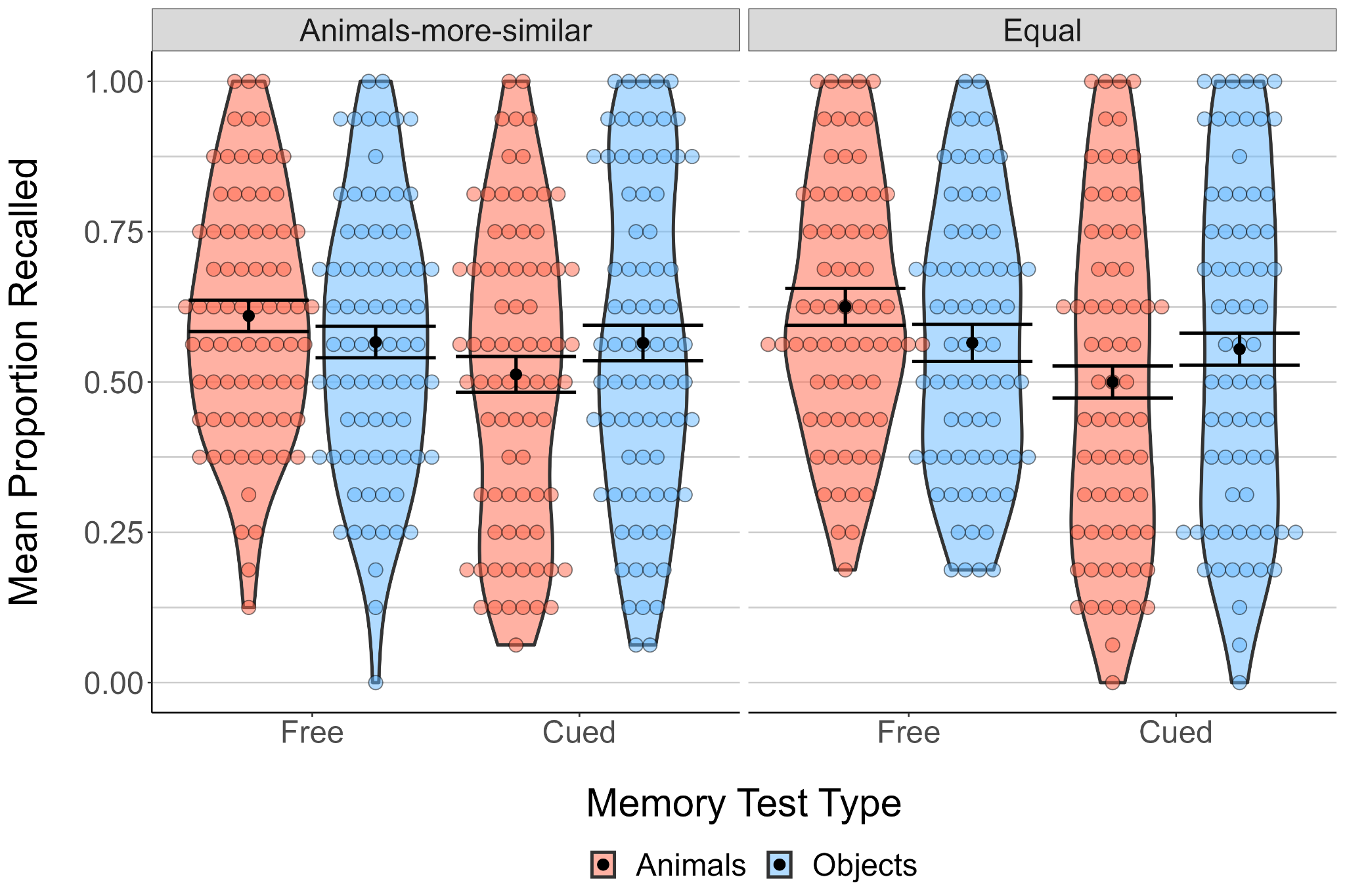


###

### Semantic similarity manipulation check

#### Distributions

##### LSA similarity


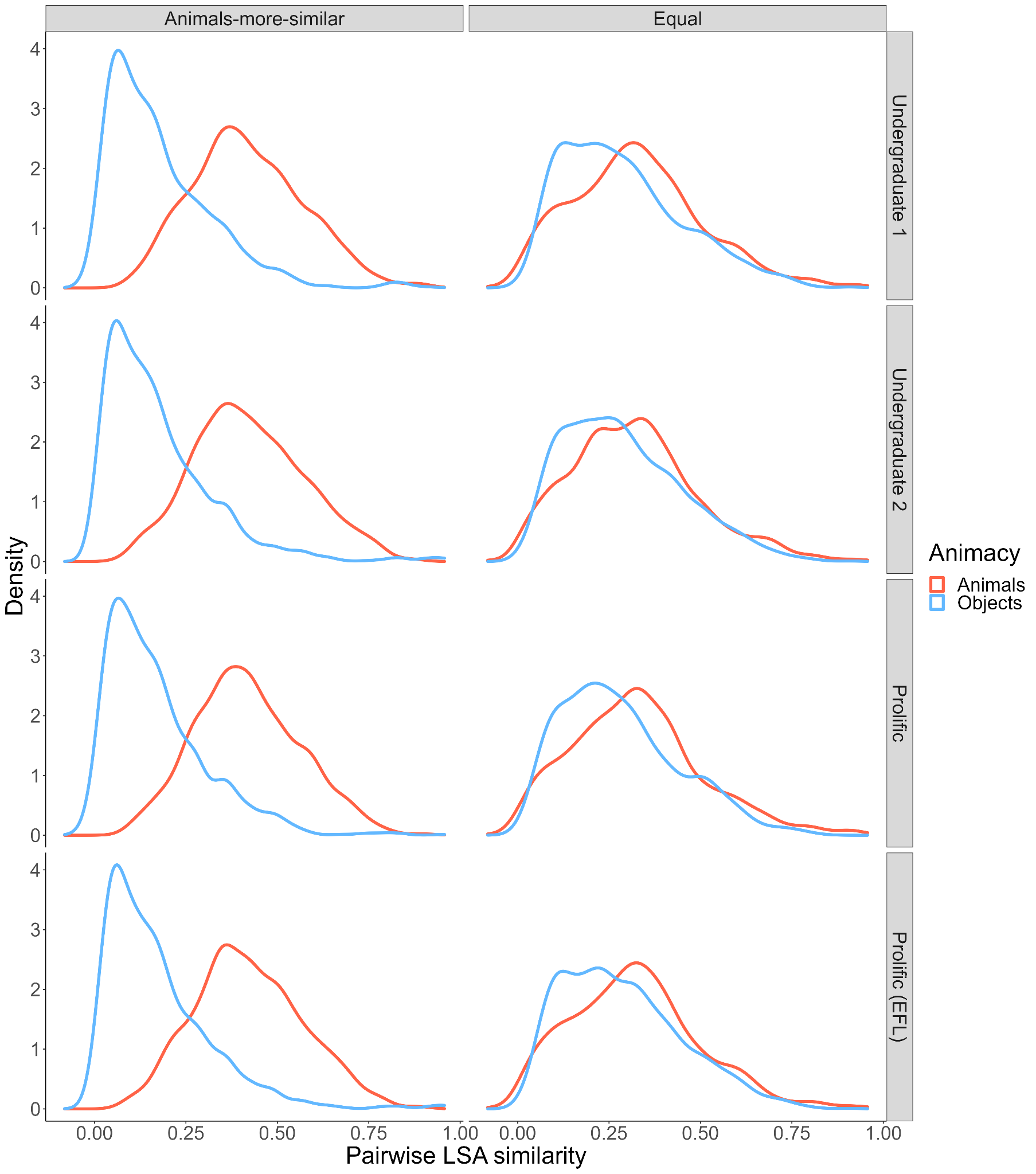


- - 1. GloVe similarity


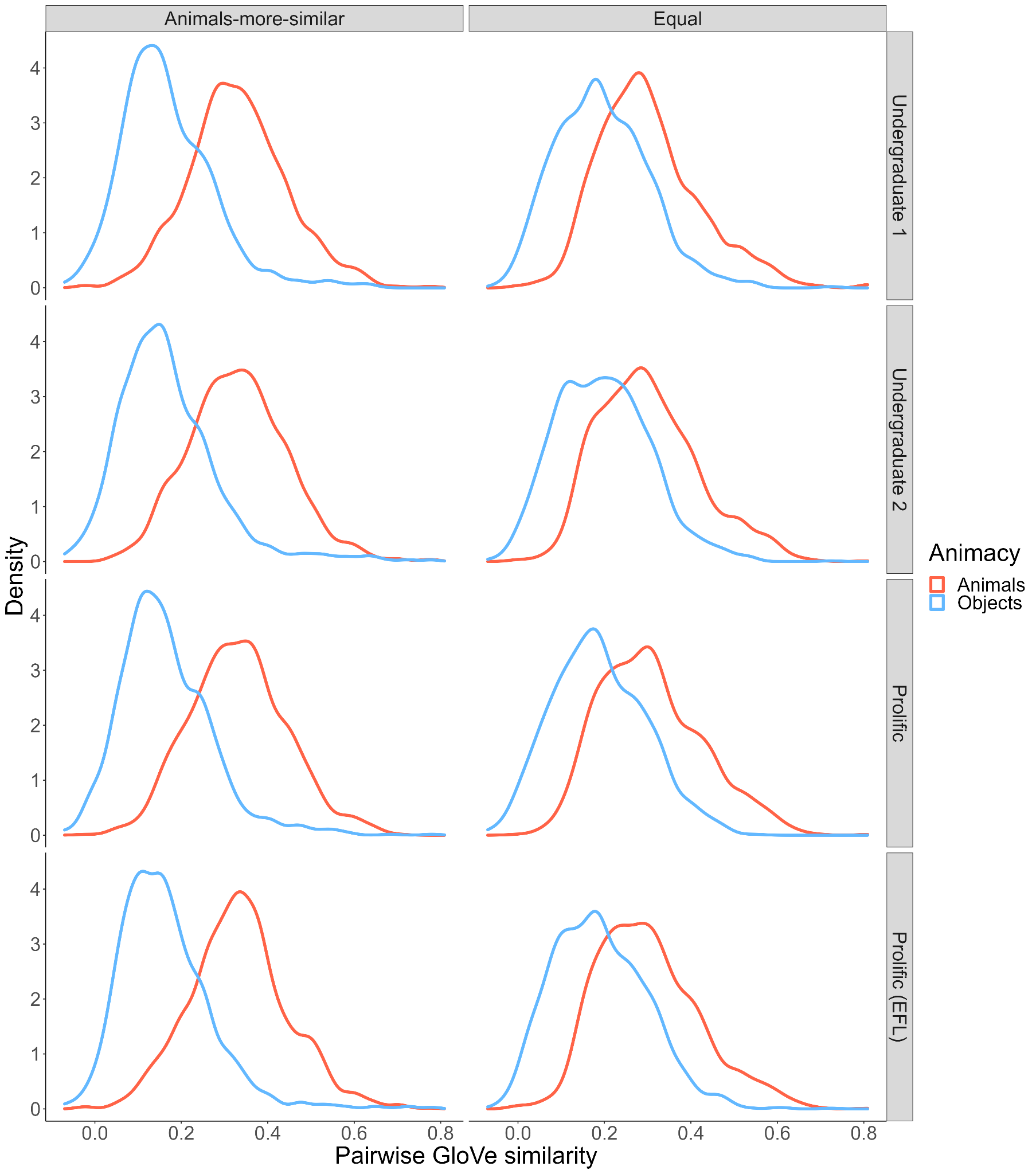


#### Predicting accuracy from semantic similarity

- - 1. Combined sample


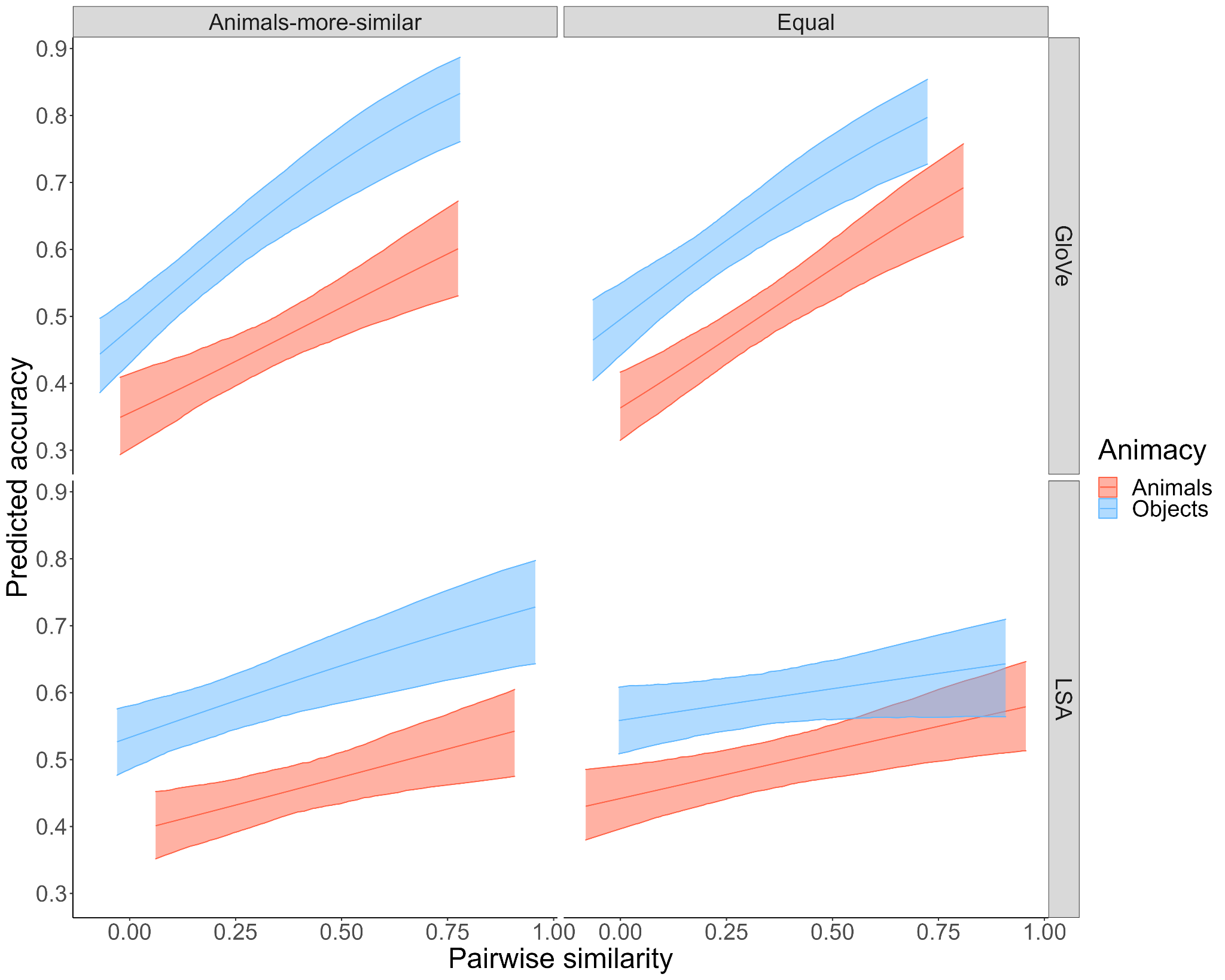


*Note.* Plots depict fixed-effects estimates and 95% confidence intervals from generalized mixed-effects linear models predicting accuracy from Similarity, Condition, and Animacy. Both similarity measures significantly predicted accuracy (LSA *p* = .004, GloVe *p* < .001). There were no significant interactions involving Similarity.

- - 1. Individual samples: LSA similarity


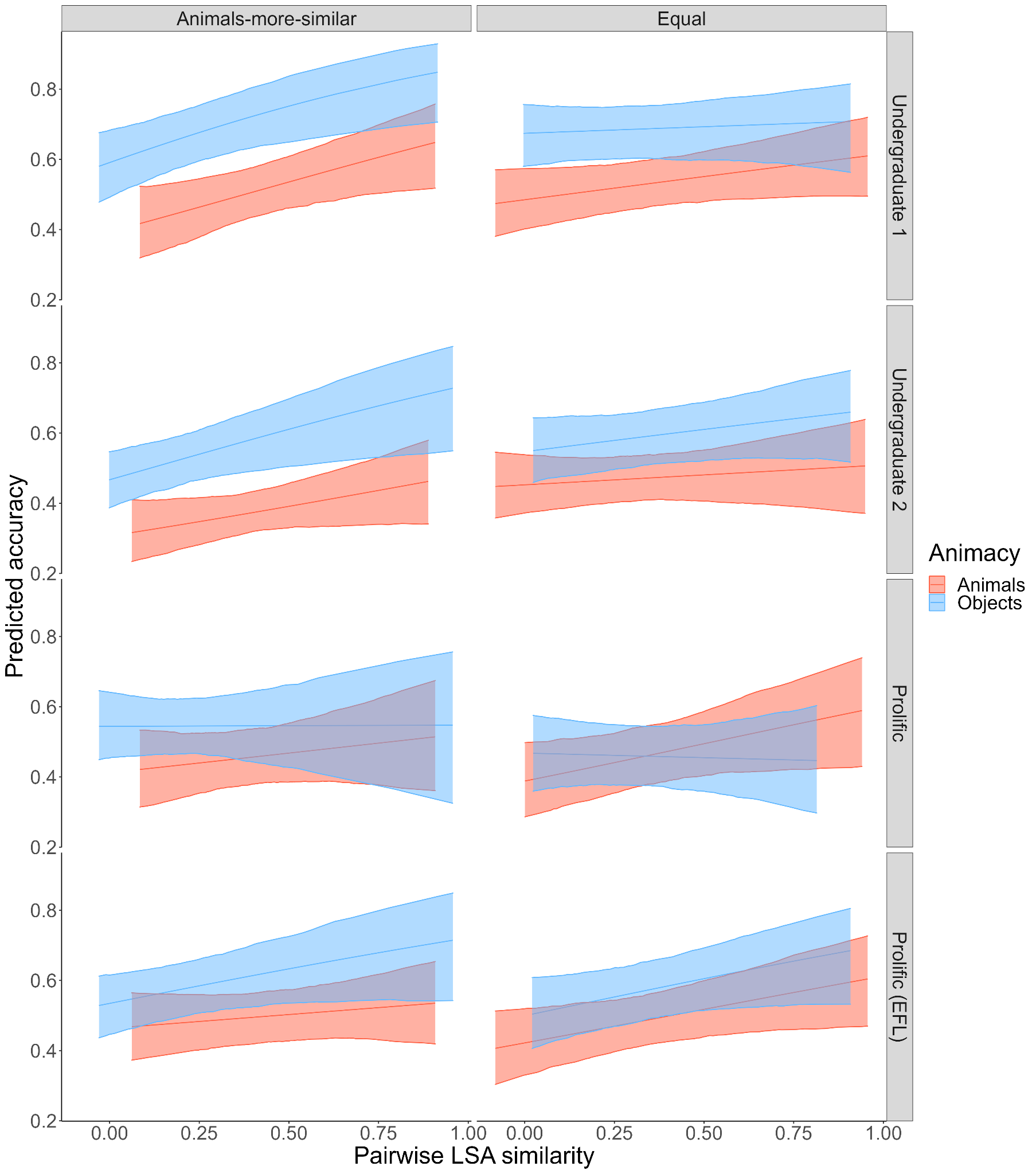


*Note.* Plots depict fixed-effects estimates and 95% confidence intervals from generalized mixed-effects linear models predicting accuracy from Similarity, Condition, and Animacy. Similarity significantly predicted accuracy in the Undergraduate 1 sample (*p* = .01). No other coefficients involving LSA similarity were significant.

- - 1. Individual samples: GloVe similarity


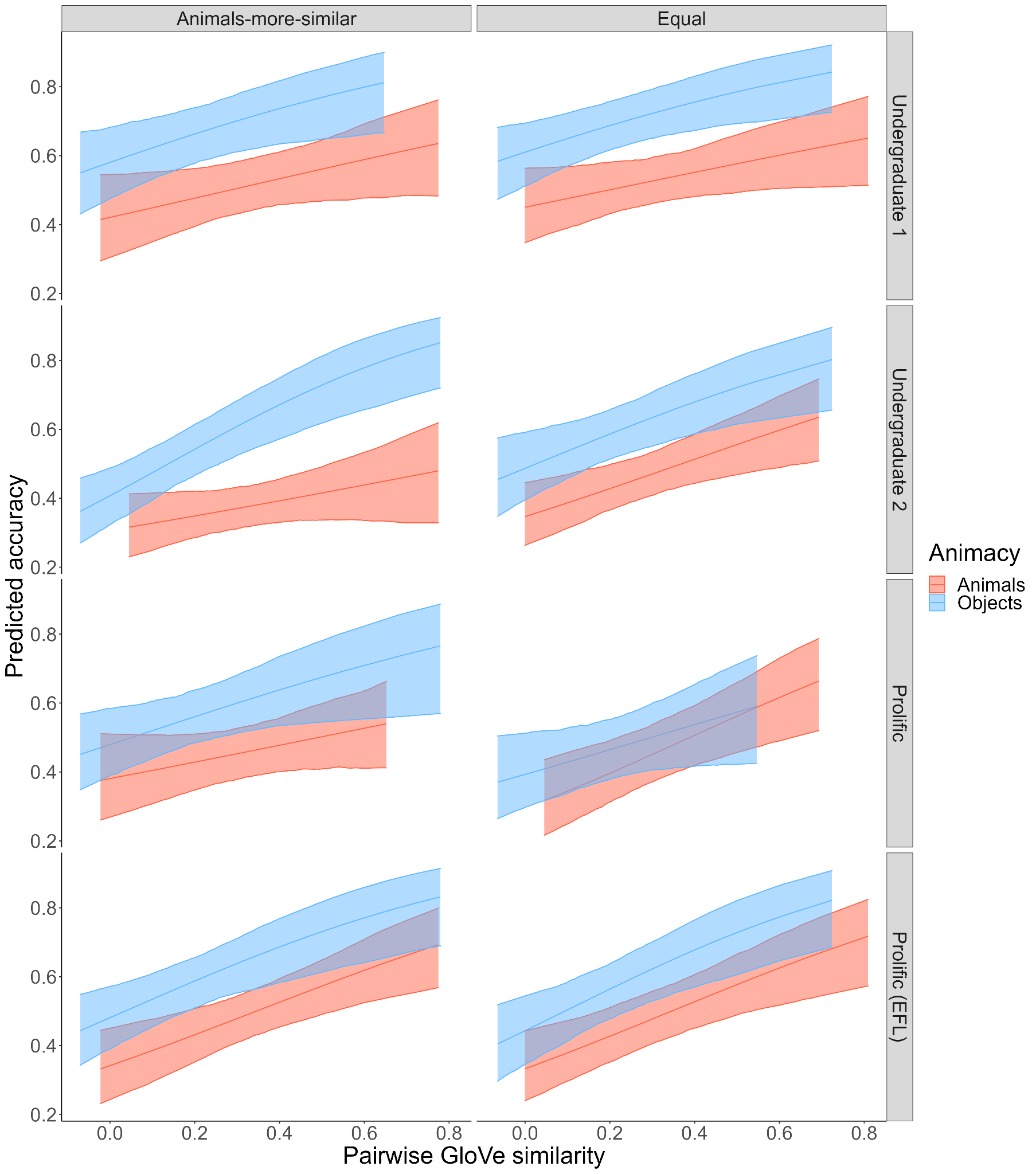


*Note.* Plots depict fixed-effects estimates and 95% confidence intervals from generalized mixed-effects linear models predicting accuracy from Similarity, Condition, and Animacy. Similarity significantly predicted accuracy in the Prolific (EFL) sample (*p* < .001). There was also a significant interaction between Animacy and Similarity in the “Animals-more-similar” condition in the Undergraduate 2 sample (*p* = .04). No other coefficients involving GloVe similarity were significant.

### Manual coding of commission errors

| Sample | Free Recall | | | Cued Recall | | |
| --- | --- | --- | --- | --- | --- | --- |
|  | Commission errors/total responses | Coder agreement | Final corrected responses | Commission errors/total responses | Coder agreement | Final corrected responses |
| Undergraduate 1 | 270/3,577 | 87% | 185 | 699/4,200 | 94% | 146 |
| Undergraduate 2 | 304/3,439 | 85% | 172 | 708/3,771 | 94% | 150 |
| Prolific | 189/2,419 | 93% | 104 | 517/2,764 | 97% | 93 |
| Prolific (EFL) | 205/3,377 | 93% | 105 | 825/5,728 | 96% | 109 |

### Main results using automatic/verbatim accuracy

|  | H1: Animacy and reverse animacy effects in the “animals-more-similar” condition | | H2: Reverse animacy effect in the “animals-more-similar” but not “equal” condition | |
| --- | --- | --- | --- | --- |
| Sample | Interaction BF10 | Interaction NHST | Interaction BF10 | Interaction NHST |
| Undergraduate 1 | 8.70 * 10^8^ | *F*(1, 67) = 22.63  *p* < .001  η^2^_p_ = .25 | .27 | *F*(1, 148) = .27  *p* = .61  η^2^_p_ = .002 |
| Undergraduate 2 | 3.57 * 10^8^ | *F*(1, 70) = 40  *p* < .001  η^2^_p_ = .36 | .29 | *F*(1, 138) = .74  *p* = .39  η^2^_p_ = .005 |
| Prolific | 1.72 * 10^7^ | *F*(1, 54) = 10.63  *p* = .02  η^2^_p_ = .16 | 3.22 | *F*(1, 99) = 2.29  *p* = .13  η^2^_p_ = .02 |
| Prolific (EFL) | 1.31 * 10^8^ | *F*(1, 78) = 15.01  *p* < .001  η^2^_p_ = .16 | .37 | *F*(1, 148) = .004  *p* = .95  η^2^_p_ < .001 |
| Combined | 1.22 * 10^23^ | *F*(1, 272) = 81.42  *p* < .001  η^2^_p_ = .23 | .27 | *F*(1, 539) = .38  *p* = .54  η^2^_p_ < .001 |

|  | “Animals-more-similar” condition | | | | “Equal” condition | | | |
| --- | --- | --- | --- | --- | --- | --- | --- | --- |
|  | FR | | CR | | FR | | CR | |
| Sample | BF10 | NHST | BF10 | NHST | BF10 | NHST | BF10 | NHST |
| Undergraduate 1 | 2.66 | *t*(67) = 2.00  *p* = .05 / .08  *D* = .24 [.001, .48] | 6,013.88 | *t*(67) = 4.81  *p* < .001  *D* = -.58  [-.84, -.32] | .22 | *t*(81) = .79  *p* = .43 / .35  *D* = .09 [-.13, .30] | 5.23 * 10^4^ | *t*(81) = 4.55  *p* < .001  *D* = -.50  [-.73, -.27] |
| Undergraduate 2 | 4.18 | *t*(70) = 2.60  *p* = .01 / .02  *D* = .31 [.07, .55] | 5.82 * 10^4^ | *t*(70) = 5.64  *p* < .001  *D* = -.67 [-.92, -.41] | 64.97 | *t*(68) = 2.48  *p* = .02 / .01  *D* = .30  [.06, .54] | 9.19 * 10^3^ | *t*(68) = 3.90  *p* < .001  *D* = -.47  [-.72, -.22] |
| Prolific | 10.24 | *t*(54) = 1.98  *p* = .05 / .04  *D* = .27 [-.003, .53] | 9.87 | *t*(54) = 2.68  *p* = .01  *D* = -.36 [-.63, -.09] | .01 | *t*(45) = 2.23  *p* = .03  *D* = .33 [.03, .62] | .02 | *t*(45) = .46  *p* = .65 / .78  *D* = -.07 [-.36, .22] |
| Prolific (EFL) | 2.83 | *t*(78) = 2.29  *p* = .02  *D* = .26 [.03, .48] | 4.96 | *t*(78) = 2.70  *p* = .008 / .009  *D* = -.30 [-.53, -.08] | 51.78 | *t*(70) = 2.79  *p* = .007 / .006  *D* = .33 [.09, .57] | 4.60 | *t*(70) = 3.11  *p* = .003 / .005  *D* = -.37 [-.61, -.13] |
| Combined | 2,706.90 | *t*(272) = 4.44  *p* < .001  *D* = .27 [.15, .39] | 5.30 * 10^10^ | *t*(272) = 7.77  *p* < .001  *D* = -.47 [-.59, -.34] | 2,560.64 | *t*(267) = 4.18  *p* < .001  *D* = .26 [.13, .38] | 5.67 * 10^8^ | *t*(267) = 6.30  *p* < .001  *D* = -.38 [-.51, -.26] |

### Semantic similarity measures and animacy effects in other wordsets

| **Wordset** | **LSA similarity Animate** | **LSA similarity Inanimate** | **GloVe similarity Animate** | **GloVe similarity Inanimate** | **LSA animacy overlap** | **GloVe animacy overlap** | **Effect summary** |
| --- | --- | --- | --- | --- | --- | --- | --- |
| Mah et al. - Animals-more-similar | 0.41 (0.15) | 0.17 (0.14) | 0.32 (0.11) | 0.17 (0.11) | 0.22 | 0.29 | Animacy disadvantage in 3/3 samples |
| Mah et al. - Equal | 0.32 (0.18) | 0.28 (0.16) | 0.31 (0.11) | 0.19 (0.11) | 0.77 | 0.43 | Animacy disadvantage in 2/3 samples, no difference in 1/3 samples |
| Popp and Serra (2016) | 0.41 (0.18) | 0.26 (0.18) | 0.37 (0.11) | 0.21 (0.12) | 0.47 | 0.3 | Animacy disadvantage in 2/3 samples, no difference in 1/3 samples |
| Popp and Serra (2018) | 0.17 (0.14) | 0.18 (0.14) | 0.18 (0.11) | 0.14 (0.11) | 0.82 | 0.75 | Animacy advantage in 2/2 samples |
| Serra & DeYoung - Atypical | 0.54 (0.24) | 0.4 (0.21) | 0.49 (0.16) | 0.42 (0.11) | 0.48 | 0.41 | Animacy advantage in 2/2 samples |
| Serra & DeYoung - Typical | 0.54 (0.23) | 0.49 (0.2) | 0.48 (0.15) | 0.56 (0.12) | 0.6 | 0.4 | Animacy disadvantage in 2/2 samples |
| Serra & DeYoung - Unrelated 1 | 0.1 (0.08) | 0.15 (0.1) | 0.12 (0.11) | 0.17 (0.11) | 0.46 | 0.53 | No difference in 1/1 sample |
| Serra & DeYoung - Unrelated 2 | 0.1 (0.07) | 0.17 (0.09) | 0.11 (0.07) | 0.21 (0.06) | 0.32 | 0.25 | No difference in 1/1 sample |

### Category Awareness data

1. Coder agreement

| Sample | Category specificity coder agreement |
| --- | --- |
| Undergraduate 1 | 71% |
| Undergraduate 2 | 86% |
| Prolific 1 | 83% |
| Prolific 2 | 84% |

1. Category awareness in each sample


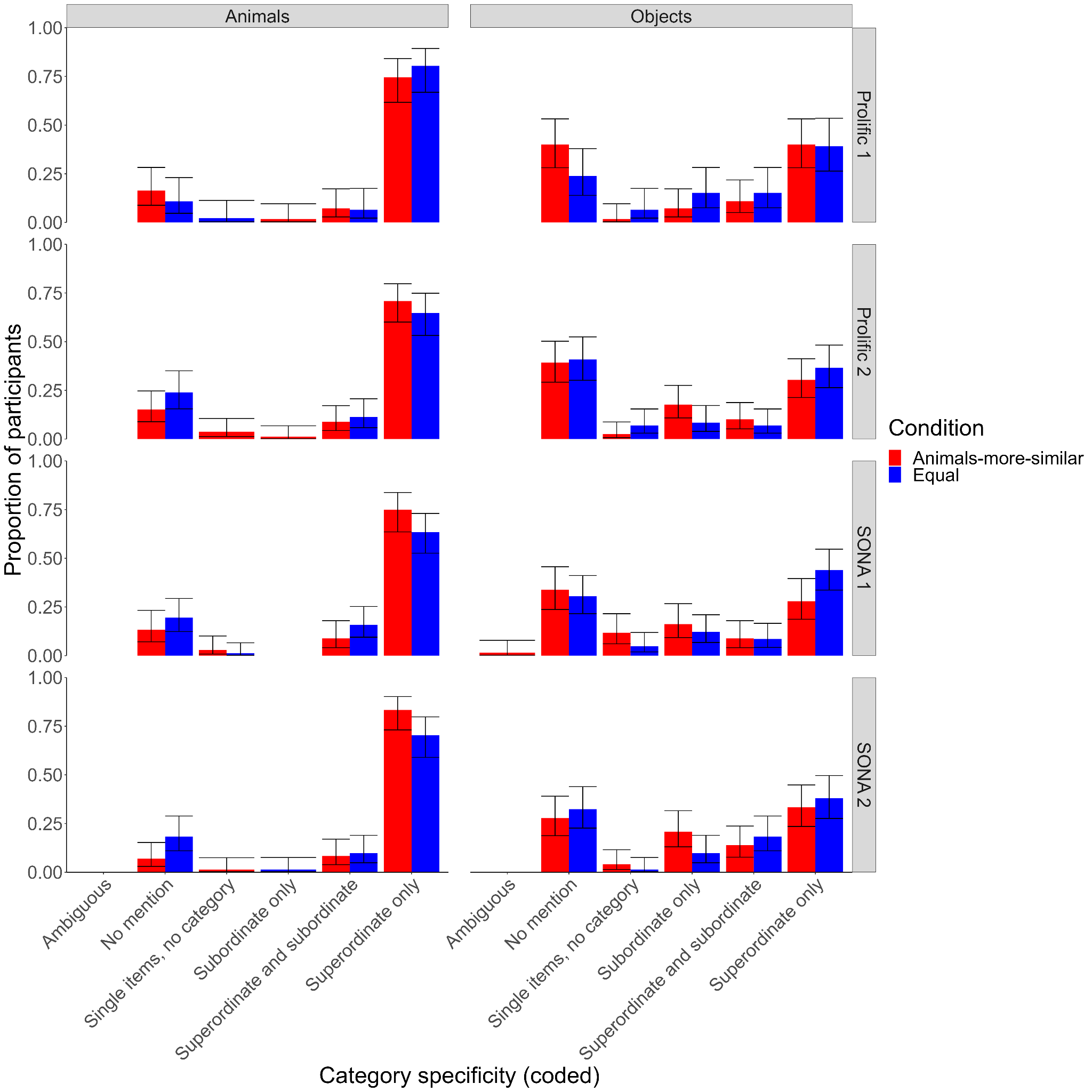


*Note.* Error bars = 95% CIs on the proportions (Wilson method). No between-condition comparisons were significant

1. Category awareness in the combined Experiment 2 data


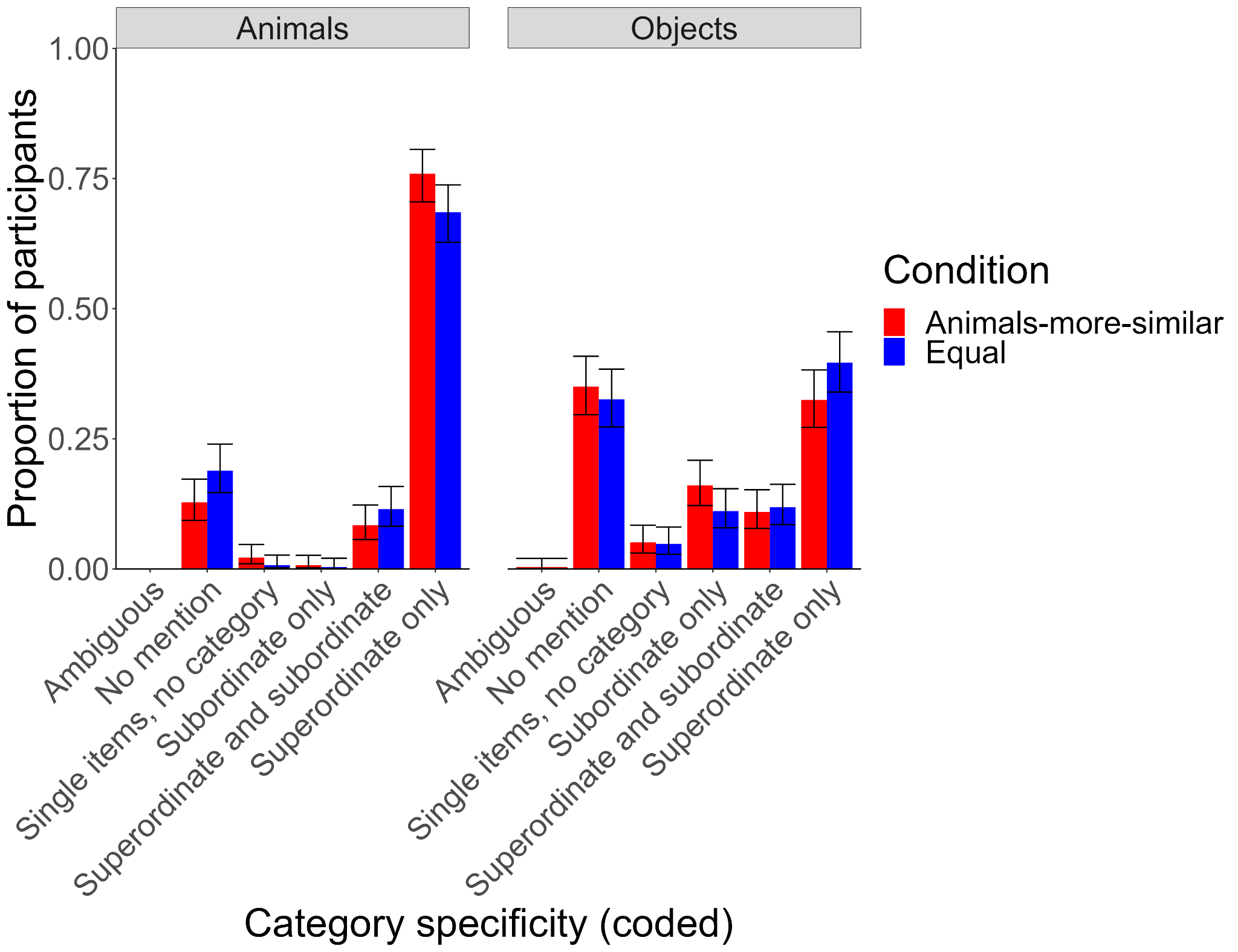


*Note.* Error bars = 95% CIs on the proportions (Wilson method). No between-condition comparisons were significant

1. Category awareness and the reverse animacy effect

If category awareness plays a role in modulating the reverse animacy effect, we might expect to see differences depending on participants’ awareness of the categories. The two most common categories in the combined Experiment 2 data were participants who indicated awareness of superordinate “animal” and “object” categories (*n* = 249, 46%) and participants who indicated awareness of a superordinate “animals” category but not a superordinate “objects” category (*n* = 198, 36%). We compared the magnitude of the CR reverse animacy effect in these two groups, but did not find a significant difference, *t*(411.39) = 1.08, *p* = .28. The figure below confirms that differences between these two groups were minimal:


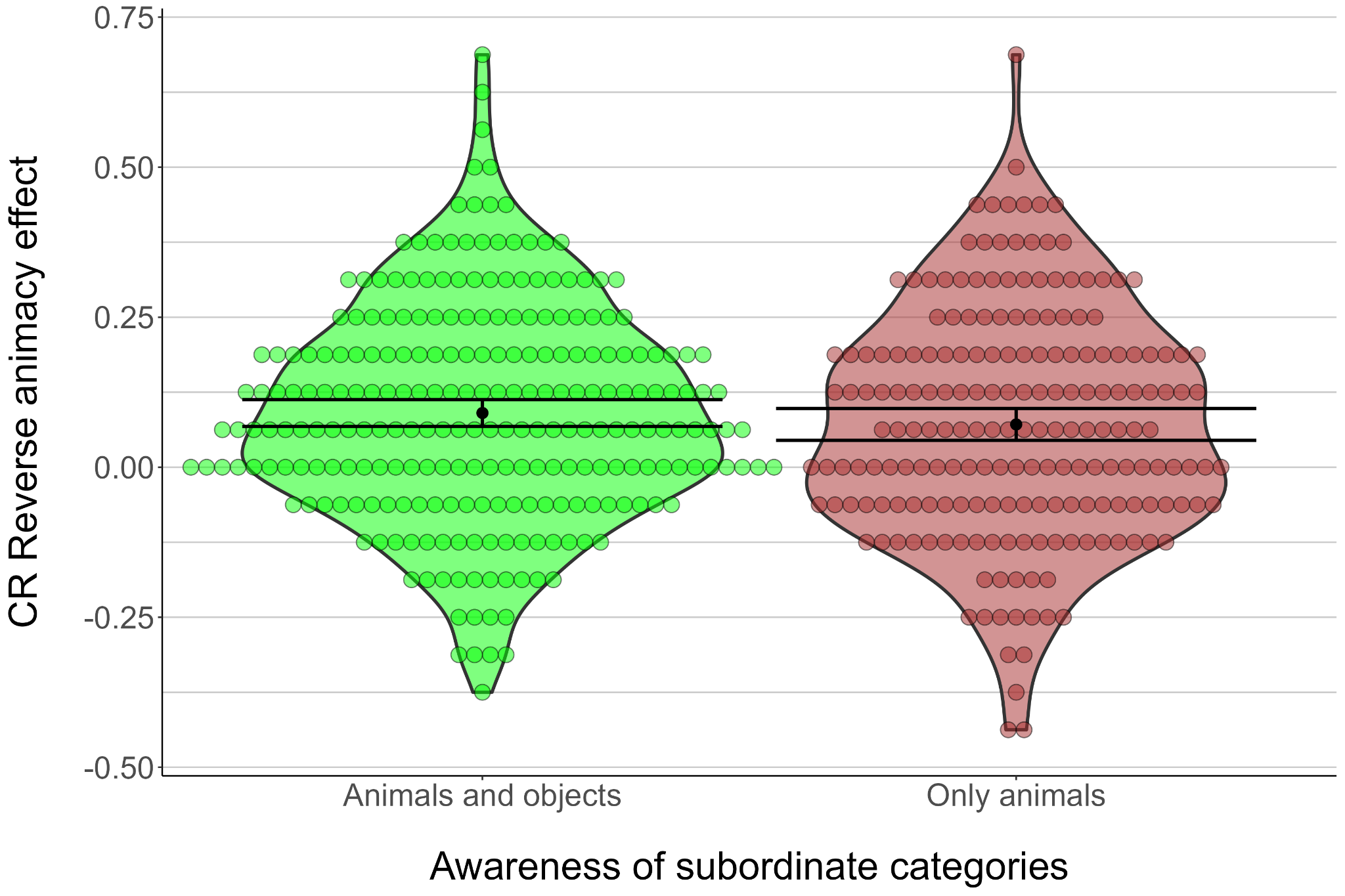


*Note.* Error bars = 95% CIs (between-subjects)

**Supplementary References**

Gronau, Q.F., Singmann, H., & Wagenmakers, E-J. (2020). bridgesampling: An R Package for Estimating Normalizing Constants. *Journal of Statistical Software, 92*(10), 1-29. doi:10.18637/jss.v092.i10 (URL: <https://doi.org/10.18637/jss.v092.i10>).

McElreath, R. (2019). *Statistical Rethinking: A Bayesian Course with Examples in R and Stan* (2nd Edition). CRC Press.

Wagenmakers, E.-J., Lodewyckx, T., Kuriyal, H., & Grasman, R. (2010). Bayesian hypothesis testing for psychologists: A tutorial on the Savage-Dickey method. *Cognitive Psychology, 60*(3), 158-189. doi: 10.1016/j.cogpsych.2009.12.001

Wagenmakers, E.-J., Wetzels, R., Borsboom, D., & Van Der Maas, H.L.J. (2011). Why psychologists must change the way they analyze their data: The case of psi: Comment on

Bem (2011). *Journal of Personality and Social Psychology, 100*(3), 426-432. doi:10.1037/a0022790

Wagenmakers, E.-J., Marsman, M., Jamil, T., Ly, A., Verhagen, J., Love, J., . . ., & Rouder, J.N. (2018). Bayesian inference for psychology: Part I: Theoretical advantages and practical ramifications. *Psychonomic Bulletin & Review, 25*, 35-57. doi 10.3758/s13423-017-

1343-3

1. Estimated using bridge sampling (Gronau et al., 2020) [↑](#footnote-ref-1)
2. All models were mixed-effects models, and included random intercepts by participant to account for the within-subjects nature of the data. [↑](#footnote-ref-2)
3. Bayesian analysis of our liberal scoring data (using the same priors as our main analysis) resulted in a Savage-Dickey BF of 5.87 *against* a reverse animacy effect. Additionally, we observed a Savage-Dickey BF > 100 *against* a reverse animacy effect similar in magnitude to the original Popp and Serra reverse animacy effect. [↑](#footnote-ref-3)
4. For both t-tests, corresponding Wilcoxon tests returned similar nonsignificant results. [↑](#footnote-ref-4)
5. *df* adjusted to account for unequal variances via Welch’s t-test. [↑](#footnote-ref-5)
6. *df* adjusted to account for unequal variances via Welch’s t-test. [↑](#footnote-ref-6)
7. Note that for this analysis, we converted animacy effects from proportions to percentages to allow for more intuitive specification for the prior on the continuous animacy effect. [↑](#footnote-ref-7)
8. A corresponding Wilcoxon signed-rank test was conducted, with similar results to the parametric t test. [↑](#footnote-ref-8)
9. The temporal order in which these samples were collected was: Undergraduate 1, Prolific, Prolific (EFL), Undergraduate 2. [↑](#footnote-ref-9)
